# A Robust Workflow for Acquiring and Preprocessing Ambient Vibration Data from Small Aperture Ocean Bottom Seismometer Arrays to Extract Scholte and Love Waves Phase-Velocity Dispersion Curves

**DOI:** 10.1007/s00024-021-02923-8

**Published:** 2021-12-14

**Authors:** Agostiny Marrios Lontsi, Anastasiia Shynkarenko, Katrina Kremer, Manuel Hobiger, Paolo Bergamo, Stefano C. Fabbri, Flavio S. Anselmetti, Donat Fäh

**Affiliations:** 1 Swiss Seismological Service, Swiss Federal Institute of Technology (ETH), Sonneggstrasse 5, 8092 Zurich, Switzerland; 2 Institute of Geological Sciences and Oeschger Centre for Climate Change Research, University of Bern, Baltzerstrasse 1+3, 3012 Bern, Switzerland; 3 Federal Institute for Geosciences and Natural Resources (BGR), Stilleweg 2, 30655 Hanover, Germany

**Keywords:** Offshore ambient vibrations, OBS localization, OBS clock error, OBS misorientation, Scholte waves, Love waves, Phase-velocity dispersion curves

## Abstract

The phase-velocity dispersion curve (DC) is an important characteristic of the propagation of surface waves in sedimentary environments. Although the procedure for DC estimation in onshore environments using ambient vibration recordings is well established, the DC estimation in offshore environments using Ocean Bottom Seismometers (OBS) array recordings of ambient vibrations presents three additional challenges: (1) the localization of sensors, (2) the orientation of the OBS horizontal components, and (3) the clock error. Here, we address these challenges in an inherent preprocessing workflow to ultimately extract the Love and Scholte wave DC from small aperture OBS array measurements performed between 2018 and 2020 in Lake Lucerne (Switzerland). The arrays have a maximum aperture of 679 m and a maximum deployment water depth of 81 m. The challenges related to the OBS location on the lake floor are addressed by combining the multibeam bathymetry map and the backscatter image for the investigated site with the differential GPS coordinates of the OBS at recovery. The OBS measurements are complemented by airgun surveys. Airgun data are first used to estimate the misorientation of the horizontal components of the OBS and second to estimate the clock error. To assess the robustness of the preprocessing workflow, we use two array processing methods, namely the three-component high-resolution frequency-wavenumber and the interferometric multichannel analysis of surface waves, to estimate the dispersion characteristics of the propagating Scholte and Love waves for one of the OBS array sites. The results show the effectiveness of the preprocessing workflow. We observe the phase-velocity dispersion curve branches in the frequency range between 1.2 and 3.2 Hz for both array processing techniques.

**Supplementary Information:**

The online version contains supplementary material available at 10.1007/s00024-021-02923-8.

## Introduction

As part of the initiative to assess the hazard of earthquake-induced tsunamis at Lake Lucerne (Switzerland), an extensive Ocean Bottom Seismometer (OBS) campaign was carried out to measure seismic ambient vibrations (and earthquakes) on subaqueous slopes. Some of the selected subaqueous slopes for OBS array deployments are well-known to have failed in the past (Schnellmann et al. 2003; Hilbe et al. 2011; Hilbe and Anselmetti 2015). Other slopes may have been weakened by previous seismic or aseismic loadings and could therefore fail spontaneously or in case of further external triggering mechanisms such as an earthquake.

Common approaches for subaqueous slope-stability analysis include the multibeam bathymetry (MB) for geomorphometric studies, reflection seismic and geotechnical investigations (e.g. coring, cone penetration testing) to assess the sediment volume and the mechanical properties of the sediments that have failed or are susceptible to failure (e.g. Urgeles et al. 2006; Strasser et al. 2007; Sammartini et al. 2021). Controlled-source surface-wave surveys using airgun are also used to estimate the phase-velocity dispersion curve (DC) of Scholte waves for the underwater subsurface sedimentary cover (Nolet and Dorman 1996; Bohlen et al. 2004; Vanneste et al. 2011). However, depending on the stiffness of the sediments and the characteristics of the seismic source used in reflection seismics and active surface-wave experiments, or the sediment coring equipment, the investigation depth is generally limited to the first few meters of the subsurface.

Passive seismic measurements, on the other hand, provide data that can span a broader frequency range, therefore allowing us to investigate subaqueous slopes on a broad depth range. With the advances in offshore seismic instrumentation, that include for example Ocean Bottom Seismometers (Webb 1998; Stähler et al. 2016; Hannemann et al. 2017; Shynkarenko et al. 2021), high quality seismic data are emerging from the offshore environments for subsurface structure analysis. These data aid us to characterize the sediments of the lake/ocean bottom and to assess the volume of sediment prone to failure. Although the operation of OBS devices is relatively challenging in terms of logistic and safety measures involved, they allow us to extract not only the Scholte wave phase velocity dispersion curves, but also the dispersion characteristic of Love waves (Shynkarenko et al. 2021). In addition, single-station three-component methods (Nakamura 1989; Bard 1998; Bonnefoy-Claudet et al. 2006) applied to OBS data allow us to easily extract the Scholte wave ellipticity and the horizontal-to-vertical (H/V) spectral ratio. The H/V provides information about the resonance frequency of the submerged slope (Gomberg 2018; Courboulex et al. 2020; Shynkarenko et al. 2021).

In onshore environments, array methods to process ambient vibration recordings for DC include for example the SPatial AutoCorrelation (SPAC; Aki 1957; Bettig et al. 2001), the frequency-wavenumber technique (Capon 1969; Poggi and Fäh 2010), the wavefield decomposition technique (WaveDec; Maranò et al. 2012), the interferometric multichannel analysis of surface waves (IMASW; Gouédard et al. 2008; Cheng et al. 2015; Lontsi et al. 2016), and the multichannel analysis of surface waves (MASW; Park et al. 1999). These methods were fully exploited for an integrated analysis of the surface-wave phase-velocity dispersion curves on a broad frequency range (e.g. Lontsi et al. 2016; Hobiger et al. 2021). The DC and the Rayleigh wave ellipticity or the microtremor H/V can further be jointly inverted to estimate the subsurface structure in onshore environments (e.g. Scherbaum et al. 2003; Hobiger et al. 2013; Lontsi et al. 2016).

In offshore environments, the DC estimation presents additional challenges that include the determination of the precise location of the OBS on the lake floor, the sensor orientation, i.e. the estimation of the misorientation of the horizontal components with respect to the geographic north, also referred to as sensor orientation, and the clock error correction. These challenges need to be addressed before applying conventional array methods. Here, we present a qualitative description of the data acquisition and a detailed quantitative analysis of the preprocessing steps that were developed to process small aperture OBS array data from Lake Lucerne and that ultimately lead to obtain clear DC. For testing the robustness of the preprocessing workflow, the three-component High Resolution Frequency-Wavenumber (3C-HRFK; Poggi and Fäh 2010) and the Interferometric Multichannel Analysis of Surface Waves (IMASW; Lontsi et al. 2016) techniques are applied to the data of one OBS array site. The choice for the two techniques is motivated by the capability of the 3C-HRFK to separate waves with close wavelength and the capability of the IMASW to exploit passive data enriched with low frequency using signal processing techniques for active data. Finally, obtained phase velocity dispersion curves for Scholte and Love waves are picked to be further inverted with additional constrains for the subsurface structure. The dispersion curve estimation at additional OBS array sites and their inversion results are presented in Shynkarenko et al. (2021).

## Geological Setting, Site Selection and Instruments

### Geological Setting of the Lake and Site Selection

**Fig. 1:**
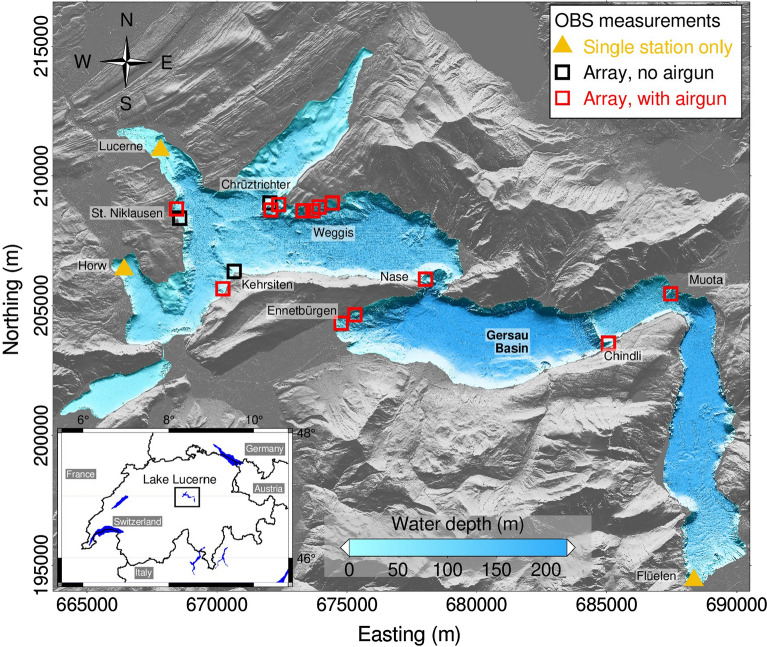
Single station and array measurement sites in Lake Lucerne. The three triangle markers at Horw, Lucerne, and Flüelen indicate sites with OBS single station measurements only. The red and black square markers indicate OBS array measurements sites with and without additional airgun surveys, respectively. The coordinates are displayed using the Swiss Coordinate System LV03 in m. The background layers represent the topography around the lake (gray) and the bathymetry of Lake Lucerne. The inset indicates the location of Lake Lucerne within Switzerland (Source: Federal Office of Topography)

Lake Lucerne is a fjord-type lake situated in the perialpine region in central Switzerland. Figure 1 gives an overview of the lake and the deployment locations for the OBS stations. The lake is characterized by an elongated shape between steep rock masses and the presence of moraine ridges between the different lake basins. A summary of the main geological and morphological features of the lake can be found in Hilbe et al. (2011). The predominant elongated shape of the glacially overdeepened Lake Lucerne basins is caused by the erosive power of the Reuss and Engelberg Glaciers and by the Brünig branch of the Aare Glacier during the Quaternary glaciations (Preusser et al. 2010). The present-day lake covers an area of 114 $\hbox {km}^2$. The maximum water depth, in the Gersau Basin, is 214 m (e.g. Hilbe and Anselmetti , 2015). The lake contains Quaternary sediment deposits with the thickness ranging from few meters to hundreds of meters (Finckh et al. 1984; Hilbe et al. 2011; Shynkarenko et al. 2021).

We selected 11 sites for OBS measurements within the lake (from west to east): Horw, St. Niklausen, Lucerne, Kehrsiten, Chrüztrichter, Weggis, Nase, Ennetbürgen, Chindli, Muota, and Flüelen (Fig. 1). The selected slopes are characterized by relatively homogeneous and flat surface geomorphology and an undisturbed sedimentary cover.

### Instrumentation

Two OBS types are used in the lake: the first type is called LOBSTER (Longterm Ocean Bottom SeismomeTER) and belongs to the DEPAS (German Instrument Pool for Amphibian Seismology), and the second type is called NAMMU and belongs to the Swiss Seismological Service at ETH Zurich. Both OBS types are equipped with popup buoy, bucket, rope, syntactic foam, releaser, hydrophone, batteries, datalogger, anchor, frame, and a self-level Trillium compact 120 s seismometer (see the annotated OBS in Fig. A.1, supplementary information). The NAMMU has a smaller size and weight than the LOBSTER (Table 1). In salty water, both instruments have the same effective weight (equivalent to an apparent mass of 30 kg). The 3C-seismometer of both the LOBSTER and the NAMMU uses the right-hand system with the vertical component “1” (or “Z”) pointing up. The horizontal components are labelled “2” (or “X”) and “3” (or “Y”). A dual-degree-of-freedom motorized gimbals and jam-free mechanical kinematic design preserves full seismometer performance. The gimbal system ensures that the vertical component is always vertical and orthogonal to the two mutually perpendicular horizontal components.

**Table 1:** Physical parameters of the OBS (LOBSTER and NAMMU) used in the lake

Parameters	LOBSTER OBS	NAMMU OBS
Size (mm)	1650 x 1300 x 720	800 x 600 x 600
Mass (kg)	405	250
Frame	Titanium	
	Buoyancy from syntactic foam	
Data Logger	Seismic Data Logger “6D6”	
Releaser	K/MT 562 KUMQuat, titanium casing	
Timing	UHURA, default signal from US GPS-system, GLONASS	
Hydrophone	HTI-04-PCA/ULF	
Seismometer	Broadband Seismometer	
	Nanometrics “Trillium Compact 120 s”	
Anchor	Steel	

## OBS Deployment and Location Procedures

### OBS Deployment

The OBS were deployed at sites with relatively flat surface morphology. Prior to the array campaign, we used the NAMMU to inspect the capability of the lake floor to accommodate the sensor for the long-term deployment, and to quality-check (e.g. noise level, frequency content) the recorded data. During this site-inspection phase, data were recorded at six different sites (Chrüztrichter, Ennetbürgen, Kehrsiten, St. Niklausen (x2), Weggis). Following our protocol, the OBS started the free descent as stand-alone system to the lake floor only after a number of steps had been cross-checked. A communication between the OBS and the field laptop was established via a junction box called DIRK. From DIRK, a LAN or WLAN connection was established. Therefore, prior to the start of the recording, a web interface allowed us to check the recorder serial number, format the memory stick, set the number of recording channels, the sampling frequency (set to 250 samples per second for all OBS), and synchronize the OBS clock with the GPS. In addition, the physical environmental conditions in the recorder casing such as the specific humidity, and the temperature were checked, as well as the battery voltage. After the recording was started, we checked that both the hydrophone and the three seismometer components record the seismic ambient noise on the platform. After this step, the releaser’s acoustic communication link was disabled to ensure that the OBS does not respond to any incoming signal. Last, the screws of the seismometer and the coupling with the anchor were cross-checked on the pontoon and the OBS was deployed to the lake floor.

The array geometry was designed to take full advantage of the nine available OBS while ensuring a good array resolution. The arrays consisted of three concentric rings with three stations each, where the OBS of each ring were deployed aiming at an equilateral triangle, resulting in a spiral arm configuration. The different rings were rotated by 30–35$^\circ$ with respect to each other and the radii of consecutive rings had a ratio between 2.2$-$ 2.5. The maximum array aperture was chosen such that all stations were deployed on slopes with presumed homogeneous sediment cover. The station names of the arrays were set to have three characters and two digits. The first two characters were set to correspond to the first two initials of the surveyed site, or occasionally the first and the third initial when the previous setting was already used. The third character indicated the array configuration at the site. When applicable, the letters A, B, C, and D were used in that order as indicator for the first, second, third, and fourth configurations, respectively. In the particular case of a single station measurement only, the letter S was used. The last two-digit of the station name served as station identifier. Each array consisted of 9 OBS stations (8 LOBSTER and 1 NAMMU), except one array that consisted of 8 stations (7 LOBSTER and 1 NAMMU). The arrays St. Niklausen B (hereafter NIB), Chrüztrichter B (CHB), and Weggis B (WEB) were obtained from the previous array settings NIA, CHA, and WEB by relocating the three most inner or outer OBS stations, resulting in 6 co-located stations. In total, 17 arrays were performed. Table 2 gives a summary of the array measurement sites, a list of the array setups (A, B, C, D) with the corresponding minimum and maximum interstation distances, the minimum and maximum water depths, and the recording duration. For each array, the table also gives the start and end recording times. At 13 array locations, the OBS measurements were complemented with airgun surveys. The number of airgun shots per array, where available, is also given in Table 2. See Fig. 1 for the OBS array locations. Data examples for the different preprocessing steps (location, misorientation and clock error correction) include OBS array measurements at Muota, Chrüztrichter, and Ennetbürgen.

**Table 2:** Overview of the array measurements. The arrays marked with $^*$ share six co-located stations with the previous array at the site

Site	Array name	Inter-station min–max (m)	Water depth min–max (m)	Common start time (UTC)	Common end time (UTC)	Common duration (dd hh mm ss)	Airgun shots (nbr.)
Chrüztrichter	CHA	61.1–499.9	21.5–37.5	2018-05-08 09:46:12	2018-07-02 10:27:12	55d 00 h 41 m 00 s	0
	$\hbox {CHB}^*$	21.8–199.8	21.5–37.5	2018-07-03 08:10:41	2018-07-05 07:36:49	1d 23 h 26 m 08 s	23
	CHC	8.6–210.3	32.8–39	2019-08-20 11:03:23	2019-08-22 11:12:32	2d 00 h 09 m 09 s	139
Chindli	CIA	11.3–177.6	47.3–61.1	2018-09-14 09:32:40	2018-09-17 08:15:48	2d 22 h 43 m 08 s	23
Ennetbürgen	ENA	12.6–152.5	30.9–41.4	2018-09-11 12:55:02	2018-09-13 07:55:19	1d 19 h 00 m 17 s	28
	ENB	19.3–217.2	19.2–42.8	2019-06-27 09:39:23	2019-08-19 11:33:08	53d 01 h 53 m 45 s	175
Kehrsiten	KEA	13.5–184.6	5.1–21.5	2018-07-06 10:44:53	2018-07-09 07:50:53	2d 21 h 06 m 00 s	0
	KEB	18.9–132.0	8.5–23.6	2019-08-23 09:32:22	2019-08-26 07:51:31	2d 22 h 19 m 09 s	134
Muota	MUA	32.4–124.1	38.9–61.1	2019-06-19 10:12:20	2019-06-24 10:08:15	4d 23 h 55 m 55 s	132
Nase	NAA	28.5–229.8	36.3–66.4	2019-06-25 13:01:01	2019-06-26 08:24:00	0d 19 h 22 m 59 s	185
St. Niklausen	NIA	10.0–79.2	18.5–25.9	2018-05-02 08:42:19	2018-05-04 07:54:02	1d 23 h 11 m 43 s	0
	$\hbox {NIB}^*$	20.7–155.2	18.5–30.5	2018-05-04 10:02:00	2018-05-07 07:57:11	2d 21 h 55 m 11 s	0
	NIC	19.6–168.2	21.1–42.3	2019-09-20 10:58:04	2019-09-23 08:07:49	2d 21 h 09 m 45 s	125
Weggis	WEA	11.7–228.2	21.4–46	2018-07-10 08:30:55	2018-07-12 07:20:58	1d 22 h 50 m 03 s	29
	$\hbox {WEB}^*$	69.3–678.9	17–81.3	2018-07-12 11:00:15	2018-09-10 10:27:56	59d 23 h 27 m 41 s	21, 29, 23
	WEC	15.7–216.4	19.9–39.2	2019-08-28 08:31:43	2019-09-16 11:58:21	19d 03 h 26 m 38 s	96
	WED	14.7–188.5	22.5–47.9	2019-09-17 09:39:23	2019-09-19 07:55:43	1d 22 h 16 m 20 s	175

### OBS Location Procedure

The coordinates of the OBS were measured at deployment with a differential GPS (dGPS). These coordinates were indicative for the post-deployment multibeam survey and interpretation.

Multibeam bathymetry data were acquired with a Kongsberg EM2040 echo sounder in a 1$^\circ$ by 1$^\circ$ beam-width configuration with a 300 kHz operating frequency. Auxiliary sensors such as (i) a Seatex MRU5+ motion sensor for wave-induced motion compensation of the boat, (ii) a Trimble SPS361 heading sensor for the orientation and (iii) a Leica GX1230 GNSS receiver using the swiposGIS/GEO real-time kinematic for accurate positioning to 2–3 cms were used. A Valeport MiniSVS sound velocity sensor monitored permanently the sound speed close to the echo sounder. A vertical sound-velocity profile was recorded at the survey site with a Valeport MiniSVP probe to determine the refraction angles of the received acoustic lake-bottom reflections. Our vertical sound velocity profiles show for instance 1475.4 m/s at NIC, in 20 cm water depth, decreasing down to 1434.4 m/s in 42.3 m water depth (depth of deepest OBS at NIC). We have a measurement every 20 cm and the average value of the entire water column down to 42.3 m is 1453.0 m/s and the median is about 1447.0 m/s. These values vary slightly with season, location and height of the water column. Another example at KEB, down to 35 m water depth, shows an average sound velocity of 1456.6 m/s and a median of 1455.6 m/s. The range of these average values is small, and after comparing several profiles of various locations, we assume an average sound velocity profile of 1450 m/s for all OBS sites with an error of +/- 10 m/s. The recorded raw data have been processed using the Caris HIPS/SIPS 9.1 software. During processing, all auxiliary sensor data were merged, reviewed, and manually corrected, if necessary. The point clouds were reviewed and different algorithms for rasterizations of the point clouds were tested. The resulting digital bathymetric map has a cell size of 0.5 m with a vertical accuracy of a few centimeters and holds detailed morphological information, allowing the identification of objects such as the OBS stations on the ground. Calculated backscatter data provide additional information on the hardness of the ground, therefore providing additional constraints for the identification of the OBS on the lake floor.

**Fig. 2:**
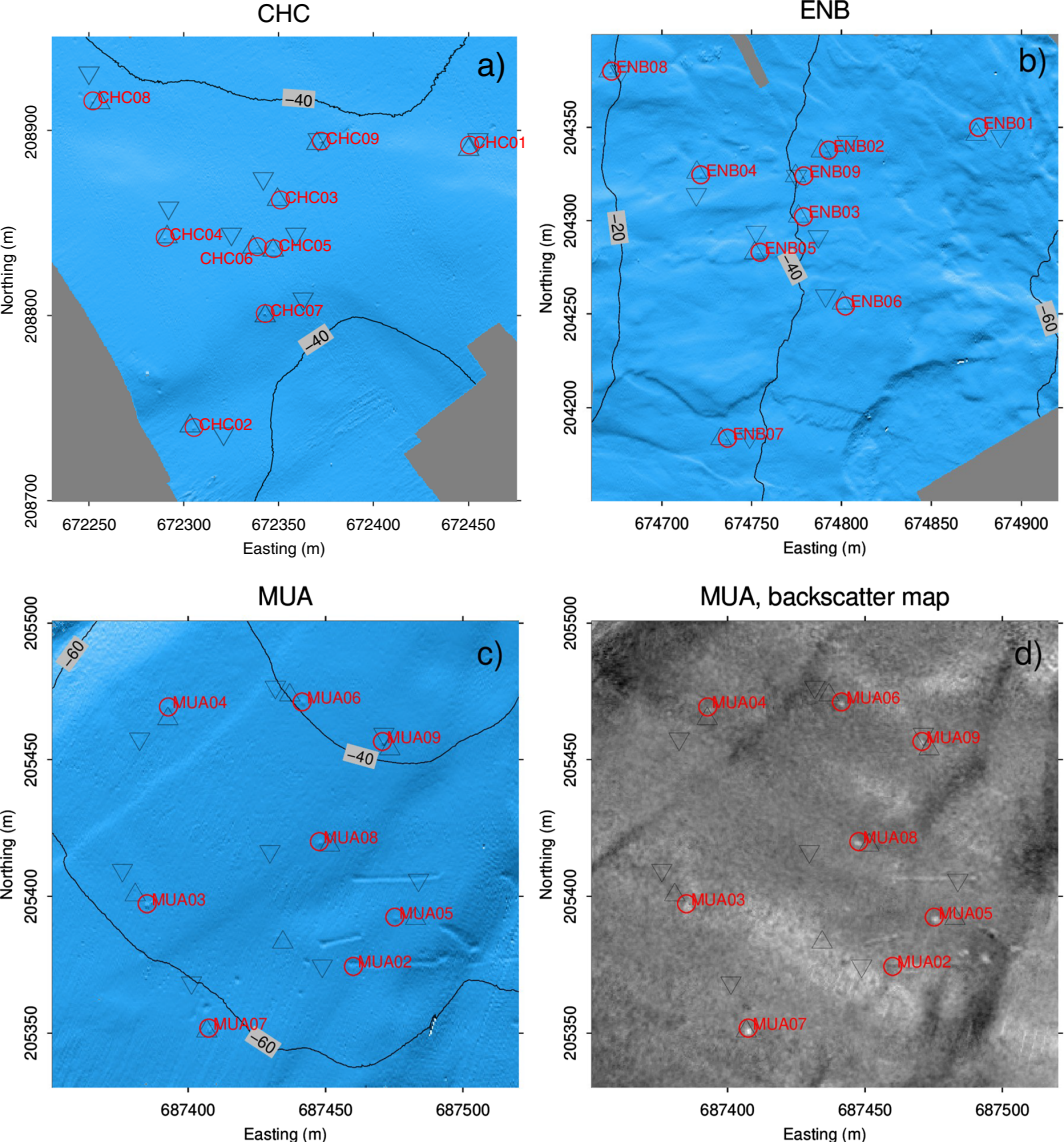
Multibeam Bathymetric map at: **a** Chrüztrichter array C (CHC); **b** Ennetbürgen array B (ENB); **c** Muota array A (MUA). The cell size is 0.5 m for OBS identification. Picked MB coordinates (circles), as well as the deployment (reverse triangles) and recovery coordinates (triangles) are indicated. **d** Backscatter map at MUA

In the presence of noise or other morphological objects visible on the MB data (blocks, artifacts), pinpointing the OBS on the lake floor may not be straighforward. To tackle this issue, we ensured that, during the OBS recovery, the OBS recovery coordinates were measured with the dGPS when the rope associated with the popup buoy was stretched vertically. Figure 2 uses different geometrical symbols to indicate the OBS positions at different phases of the operation, i.e., deployment (dGPS), post-deployment (MB), recovery (dGPS). Examples of positions are shown for the OBS arrays at Chrüztrichter, Ennetbürgen, and Muota. At Chrüztrichter, the lake floor geomorphology is smooth. The combination of the OBS positions at different phases of the operation allowed us to pick the unique OBS location on the lake floor. At Ennetbürgen, the lake floor has a rough surface geomorphology, but here again the combination of the OBS position at different phases allowed us to pick the unique OBS location on the lake floor. At Muota, strong scatterers are observed on the lake floor. In addition to the OBS position at different phases, the backscatter map shows the morphologic imprints of the OBS stations (e.g. Fig. 2d for MUA). This information is used to pinpoint the correct OBS location. The OBS stations are visible and are slightly offset of the planned deployment and recovery coordinates.

Once the OBS are identified on the bathymetric map, the MB coordinates of the OBS are considered as the most accurate, since the coordinates are neither affected by the post-deployment down-slope sliding of the OBS, nor biased by drifting of the OBS during deployment and recovery due to water currents. With this method, we achieve a location error in the range between 1.30 and 2.15 m. The lower error bound corresponds to the maximum length of the NAMMU on the ground (0.8 m) augmented by the MB horizontal grid resolution (0.5 m) and the upper error bound corresponds to the maximum length of the LOBSTER location on the ground (1.65 m) augmented by the MB horizontal resolution. Figure 2 presents the OBS locations for the arrays CHC, ENB, and MUA at Chrüztrichter, Ennetbürgen, and Muota, respectively. Further location examples for St. Niklausen A (NIA), B (NIB) and C (NIC), Chrüztrichter A (CHA) and B (CHB), Kehrsiten A (KEA) and B (KEB), Weggis A (WEA), B (WEB), C (WEC) and D (WED), Nase A (NAA), Ennetbürgen A (ENA), and Chindli A (CIA) are shown in  Figs. B.1, C.1, E.1, F.1, H.1, I.1, K.1, L.1, M.1, N.1, O.1, P.1, Q.1, R.1, of the supplementary information.

## Airgun Acquisition: Misorientation and Clock Error Correction

### Airgun Acquisition

Most OBS measurements are complemented by an extensive airgun survey prior to the OBS recovery from the lake floor. The purpose of the airgun survey is twofold: the estimation of the misorientation of the horizontal OBS components (Duennebier et al. 1987) and the estimation of the clock error of the OBS with respect to a selected station of the array. The operated airgun equipment is an electromechanical system with a 16.39 ml (1 $\hbox {in}^3$) air chamber operated at a pressure between 7 and 8 MPa. The airgun was lowered in the lake about 1 m below the water surface. It was trailed and set to release the compressed air every 18 s. This time between airgun shots was sufficiently long to ensure that the coordinates of the shooting points can be manually measured with the differential GPS and to prepare for the next shooting point. Each shot time was stored by a Centaur datalogger connected to the GPS and specifically prepared for this purpose.

**Fig. 3:**
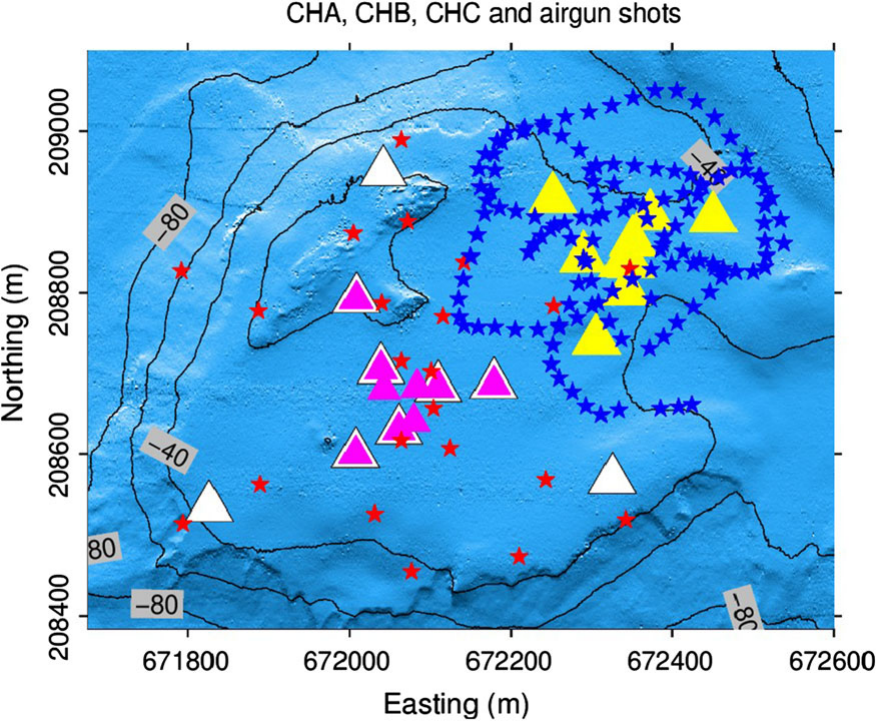
OBS locations and airgun shooting path at Chrüztrichter. OBS locations at CHA, CHB, and CHC are marked with white, magenta, and yellow triangles, respectively. CHA and CHB have 6 co-located stations. The red and blue stars indicate the airgun shooting path at CHB and CHC, respectively. The airgun path on top of CHB is a typical example of an airgun shot geometry with poor azimuthal coverage, especially for the outer stations. The airgun path on top of CHC is an example of good azimuthal coverage. The contour lines indicate the water depth. See Figs. C.2, D.1, E.2, F.2, G.1, I.2, J.1, K.2, N.2, O2, P.2, Q.2, R.2 in the supplementary information for more examples illustrating the airgun coverage

Figure 3 shows the OBS locations at CHA, CHB, and CHC with airgun shooting points on top of the arrays. The airgun configuration was sparser in the earlier campaign as shown for example by the airgun shooting path on top of CHA and CHB. As a consequence of the sparse airgun shot positions, the statistical interpretation of the mean OBS misorientation was difficult. The airgun shooting configuration was later optimized to ensure that we obtain a good azimuthal coverage around each OBS station in an offset sufficient to obtain a good signal-to-noise ratio on the OBS components. See Fig. 3 for an example of airgun shooting geometry at array CHC. Further examples of airgun shooting geometries can be found in Figs. C.2, D.1, E.2, F.2, G.1, I.2, J.1, K.2, N.2, O.2, P.2, Q.2, R.2 of the supplementary information.

With a dominant frequency of the airgun of about 1200 Hz and knowing that the recorder of the OBS operates at 250 Hz, it is clear that the first signal detected by the OBS is not the primary pulse, but the signal generated by the subsequent collapse of the air bubbles, including diffracted and reflected waves. In the particular case of reflected waves, a $180^{\circ }$ ambiguity may be observed in the OBS misorientation estimation.

### OBS Misorientation Estimation Using the Airgun Signal

**Fig. 4:**
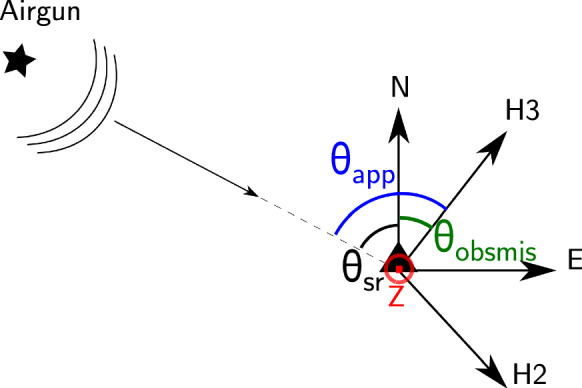
Schematic representation of the horizontal component misorientation angle on the lake floor. N and E indicate the geographic north and East, respectively. H2 and H3 are the horizontal components of the OBS with unknown orientations. $\theta _{app}$ is the apparent azimuth, $\theta _{sr}$ is the source - receiver azimuth, and $\theta _{obsmis}$ is the OBS misorientation angle

After the free-fall descent of the OBS in the lake, the horizontal components’ orientation is unknown. However, we know that the gimbal system ensures that the vertical component is always oriented towards the true vertical up. Figure 4 shows the schematic representation of the OBS horizontal components’ misorientation on the lake floor.

**Fig. 5:**
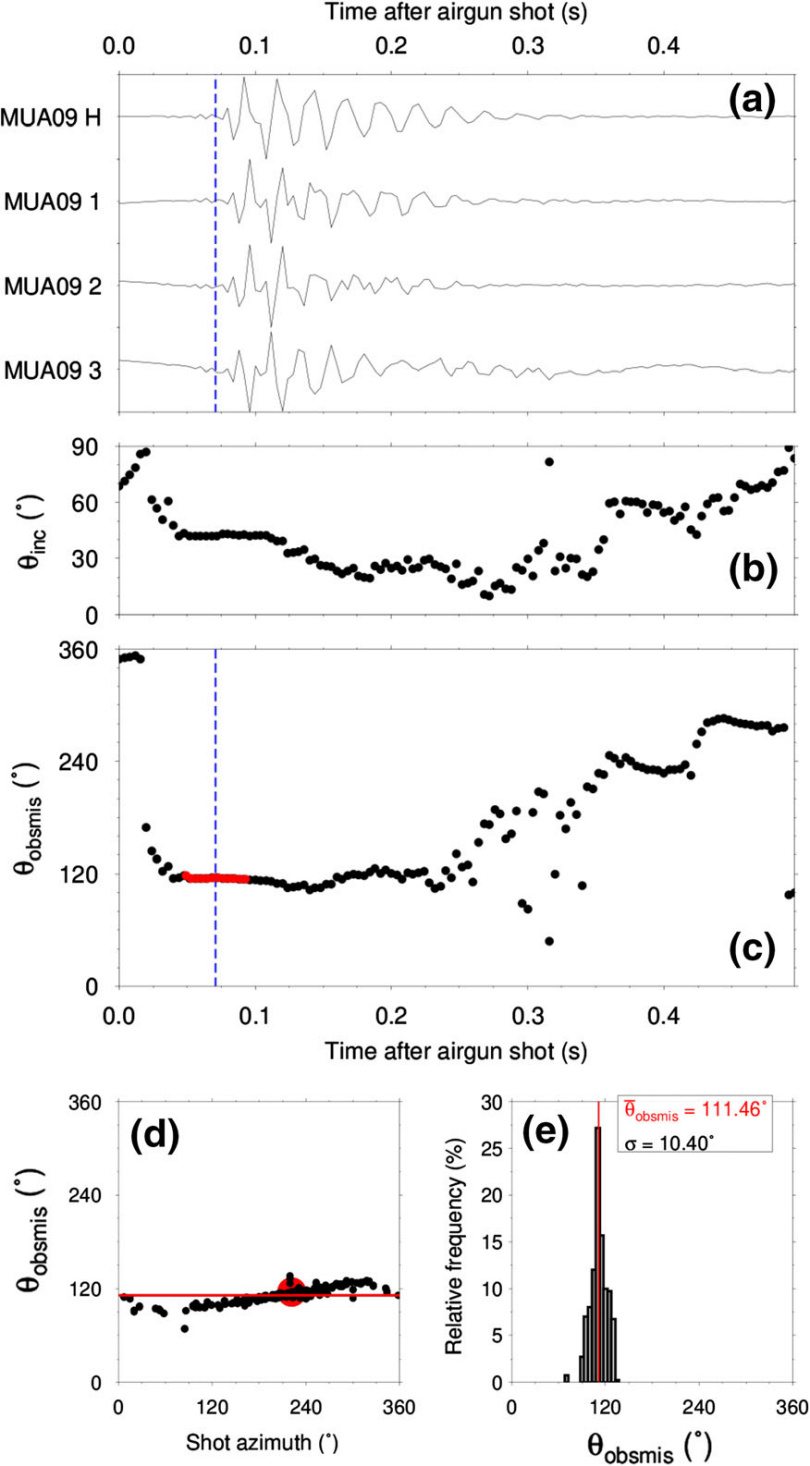
**a** Sample airgun shot recorded by the four components of the OBS station MUA09 at Muota. MUA09 H corresponds to the hydrophone component; MUA09 1, 2, and 3 correspond to the components Z, H2, and H3 as shown in Fig. 4, respectively. **b** Variations of $\theta _{inc}$ with time. **c** Variations of $\theta _{obsmis}$ with time. The dashed vertical line indicates the theoretical arrival time at 0.07 s. The source-receiver distance is 103 m and the average P-wave velocity in water is 1450 m/s. The red dots around the theoretical arrival time indicate the picked misorientation angles. d) Variations of $\theta _{obsmis}$ with respect to the airgun shot azimuth. e) Histogram of the $\theta _{obsmis}$ values with a mean value ${{\bar{\theta }}}_{obsmis}$ of $(111.46 \pm 10.40)^{\circ }$

We developed a semi-automatic procedure called obsmis (OBS MISorientation) that exploits the signal generated by the airgun to estimate the misorientation of the horizontal OBS components. We search for the rotation angle maximizing the signal energy on the radial component, parallel to the direction of propagation of the airgun impulse, and at the same time minimizing the signal energy on the transverse component (see also Duennebier et al. 1987). Obsmis estimates the polarization of the airgun signal according to Jurkevics (1988). We define data windows of 12 samples with 90% overlap. The number of samples was selected to potentially capture the first P-wave arrival while reducing the contamination from the reflected and diffracted waves, and the time window overlap parameter was set to ensure a good visual inspection of the obsmis values with time after the airgun shot. The recorded airgun shot signal is high-pass filtered with a butterworth filter of order 3, and corner frequency 5 Hz. For each defined data window, the covariance matrix is calculated as

1 $$\begin{aligned} S = \begin{pmatrix} \sum x^2(t) &{} \sum x(t) y(t) &{} \sum x(t) z(t) \\ \sum x(t) y(t) &{} \sum y^2(t) &{} \sum y(t) z(t) \\ \sum x(t) z(t) &{} \sum y(t) z(t) &{} \sum z^2(t) \end{pmatrix}, \end{aligned}$$

where *x*(*t*), *y*(*t*) and *z*(*t*) are the signals of the eastern, northern and vertical components, respectively. The three eigenvalues of *S* are linked with the three axes of the polarization ellipsoid. Therefore, the normalized eigenvectors $$\mathbf {v} = \begin{pmatrix}v_x \\ v_y \\ v_z \end{pmatrix}$$ and $-\mathbf {v}$ are both associated with the largest eigenvalue of *S* and indicate the direction of maximum polarization. The ambiguity between $\mathbf {v}$ and $-\mathbf {v}$ is solved based on the assumption of the airgun shot signal as a pure P-wave. The shot location at the water surface is always at higher elevation than the recording OBS at the lake bottom. Therefore, the $v_z$ component of the correct vector has to be positive and this vector is identified as the one pointing from the OBS to the airgun source. We can deduce the apparent azimuth $\theta _{app}$ and incident angle $\theta _{inc}$ as $\theta _{app} = \arctan \left( \dfrac{v_x}{v_y} \right)$, if $v_y > 0$ or $\theta _{app} = \arctan \left( \dfrac{v_x}{v_y} \right) + 180^{\circ }$, if $v_y < 0$, and $\theta _{inc}=\arccos (v_z)$, respectively. Knowing the locations of the shot point and the OBS, we easily determine the source-receiver azimuth $\theta _{sr}$ of the signal for each shot and calculate the misorientation angle of the OBS from each moving window as $\theta _{obsmis} = \theta _{app} - \theta _{sr}$. It is then straightforward to manually pick the misorientation value from the moving time window values. Figure 5 shows a sample airgun signal recorded at the OBS station MUA09 of array A at Muota and presents the azimuthal coverage and the estimated misorientation value ($\theta _{obsmis}$) at each azimuth, as well as the distribution of $\theta _{obsmis}$. The estimated $\theta _{inc}$ and $\theta _{obsmis}$ for each short time window are displayed. The incidence angle serves as quality measure to pick the appropriate $\theta _{obsmis}$ values, e.g., knowing the expected incidence angle, we can better isolate the part of the recorded signal which is related to the first arrival of the airgun signal, although this is not always trivial. The theoretical arrival time is marked by the vertical dashed blue line (Fig. 5a and c) and the samples which contributed to the statistical mean of $\theta _{obsmis}$ for this shot are marked as red dots (Fig. 5c, d). The picking procedure is repeated for all shots and the variations of the $\theta _{obsmis}$ values with respect to the shot azimuth as well as the relative frequency of occurrence are given in Fig. 5d, e. Using the picked $\theta _{obsmis}$ values from each shot, we estimate the mean orientation ${{\bar{\theta }}}_{obsmis}$ between 0 and 360$^{\circ }$ and the corresponding standard deviation $\sigma$ using Equations (2) and (3) (see e.g. p.33, Fisher 1993).

2 $$\begin{aligned} {{\bar{\theta }}}_{obsmis}= & {} {\left\{ \begin{array}{ll} \arctan \left( \frac{X}{Y} \right) &{} \quad \text {if }Y > 0 \\ \arctan \left( \frac{X}{Y} \right) + 180^{\circ } &{} \quad \text {if }Y < 0, \end{array}\right. } \end{aligned}$$

3 $$\begin{aligned} \sigma= & {} \sqrt{(-2ln(R))}, \end{aligned}$$

where $X=\sum \nolimits _{i=1}^N \sin (\theta _{obsmis,i})$, $Y=\sum \nolimits _{i=1}^N \cos (\theta _{obsmis,i})$, and $R= \dfrac{1}{N} \sqrt{X^2 + Y^2}$. *i* indicates the airgun shots and *N* is the total number of picked airgun shots.

**Fig. 6:**
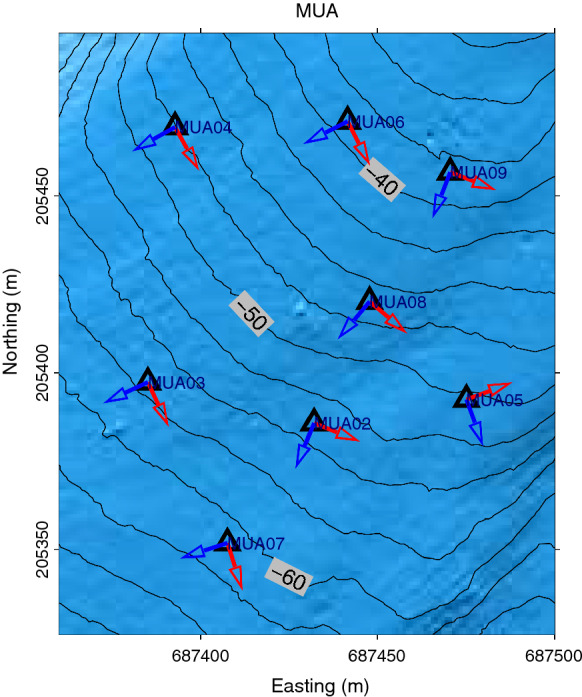
Estimated sensor orientations for array MUA at Muota. The blue and red arrows indicate the orientations of the H2 and H3 components, respectively. The OBS uses a right-hand system with the vertical component pointing upward as illustrated in Fig. 4

The component misorientations for MUA are given in Fig. 6. Detailed results presenting the variations $\theta _{obsmis}$ with respect to $\theta _{sr}$ and the corresponding histograms (similar to Fig. 5d and e) Fig. 6 for MUA as well as at CHB, CHC, CIA, ENA, ENB, KEB, NAA, NIC, WEB, WEC, and WED at each specific station are given in Figs.  C.3, D.2., E.3, F.3, G.2, I.3, J.2, K.3, N.3, P.3, Q.3, R.3 of the supplementary information, respectively. The summary plot of the component misorientations, similar to Fig. 6, is given in Figs. C.4, D.3, E.4, F.4, G.3, I.4, K.4, N.4, P.4, Q.4, R.4. Usually, the standard deviation of the estimated misorientation angle is about $10^{\circ }$, but can also be larger. These large standard deviation values can be understood as caused by the slight azimuthal dependence on the slope gradient of the lake floor morphology or error in OBS location and airgun shot positioning.

Although the $180^{\circ }$ ambiguity is solved by using the upward-pointing eigenvector, an important observation is that it is still observed for some sites. This may be caused by the complex nature of the analyzed time window signal, including for example reflections or refractions on interfaces at the lake bottom. In these cases, the ambiguity is solved by using the bathymetry map and by assuming that the direction of maximum down-going slope provides a constraint for the X (or 2) direction. This assumption is supported by the results obtained at MUA where no polarity flipping was observed (see Fig. 5d, e and Figure J.2 of the supplementary information). We therefore cross-checked the orientation obtained from the airgun shooting with the expected orientation from the bathymetry. The orientation of the horizontal components might however be affected by small objects or small-scale topographic features not resolved by the bathymetry.

### Clock Error Correction

Starting from the clock error value measured at OBS recovery, a clock correction can be performed by assuming a linear clock-drift behavior (Hannemann et al. 2013; Hable et al. 2018). However, the clock can also drift nonlinearly (Gouédard et al. 2014). Here, we assumed that the absolute clock error measured at recovery when the recorder and GPS are synchronized is representative for the OBS, independently of the linear or non-linear clock-drift behavior. The assumption holds for short-term measurements, and changes within the last two hours prior to OBS recovery can be neglected. This assumption allows us to shift the recordings with the respective clock error.

**Fig. 7:**
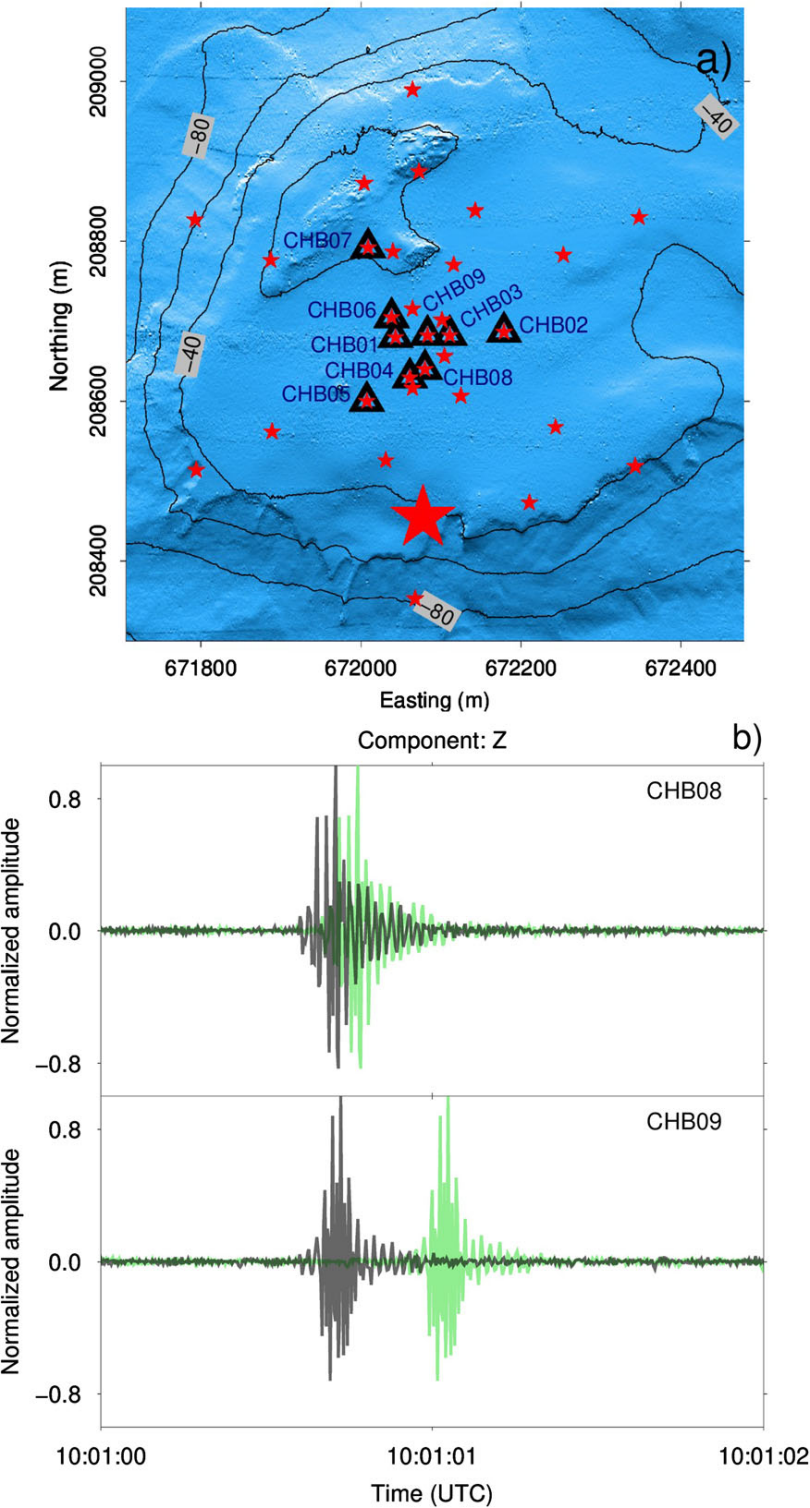
**a** The black triangles indicate the final array configuration on the lake floor for CHB. The red stars indicate the airgun shot locations. The large star indicates the airgun shot position for which we present the corrected and uncorrected signals. **b** The uncorrected traces are shown in light green and the clock-error corrected traces are shown in gray for the reference station CHB08 and the station CHB09. Each trace is normalized to its maximum amplitude. Measured and airgun-estimated clock errors are given in Table 3. The measured clock error at recovery was used for the correction (gray curves)

Alternatively to the clock error measured at the recovery of the OBS, we also used the airgun signal to estimate the relative clock error. The relevance of the airgun to estimate the clock error is two-fold: the clock error at some stations is non-linear and some arrays have co-located OBS stations with other arrays. In this case, the clock error of the stations that remain on the lake floor cannot be measured until they are recovered. A linear optimization process is able to find the clock error that minimizes the travel time difference with respect to a reference station. An example that uses airgun shots for clock correction is shown in Fig. 7. It shows the example of array B (CHB) at Chrüztrichter. The choice for CHB is motivated by the fact that some OBS stations of the array exhibited large clock errors. CHB was obtained from CHA by changing the location of three OBS stations. CHA operated for 55 days since May 8th, 2018. CHB was deployed for 2 days, starting from July 3rd, 2018 08:10:41 to July 5rd, 2018 07:36:49 UTC. Six co-located OBS stations at CHB were recording from May 8th, 2018 09:46:12 UTC. The airgun shot experiment took place on July 4th, 2018. In Fig. 7, the green signals show the raw traces and the gray curves the ones after time correction. All stations are time-corrected with respect to the OBS station CHB08 that had the lowest clock error at recovery. Table 3 gives the measured clock error at the OBS recovery ($\Delta T_{GPS}$), and the clock error estimated from the airgun shots ($\Delta T_{airgun}$). The clock error ranges from $-3.287$ to $-1.8685$ s for the 2-month recording period. These variations are very large, as we expected a variation in the order of 0.1 s for a clock timing accuracy of 0.02 ppm.

**Table 3:** Clock error estimates using GPS and airgun measurements for array B (CHB) at Chrüztrichter

Station	$\Delta T_{GPS}$ (s)	$\Delta T_{airgun} \pm std$ (s)
CHB01	$-0.7806$	$-0.7816 \pm 0.0205$
CHB02	$-1.8702$	$-1.8685 \pm 0.0394$
CHB03	$-2.3265$	$-2.3206 \pm 0.0252$
CHB04	$-3.2870$	$-3.2591 \pm 0.0140$
CHB05	$-2.5900$	$-2.5858 \pm 0.0276$
CHB06	$-2.7665$	$-2.7687 \pm 0.0259$
CHB07	$-2.5701$	$-2.5606 \pm 0.0301$
CHB08	$-0.0668$	$-0.0668 \pm 0$ (Ref. station)
CHB09	$-0.3243$	$-0.3095 \pm 0.0345$

After changing the recorders of the OBS in September 2018, the clock error of the measurements significantly improved. Also, the error was of the same order as the expected airgun-based time shift estimates. For this reason, for the array measurements with changed recorders, the time correction was performed by using the clock error at recovery.

## Data Processing: Extraction of Scholte and Love Waves Phase-Velocity Dispersion Curves

Two array processing techniques are used to estimate the phase velocity dispersion curves with the array data measured at Muota. The first approach is the three-component High Resolution Frequency-Wavenumber technique for both Scholte and Love waves DC extraction (Poggi and Fäh 2010). The second approach is the Interferometric Multichannel Analysis of Surface Waves for Scholte waves DC extraction (Lontsi et al. 2016). The application of the cross-correlation techniques to the noise wavefield allows to reconstruct the Green’s function between two receivers. The causal and acausal correlations Green’s functions are symmetric when the noise wavefield is diffuse, or otherwise asymmetric when this principle is not fulfilled. In practice, both the causal and acausal part of the correlations Green’s functions are stacked for DC estimations.

### High Resolution Frequency-Wavenumber Approach

For the data analysis, we selected a 2-h time window of the recorded data preceding the OBS recovery. In an initial processing, the phase velocity dispersion curve is sought on raw data where the locations of the OBS stations are obtained using dGPS at recovery, and no orientation and clock errors are corrected. Second, we preprocess the data following our workflow presented above. We apply a multi-step correction procedure: first, we replace the dGPS coordinates with the multibeam (MB) coordinates; then, we make a clock correction by shifting the traces by the clock error value and assuming a constant clock error for each station during the analyzed time window; the last step is the misorientation correction. The ${\bar{\theta }}_{obsmis}$ estimates for OBS stations at Muota are given in Table 4 and Fig. 6. Table 4 further gives the clock errors measured at recovery ($\Delta T_{GPS}$), the coordinates of the OBS stations measured with the dGPS and with the multibeam bathymetry, and also the variation in terms of distance between the two measured positions. Here we use the clock error measured at recovery because their values are in the same order as the error of the clock error estimates obtained with the airgun procedure.

**Table 4:** Preprocessing parameters for the array MUA. The parameters are the OBS coordinates (CH_X, CH_Y, CH_Z) at recovery using the dGPS and multibeam, the variation between the dGPS and multibeam ($\Delta d$), the misorientation angles (${\bar{\theta }}_{obsmis}$) and the clock error. The coordinates are given in the Swiss Coordinate System LV03

OBS name	Coordinates using dGPS (m)			Coordinates using MB (m)			$\Delta d$ (m)	${\bar{\theta }}_{obsmis}$	$\Delta T_{GPS}$
	CH_X	CH_Y	CH_Z	CH_X	CH_Y	CH_Z		($^{\circ }$)	(s)
MUA02	687,434.48	205,383.32	378.9	687,432.07	205,385.76	378.9	3.43	$113 \pm 18$	0.063
MUA03	687,380.73	205,400.66	375.8	687,385.11	205,397.33	376.2	5.52	$147 \pm 25$	0.017
MUA04	687,392.66	205,465.10	384.7	687,392.78	205,469.42	385.4	4.38	$150 \pm 10$	0.044
MUA05	687,482.65	205,391.89	382.8	687,475.06	205,392.42	382.4	7.62	$72 \pm 23$	0.012
MUA06	687,436.88	205,473.76	393.8	687,441.47	205,471.09	393.9	5.31	$148 \pm 25$	$-0.209$
MUA07	687,406.79	205,350.86	371.7	687,407.53	205,351.75	371.9	1.19	$163 \pm 27$	0.052
MUA08	687,451.51	205,418.77	386.6	687,447.69	205,420.09	386.8	4.04	$127 \pm 19$	$-0.009$
MUA09	687,473.39	205,454.02	394.1	687,470.57	205,456.66	394.6	3.90	$111 \pm 10$	0.040

**Fig. 8:**
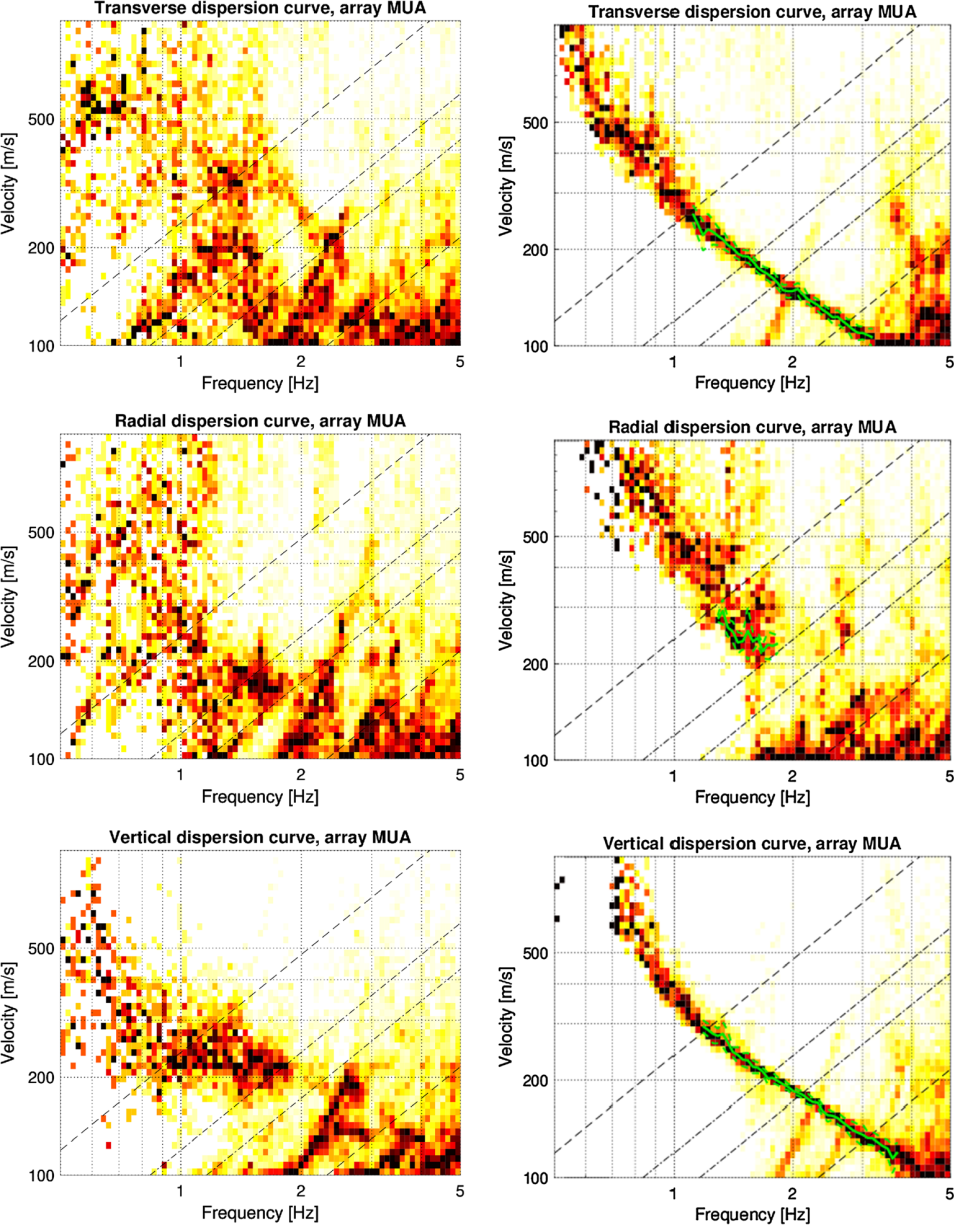
Phase-velocity dispersion curves at Muota (array MUA). Left panels: results without preprocessing steps. Right panels: results with consideration of the three preprocessing steps (position, orientation, clock error). The dashed and dash-dotted black lines are the theoretical array resolution limits. The green lines indicate the phase-velocity dispersion curves that were picked

To investigate the DC from the 3C HRFK technique for both the raw and preprocessed data, the two hours of continuous noise recording for each of the three components is splitted into short time windows with length corresponding to $50\cdot \hbox {T}$, where $T=\dfrac{1}{f_c}$ and $f_c$ is the central frequency. For each short time window, the beampower is estimated for each central frequency between 0.5 and 5 Hz and for velocities between 100 and 1000 m/s. A histogram visualization as density plot of the beampower results in a frequency velocity diagram, allows us to pick, within the resolution limits, the DC that are best representative of the measurements. Figure 8 presents an example of phase-velocity dispersion curves extracted from the array at Muota (MUA) before (raw data) and after location, time and misorientation correction of the data (preprocessed data).

As expected, there is no clear sign and shape of the dispersion curves in case of uncorrected data, even for the vertical component, where only the time and location corrections are important. For the corrected recordings, we observe very clear and well-resolved dispersion curves on the transverse and vertical components, which provide information about the velocities of Love and Scholte waves, respectively. We also observe an improved result on the radial component that is relevant for Scholte waves.

### Interferometric Multichannel Analysis of Surface Waves

**Fig. 9:**
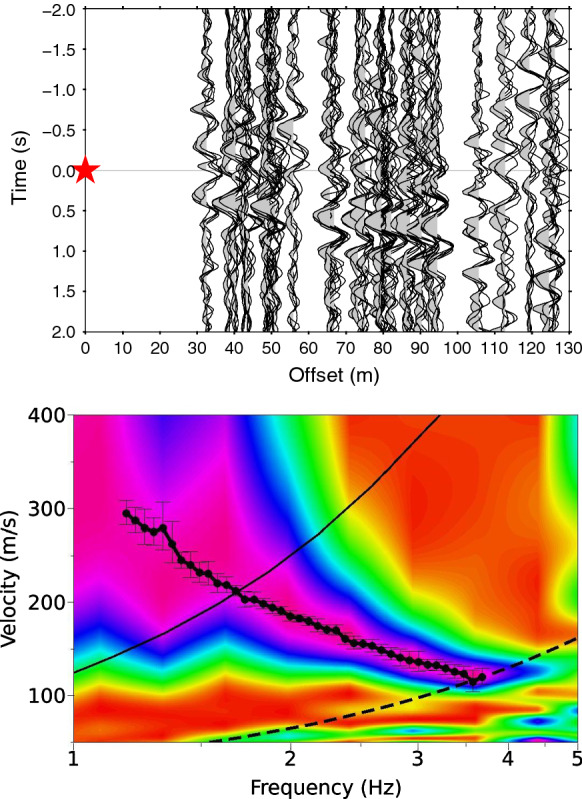
Top: Correlograms from all receiver pair combinations (See array setup for Muota in Fig. 3). The red star represents the virtual source. Bottom: Scholte wave phase-velocity dispersion curve map from the virtual array set-up. A phase-velocity dispersion branch of Scholte waves is observed within the resolution limits defined by the continuous and dashed black curves. The continuous curve is linked to the maximum analyzed inter-station distance and the dashed curve is linked to the offset between the virtual source and the first receiver. The dotted curve with error bars corresponds to the picked Scholte wave DC from HRFK (Fig. 8)

The advantages of active shot experiments with known source location and passive microtremor recordings are combined by using, in a first step, the interferometric principle (Snieder 2004; Curtis et al. 2006; Schuster 2009; Wapenaar et al. 2010) to estimate the correlation functions for the vertical components of the distinct receiver pairs. Here, we analyze the vertical component of the recording and perform the analysis on corrected data. The ambient vibration recordings are first splitted into 30 min long data segments. For each data segment, the cross-correlation is performed for each station pair on 10 s windows with 50% overlap. The cross-correlation results are saved for 2 s for the positive (causal) and negative (acausal) lag time. The final cross-correlogram for each station pair is obtained from the stack of the cross-correlation results. Assuming the equivalence between the time-derivative of the inter-station cross-correlation function and the Green’s function, the correlograms are re-ordered according to the respective inter-station distance to build a virtual active experiment setup (Fig. 9). We observe, especially for the positive lag time, a clear Scholte wave propagation.

We then apply, in a second step, the frequency-wavenumber technique to the virtual active source data also known as Interferometric Multichannel Analysis of Surface Waves (IMASW; Lontsi et al. 2016) to extract the phase velocity dispersion curve of Scholte waves. For the DC analysis, the causal and acausal part are stacked. The beampower is estimated for frequencies ranging from 0.4 to 8 Hz, and for velocities ranging from 50 to 600 m/s. Figure 9 (bottom) shows the phase-velocity dispersion. The dispersion characteristic of the Scholte waves is clearly identified within the resolution limits that are defined by the virtual source-to-first receiver offset and the maximum interstation distance. The DC for Scholte waves obtained using the HRFK is overlaid for comparison and both show a good agreement within the resolution limits.

## Discussion

**Fig. 10:**
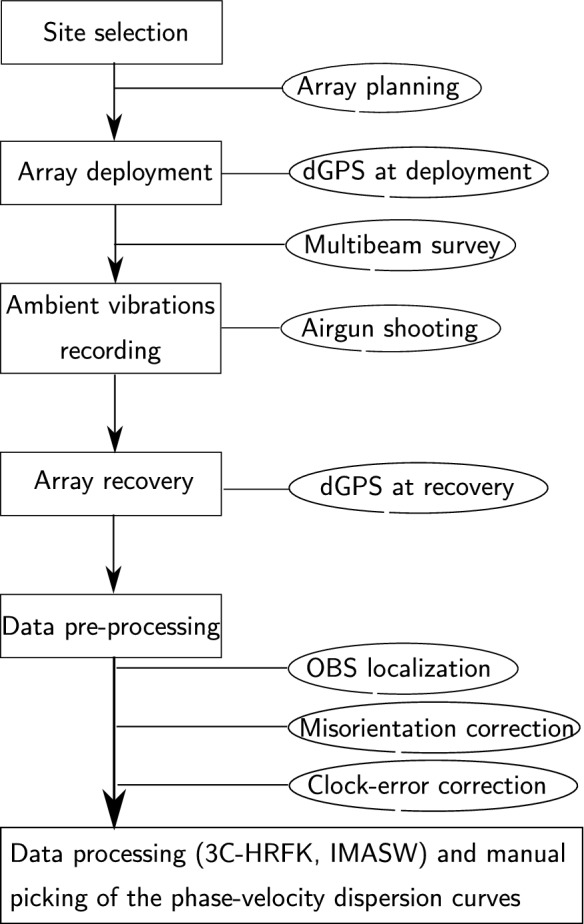
Schematic representation of the workflow for phase-velocity dispersion curve estimation using OBS data

A successfull processing the OBS data for the estimation of the phase velocity dispersion curve requires additional steps in the data preparation phase that are usually not part of the processing procedure of onshore data. Our workflow is summarized in Fig. 10.

The first step is the localization of the OBS on the lake floor. We have shown that, by using a combination of the multibeam bathymetry, the backscatter map, and the differential GPS data at deployment and recovery, we can uniquely identify the position of the OBS on the lake floor with an uncertainty of 1.3 m for the NAMMU and 2.15 m for the LOBSTER. These uncertainty values correspond to the maximum length of the OBS on the lake floor added to the multibeam grid resolution of 0.5 m. The location procedure is optimized for shallow water environments and is technically limited in deep water environments by the maximum rope length that the bucket associated with the OBS can accommodate. An additional factor affecting the measurement of the OBS position at recovery is the presence of water currents, or the drift of the boat. It can therefore be difficult to have the rope in a vertical position. Nevertheless, dGPS measurements are used to aid in the interpretation of the multibeam measurement. As a consequence of the uncertainties on the position, seismic waves with wavelengths below 4.3 m cannot be resolved.

We addressed the challenges related to the sensor orientation by using airgun data. In the absence of airgun signals, methods based on earthquakes (Stachnik et al. 2012; Doran and Laske 2017) or cross-correlation Green’s functions (Zha et al. 2013) can be used. While earthquake-based polarization analysis may suffer from limited azimuthal coverage especially at the areas of low-to-moderate seismicity or when the seismicity is azimuth band-limited. Airgun-based polarization analysis has shown to be very efficient in this study.

We retrieved the misorientation of the sensor with a $180^{\circ }$ ambiguity at many sites. The reason why we still observe the ambiguity lies in the analyzed data. The sampling rate is 250 Hz and the dominant frequency of the airgun signal is about 1200 Hz. With this limitation, the analyzed window does not contain pure direct P-waves. As a consequence, the obsmis estimates may be ambiguous. MUA is a very good example of a ${{\bar{\theta }}}_{obsmis}$ representation between 0 and $360^{\circ }$ without ambiguity. The location procedure, the airgun measurement, obsmis at the remaining arrays, sorted site-by-site, are shown in Figs. B1 to R.4 of the supplementary information. With the sampling frequency limitation mentioned above, it is also expected that MUA shows the ambiguity for some shot azimuths, but this is not the case. This is probably related to the maximum shot-receiver distance that was analyzed and indicates that a criteria may be set to define a critical shot-receiver distance to minimize the effects of diffracted and reflected waves. This scenario was not investigated. Instead, we took the advantage of the existing bathymetry data and the sensor orientation results obtained at MUA to address the ambiguity issue. As most of the stations were on slopes, addressing the ambiguity was relatively straightforward. Applying the preprocessing above, clear Scholte and Love waves phase-velocity dispersion curve branches were retrieved using two array methods, the 3C-HRFK and the IMASW. The comparison of the picked phase-velocity dispersion curves for the Scholte waves shows that within the error ranges and the resolution limits, the DC from 3C-HRFK and IMASW are comparable. For MUA, we observe that the Love waves are slower than the Scholte waves. In order to compare our observations with the results from Nolet and Dorman (1996) who find that Love waves are faster than Scholte waves, we first defined a simple two-layer over halfspace with a water on top (Table S.1 of the supplementary information) and calculate the Scholte and Love waves phase velocity between 0.2 Hz and 20 Hz for the fundamental mode. Modeling results are presented in Figure S.1 of the supplementary information. The results show that for the low frequency signals, the Love wave is indeed faster than the Scholte waves as observed for example by Nolet and Dorman (1996) on large aperture OBS array. However, this is not true for all frequency bands (Figure S.1). Second, we compare the Love and Scholte wave phase-velocity dispersion curves at CIA, ENA, and ENA (Figure T.1 in the supplementary information). The observations corroborate with modeling results, but the Scholte wave remains faster in most frequency bands. Further applications of the current workflow for phase-velocity dispersion curve estimation and their inversion for the subsurface structure at multiple OBS array sites at Lake Lucerne are given in Shynkarenko et al. (2021).

## Conclusion

As part of the initiative to assess the causes, control and mechanisms of mass-movement triggered tsunamis in lakes, we presented the workflow used to efficiently estimate the phase-velocity dispersion curves of Scholte and Love waves from small-aperture OBS ambient-vibration array data. The workflow involves three main steps. In the first step, a multibeam bathymetric (MB) survey is performed on top of each array to locate, with a precision up to the size of the OBS plus the MB grid resolution, the OBS locations on the lake floor. In the second and third steps, the airgun data are used to estimate the OBS misorientation and to correct the clock error. For arrays that present an ambiguity, each station was regarded individually especially when ${{\bar{\theta }}}_{obsmis}$ does not correspond to the mean of any predominant distribution. By applying all steps to the recorded data, we successfully obtained a clear phase-velocity dispersion curve for both Scholte and Love waves using two different array processing techniques.

## Supplementary Information

Below is the link to the electronic supplementary material.

Supplementary file1 (PDF 10269 KB)

## Data Availability

The OBS data are archived at ETHZ. Any data request should be addressed to the authors.
