# Supplementary material for: A Robust Workflow for Acquiring and Preprocessing Ambient Vibration Data from Small Aperture Ocean Bottom Seismometer Arrays to Extract Scholte and Love Waves Phase-Velocity Dispersion Curves
Source: Pure Appl Geophys. 2021 Dec 14;179(1):105–23. doi: 10.1007/s00024-021-02923-8 (PMC8752553; doi:10.1007/s00024-021-02923-8)
Supplement: Supplementary file 1 — Supplementary file1 (PDF 10269 KB) [file 24_2021_2923_MOESM1_ESM.pdf]

Supplementary information to "A robust workflow for acquiring and preprocessing ambient vibrations data from small aperture Ocean Bottom Seismometer arrays to extract Scholte and Love waves phase-velocity dispersion curves"

Agostiny Marrios Lontsi[1], Anastasiia Shynkarenko[1], Katrina Kremer[1,2], Manuel Hobiger[1,3], Paolo Bergamo[1], Stefano C. Fabbri[2], Flavio S. Anselmetti[2], Donat Fäh[1]

[1] *Swiss Seismological Service, Swiss Federal Institute of Technology (ETH), Sonneggstrasse 5, 8092 Zurich, Switzerland*
agostiny.lontsi@sed.ethz.ch, a.shynkarenko@sed.ethz.ch, katrina.kremer@sed.ethz.ch, paolo.bergamo@sed.ethz.ch, donat.faeh@sed.ethz.ch
[2] *Institute of Geological Sciences and Oeschger Centre for Climate Change Research, University of Bern, Baltzerstrasse 1+3, 3012 Bern, Switzerland*
stefano.fabbri@geo.unibe.ch, flavio.anselmetti@geo.unibe.ch
[3] *Federal Institute for Geosciences and Natural Resources (BGR), Stilleweg 2, 30655 Hanover, Germany*
manuel.hobiger@bgr.de

Summary

The current online material contains the following issues that support the main article:

- an annotated image of the OBS types used during the measurements in lake. This part is complementary to the subsection 2.2 "Instrumentation" in the main article.
- located OBS stations on top of the multibeam bathymetry map. This part is complementary to subsection 3.2 "OBS location procedure".
- airgun shots on top of the arrays, where available. The section is complementary to the subsection 4.1 "Airgun acquisition" in the main article.
- misorientation estimates at each OBS of arrays with airgun measurements, indicating the orientation of the horizontal components on top of the bathymetry map. This section is complementary to the subsection 4.2 "OBS misorientation estimation using the airgun signal" in the main article.

The preprocessing steps listed in the last three points above are presented site-by-site or some points are referenced to the main article where it applies.

A. OBS types

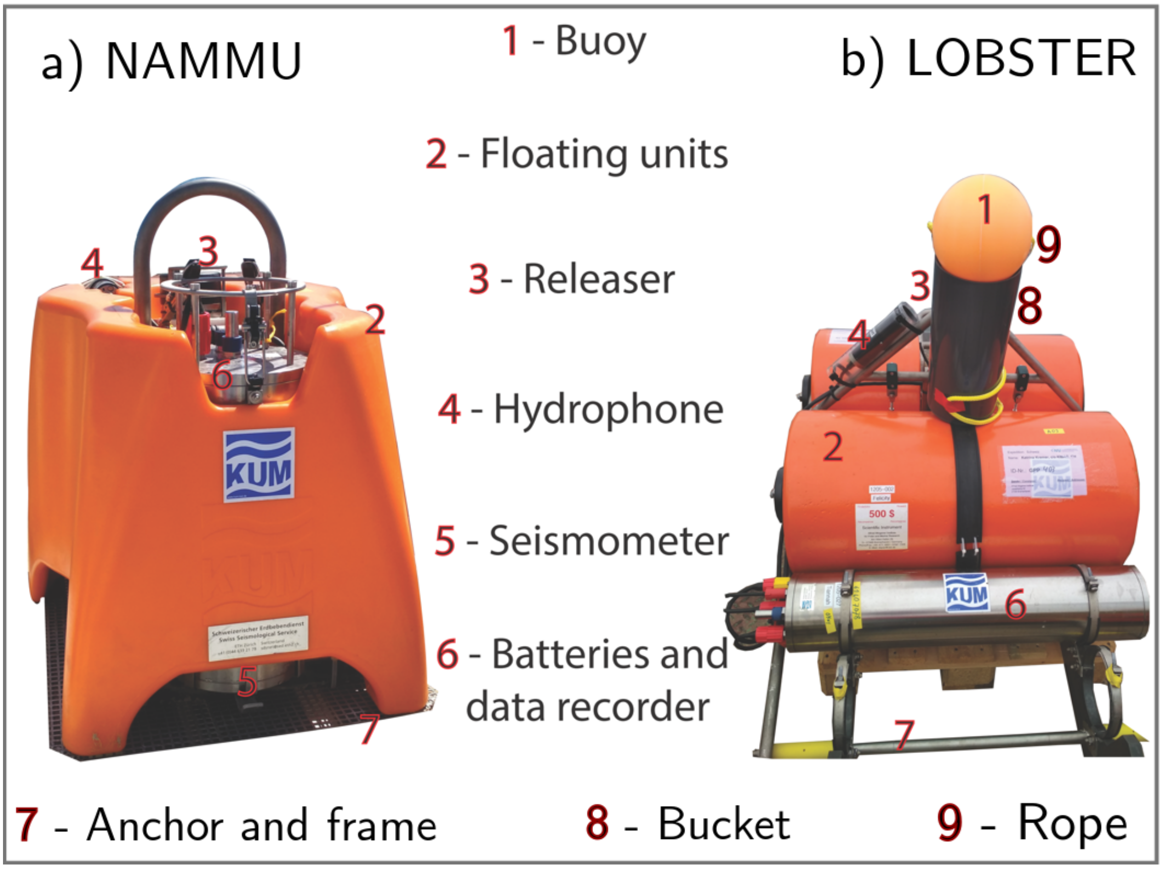

Figure A.1: OBS stations used in Lake Lucerne. a) NAMMU, b) LOBSTER. Both OBS types are equipped with popup buoy, bucket, rope, syntactic foam, releaser, hydrophone, seismometer, batteries, datalogger, anchor and frame. The annotation shows each component on both the NAMMU and LOBSTER. The NAMMU is half the LOBSTER in surface area on the ground. Table 1 in the main article gives an extended comparison of the main physical parts between the two devices.

B. Chrüztrichter: Array CHA

B.1. OBS locations

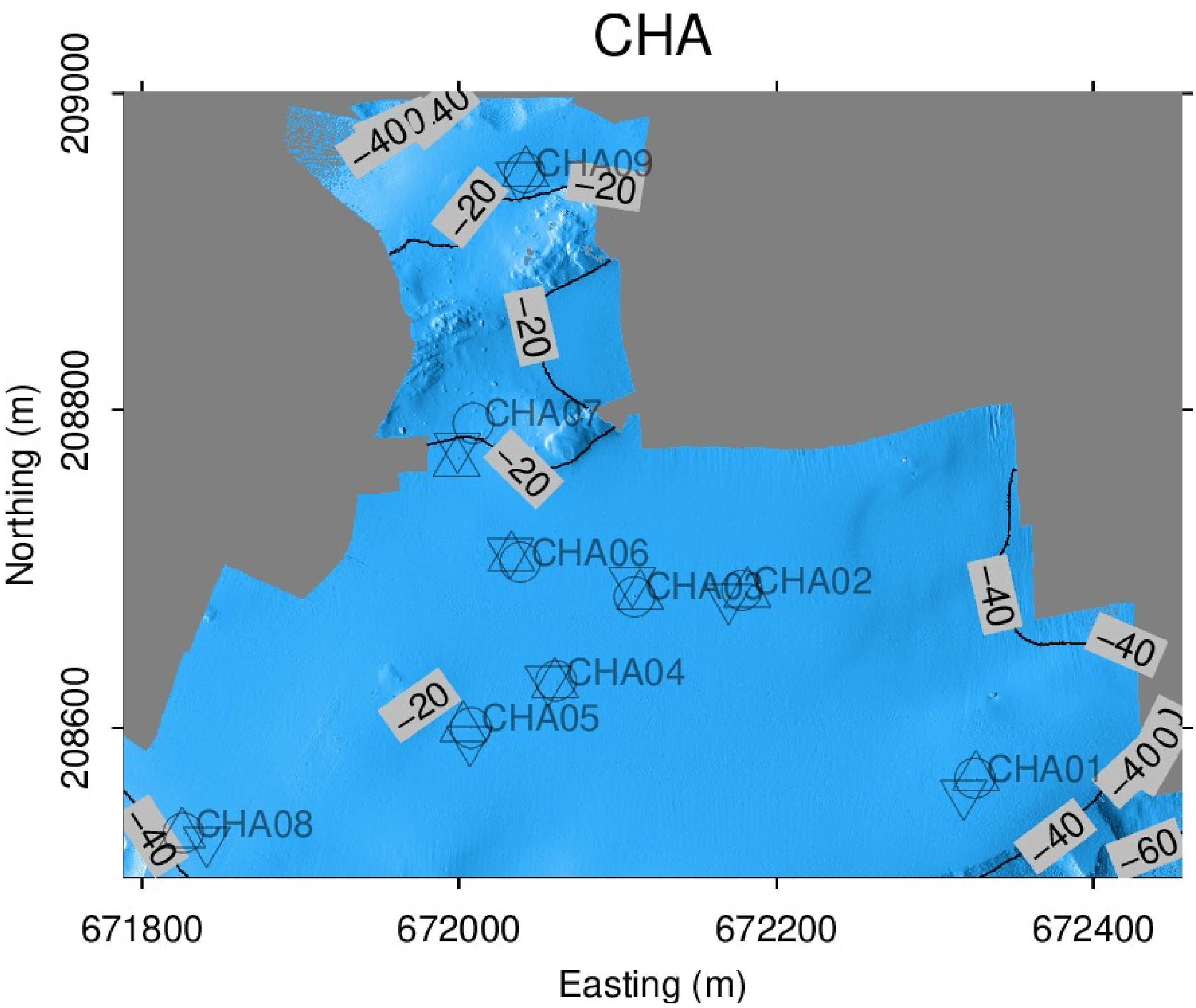

Figure B.1: OBS localization at CHA. The reverse triangle indicates the OBS position at deployment using the differential GPS (dGPS); the triangle indicate the OBS position at recovery using the dGPS; and the circle indicates the OBS position from multibeam. The contour lines give the water depth.

B.2. Airgun measurements

No airgin data available for array CHA.

B.3. OBS misorientation

No obsmis value available for array CHA.

B.4. OBS misorientation with base bathymetry

No obsmis value available for array CHA.

C. Chrüztrichter: Array CHB

C.1. OBS locations

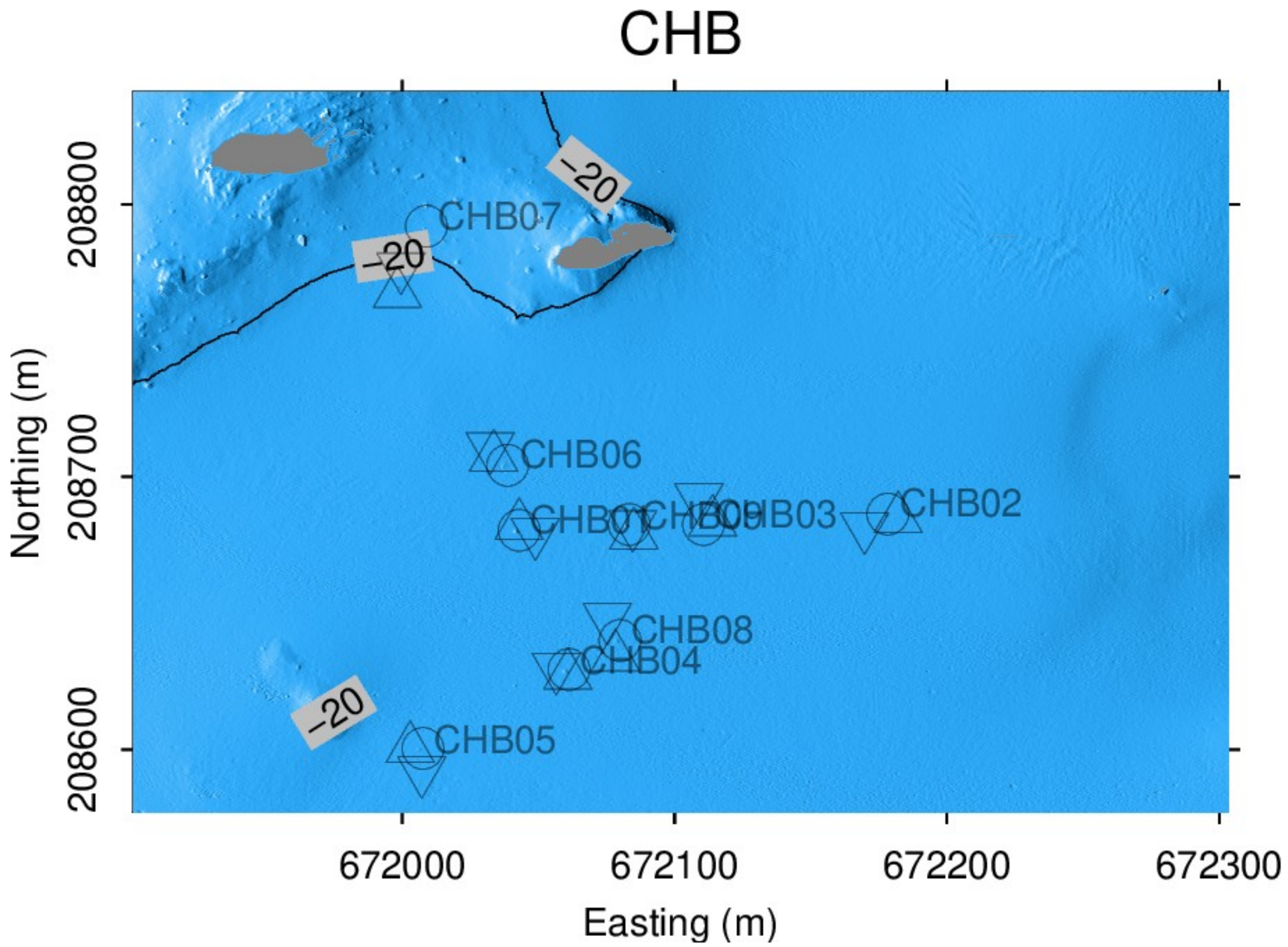

Figure C.1: OBS localization at CHB. The reverse triangle indicates the OBS position at deployment using the differential GPS (dGPS); the triangle indicate the OBS position at recovery using the dGPS; and the circle indicates the OBS position from multibeam. The contour lines give the water depth. Array CHB differs from array CHA by the position of the stations CHA01, CHA08, CHA09, and CHB01, CHB08, CHB09, respectively.

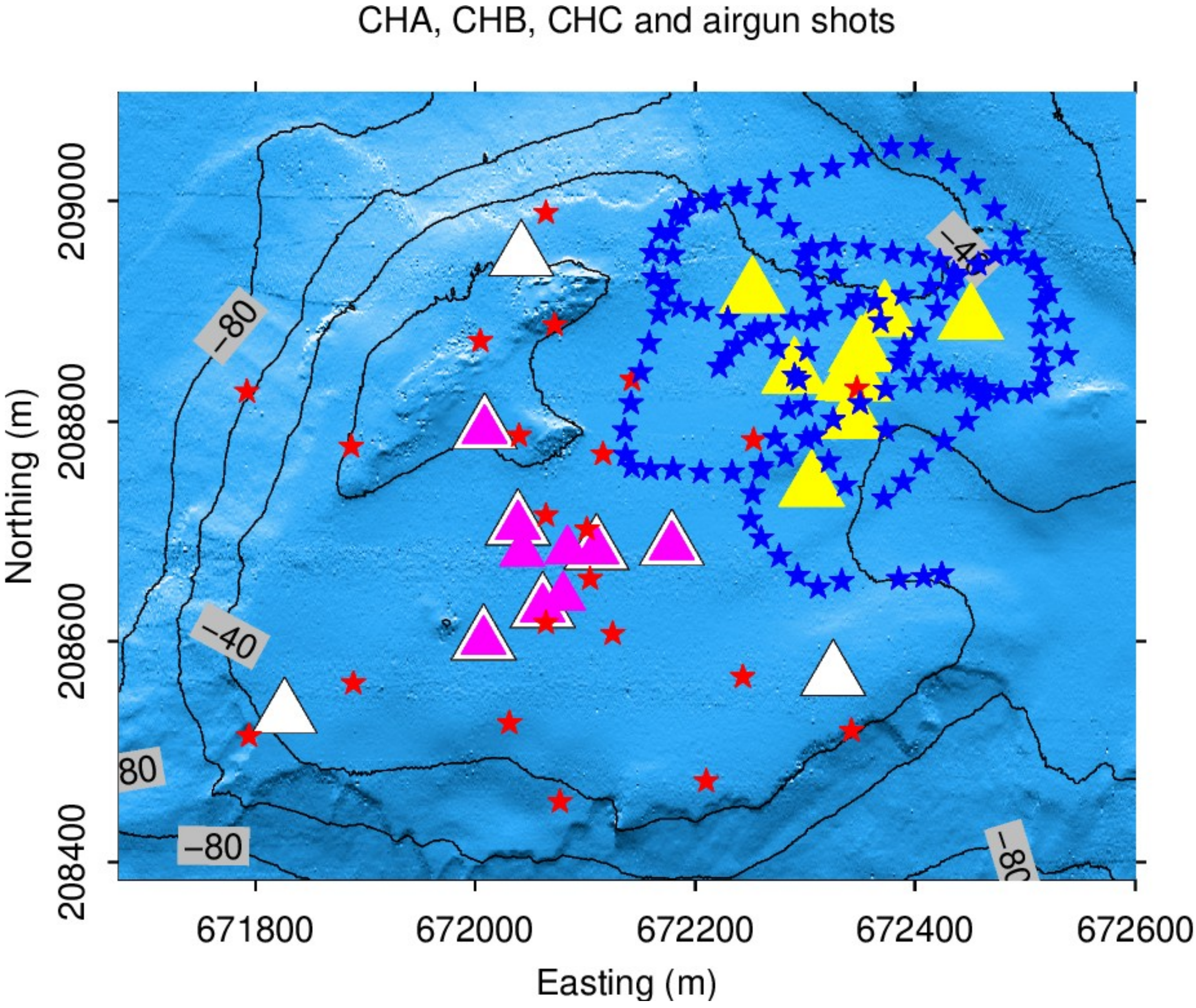

Figure C.2: Red stars indicate the airgun shooting path at CHB (magenta triangles).

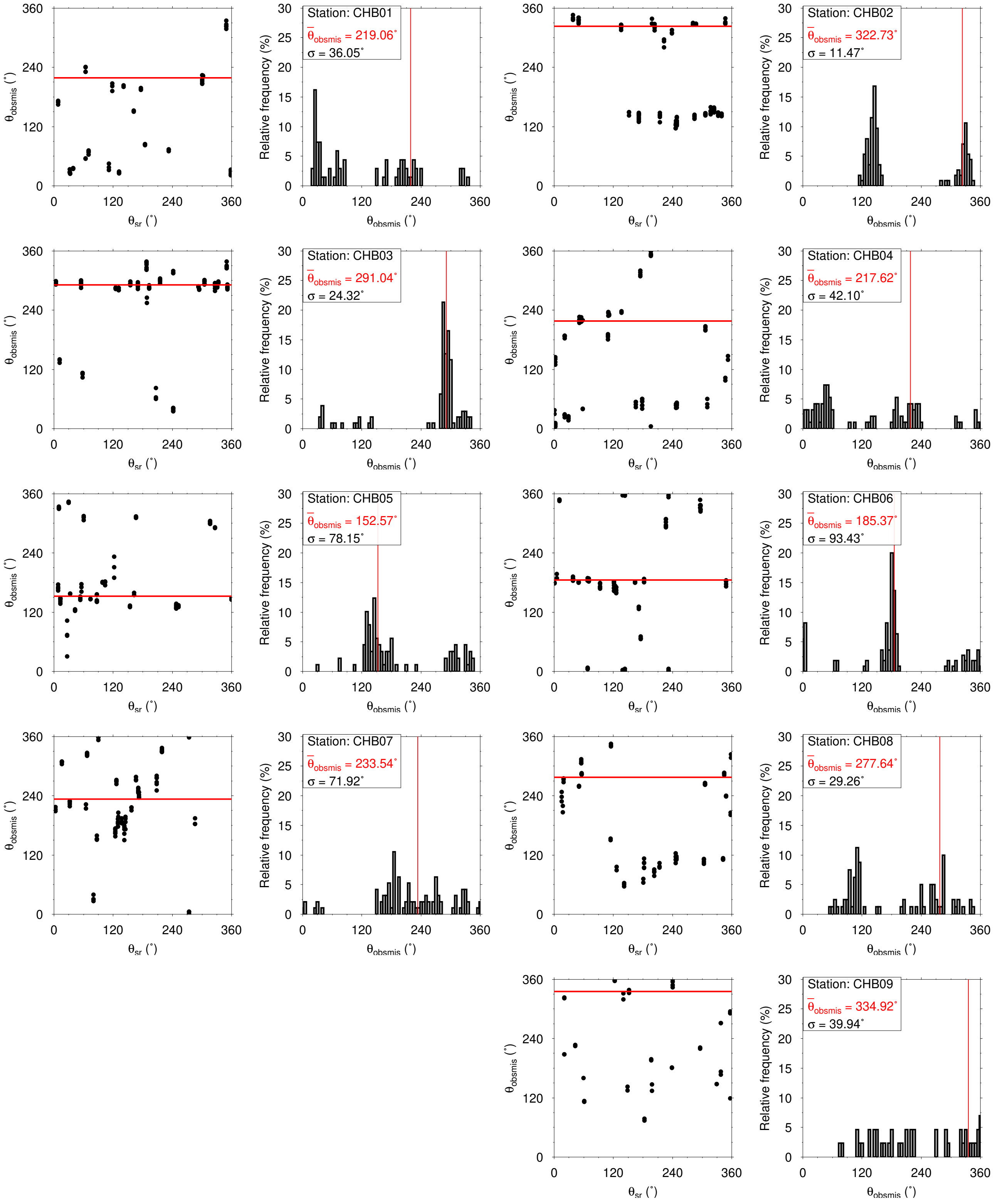

Figure C.3: Misorientation estimates at each OBS station of array CHB with respect to the shot azimuth and the corresponding relative frequency of occurrence.

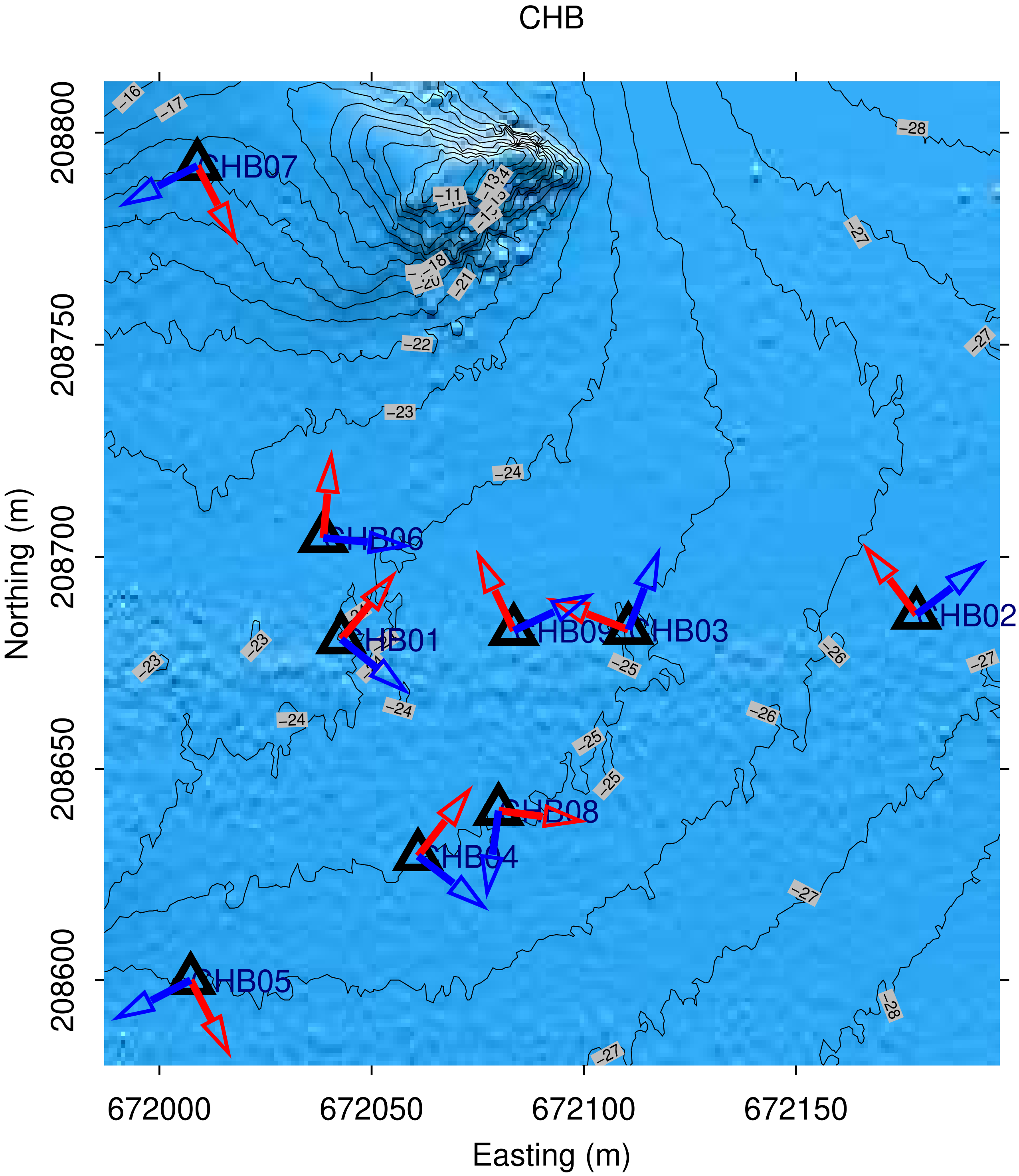

Figure C.4: Plot of the OBS horizontal component orientations on the bathymetry map for OBS stations of array CHB.

D. Chrüztrichter: Array CHC

D.1. OBS locations

See Figure 2a in the main article.

D.2. Airgun measurements

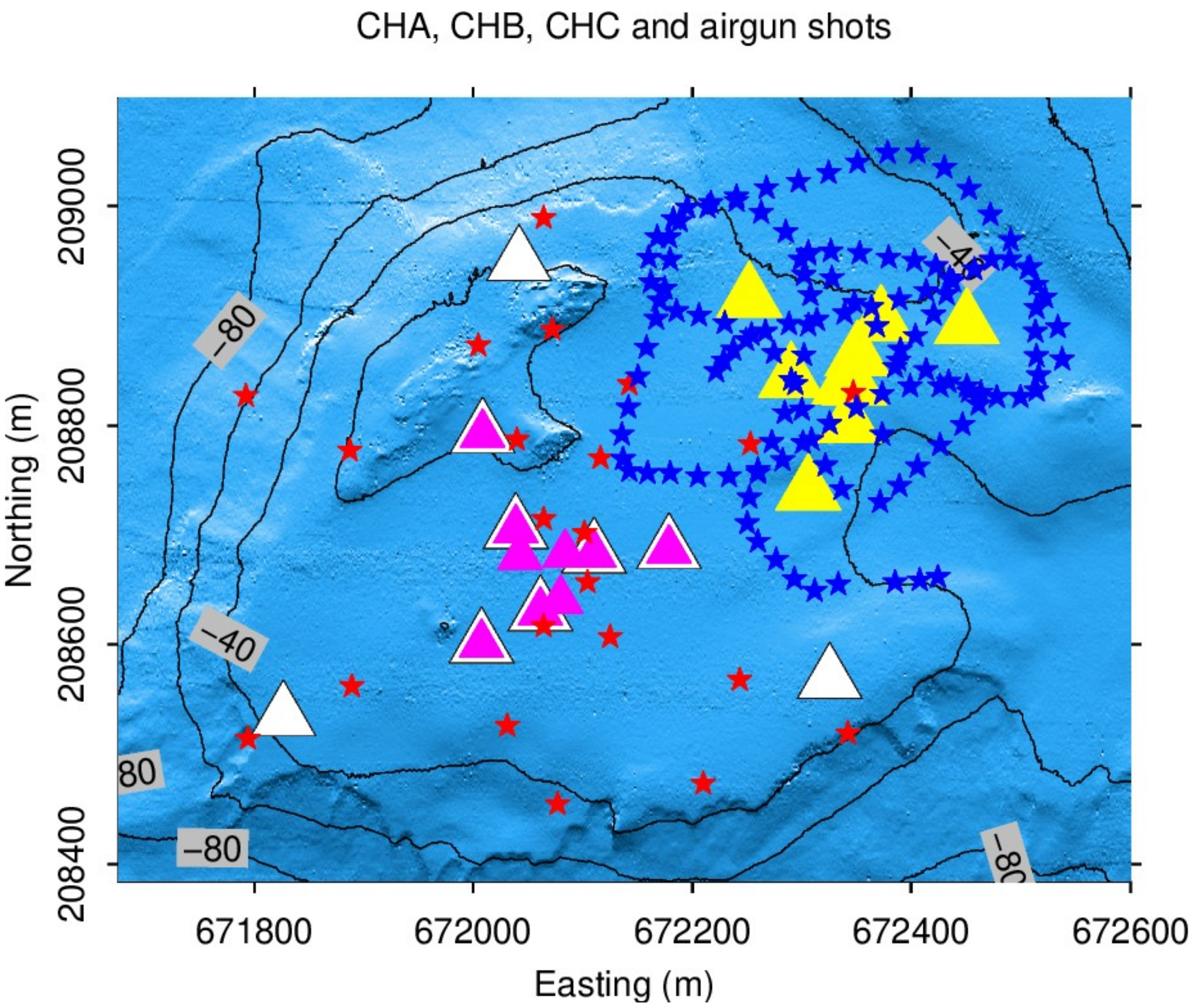

Figure D.1: Blue stars indicate the airgun shooting path at CHC (yellow triangles).

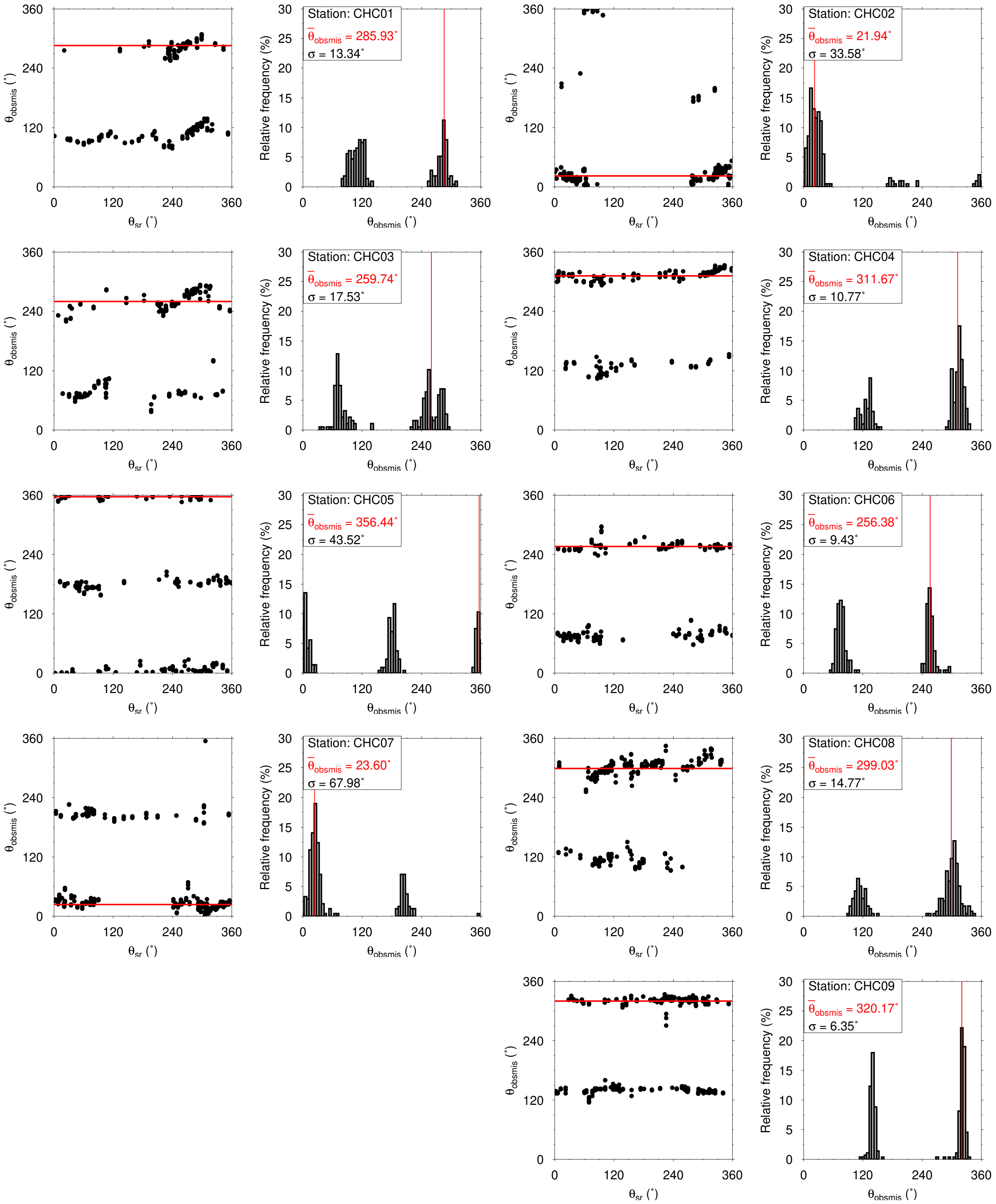

Figure D.2: Misorientation estimates at each OBS station of array CHC with respect to the shot azimuth and the corresponding relative frequency of occurrence.

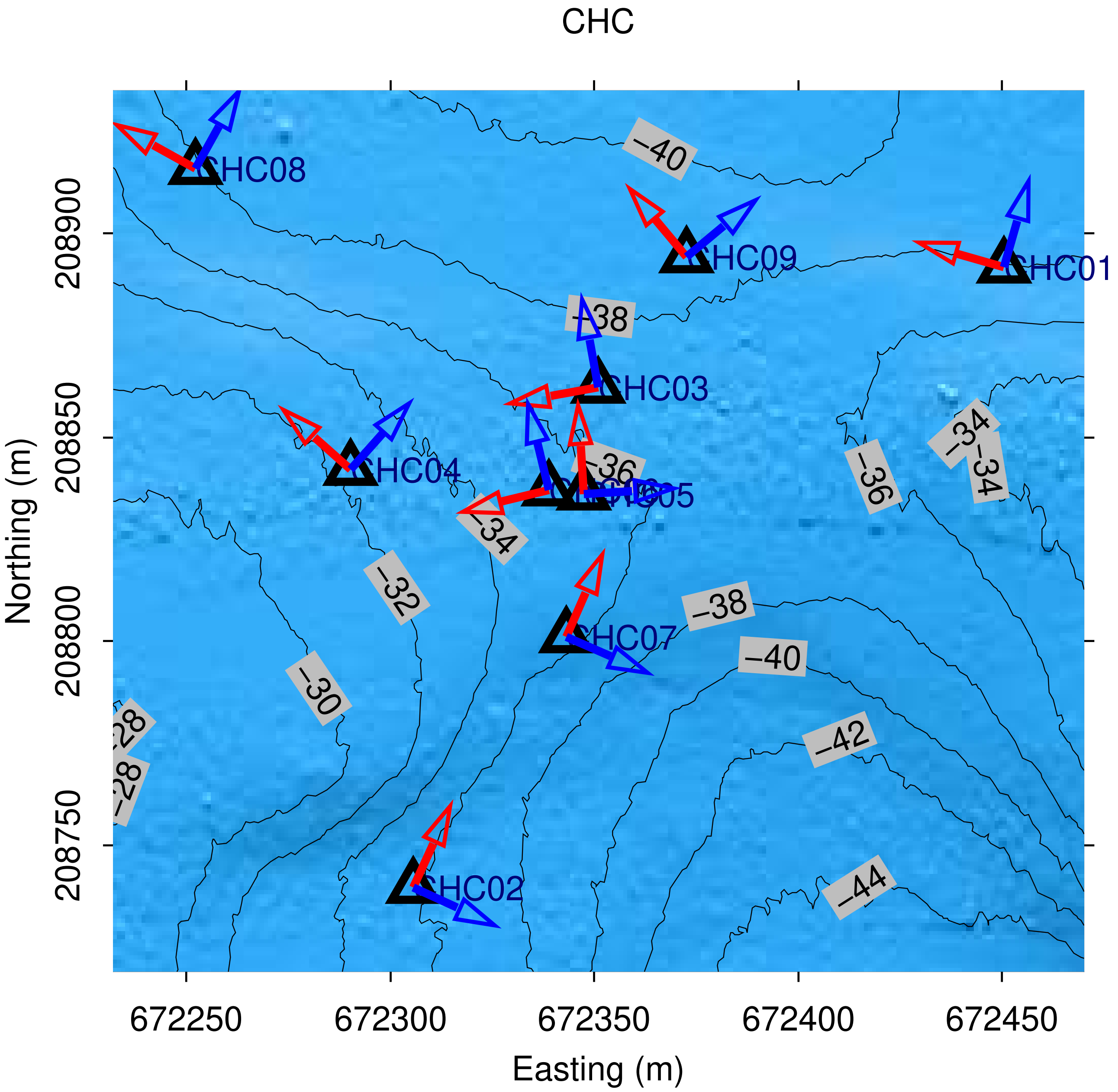

Figure D.3: Plot of the OBS horizontal component orientations on the bathymetry map for OBS stations of array CHC.

E. Chindli: Array CIA

E.1. OBS locations

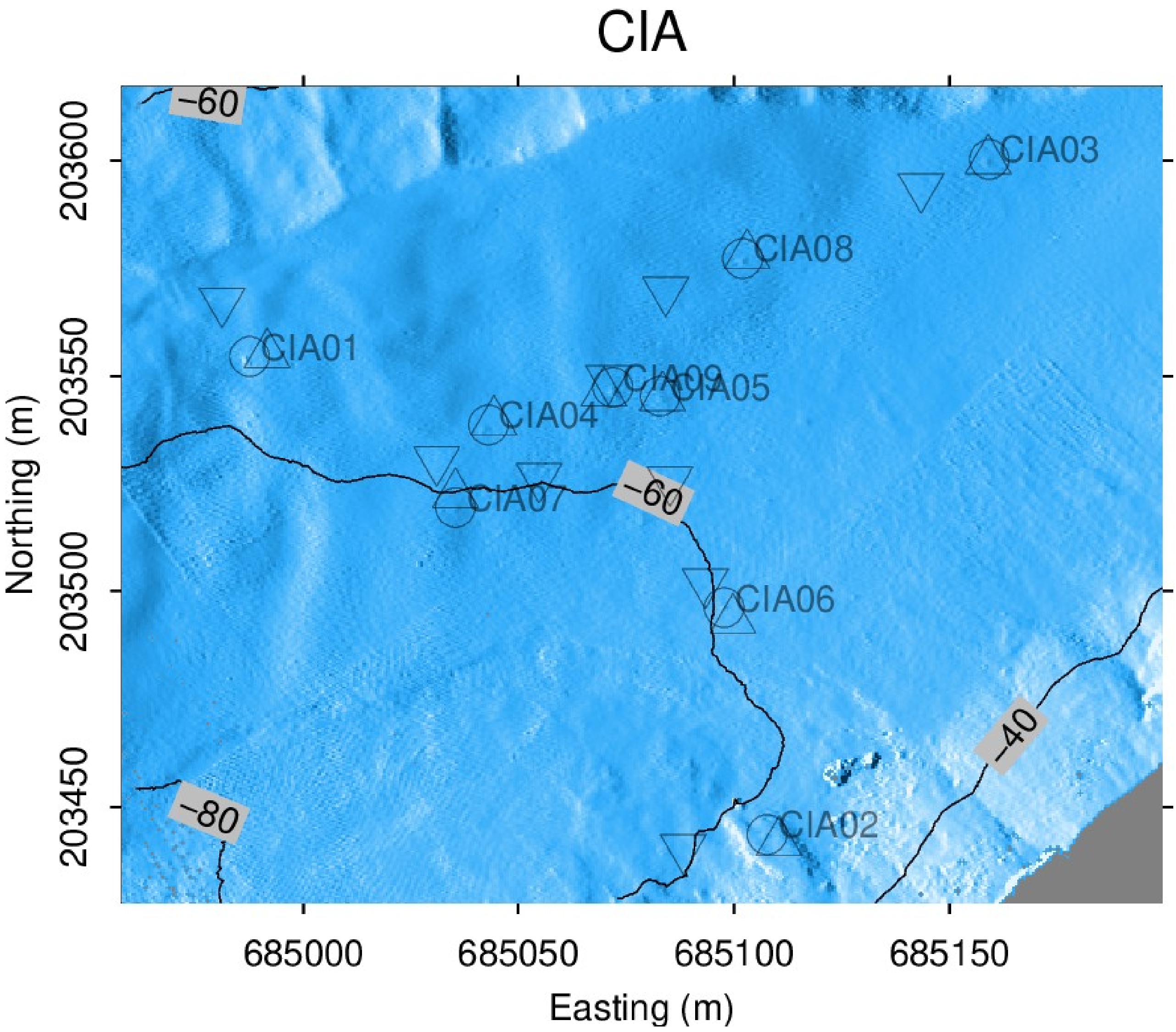

Figure E.1: OBS localization at CIA. The reverse triangle indicates the OBS position at deployment using the differential GPS (dGPS); the triangle indicate the OBS position at recovery using the dGPS; and the circle indicates the OBS position from multibeam.

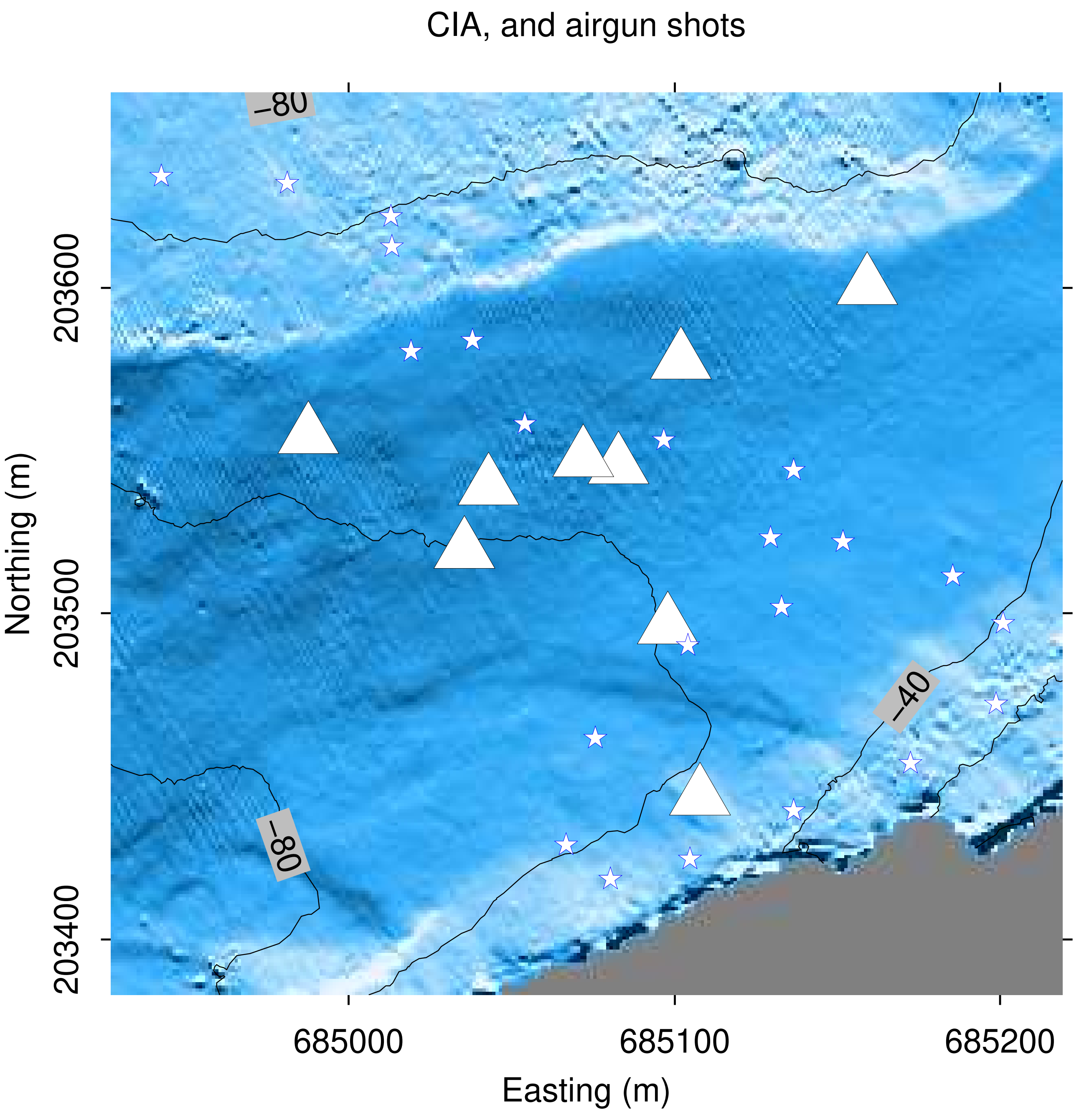

Figure E.2: The stars indicate the airgun shooting path at CIA (white triangles).

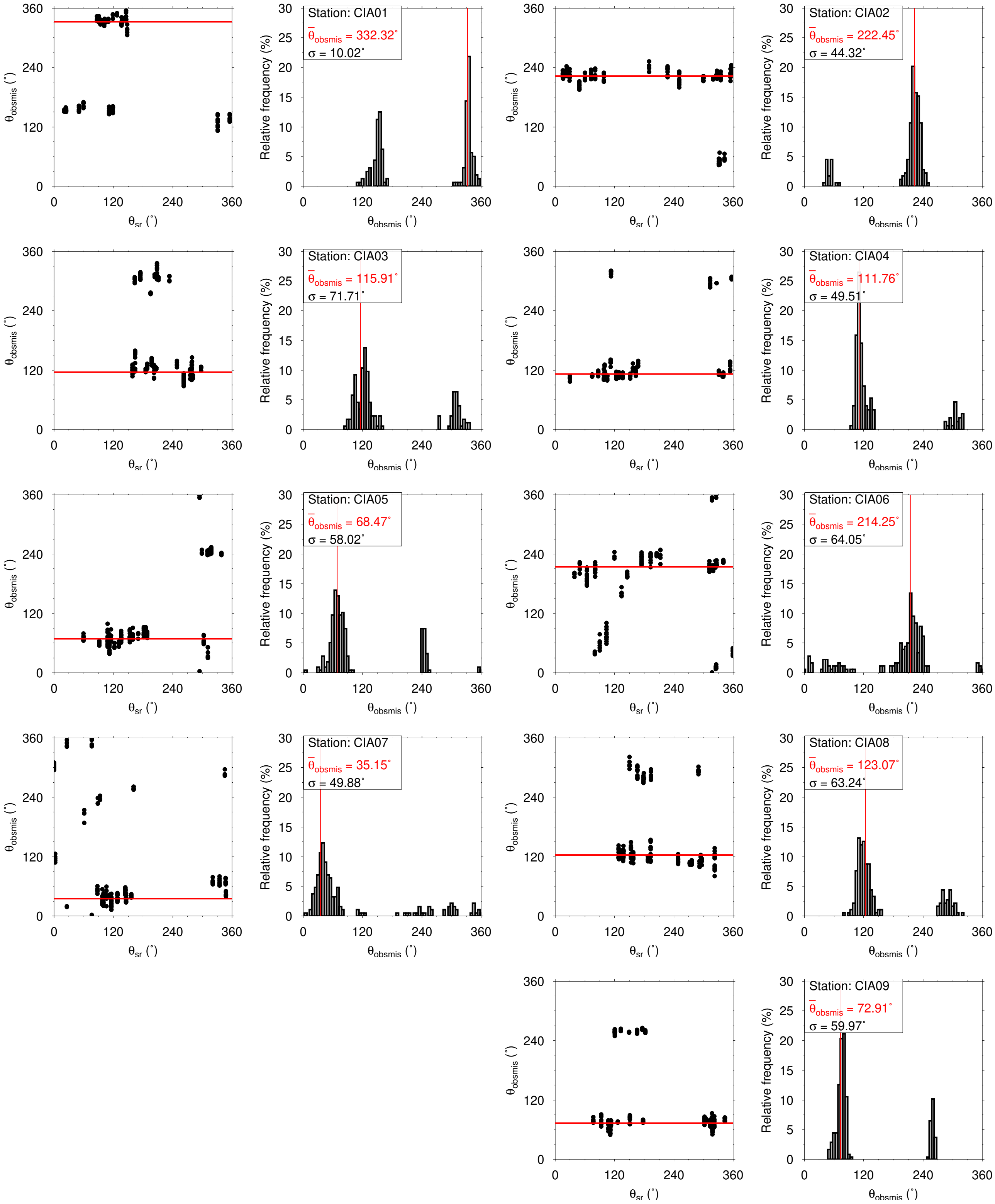

Figure E.3: Misorientation estimates at each OBS station of array CIA with respect to the shot azimuth and the corresponding relative frequency of occurrence.

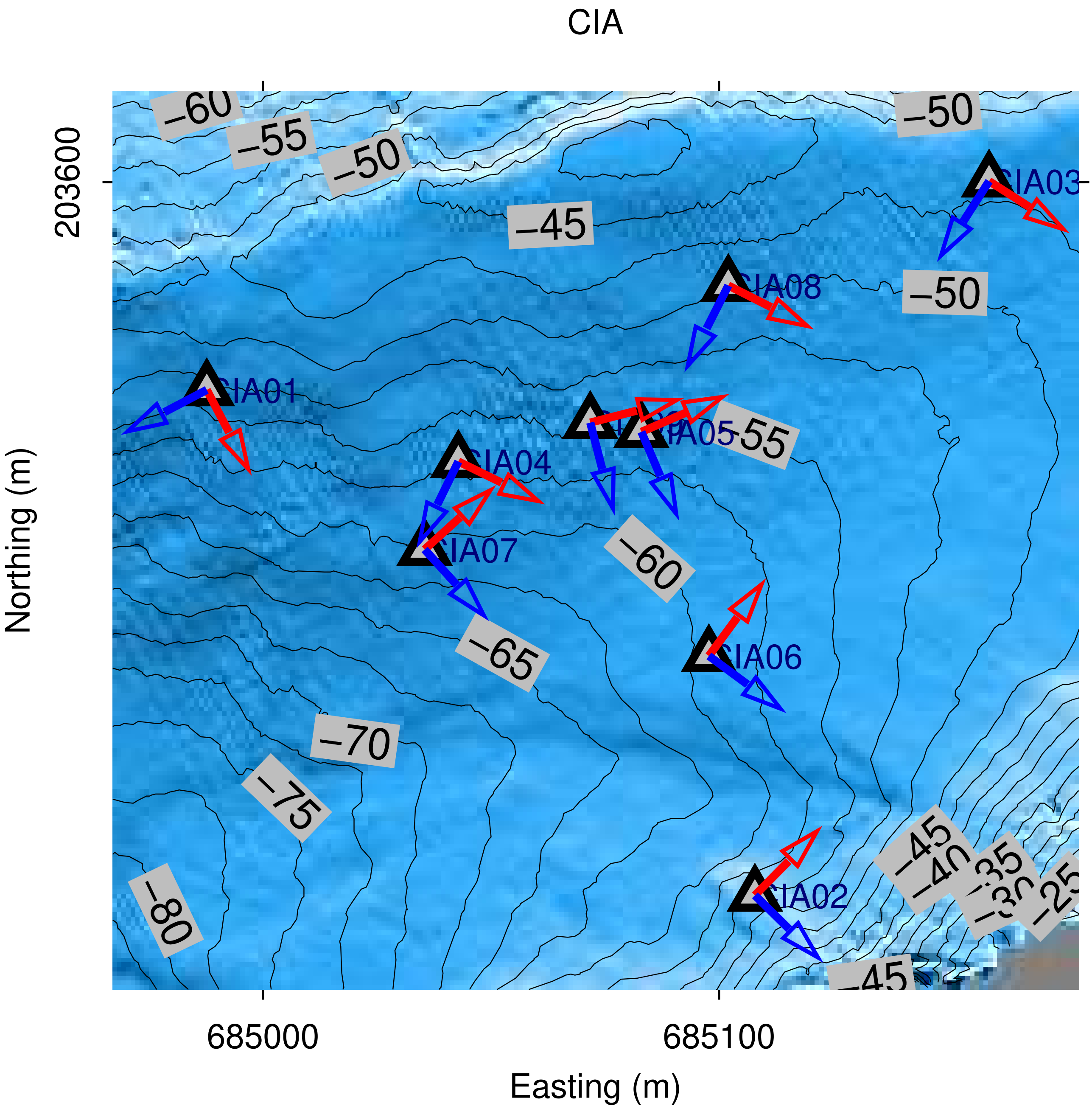

Figure E.4: Plot of the OBS horizontal component orientations on the bathymetry map for OBS stations of array CIA.

F. Ennetbürgen: Array ENA

F.1. OBS locations

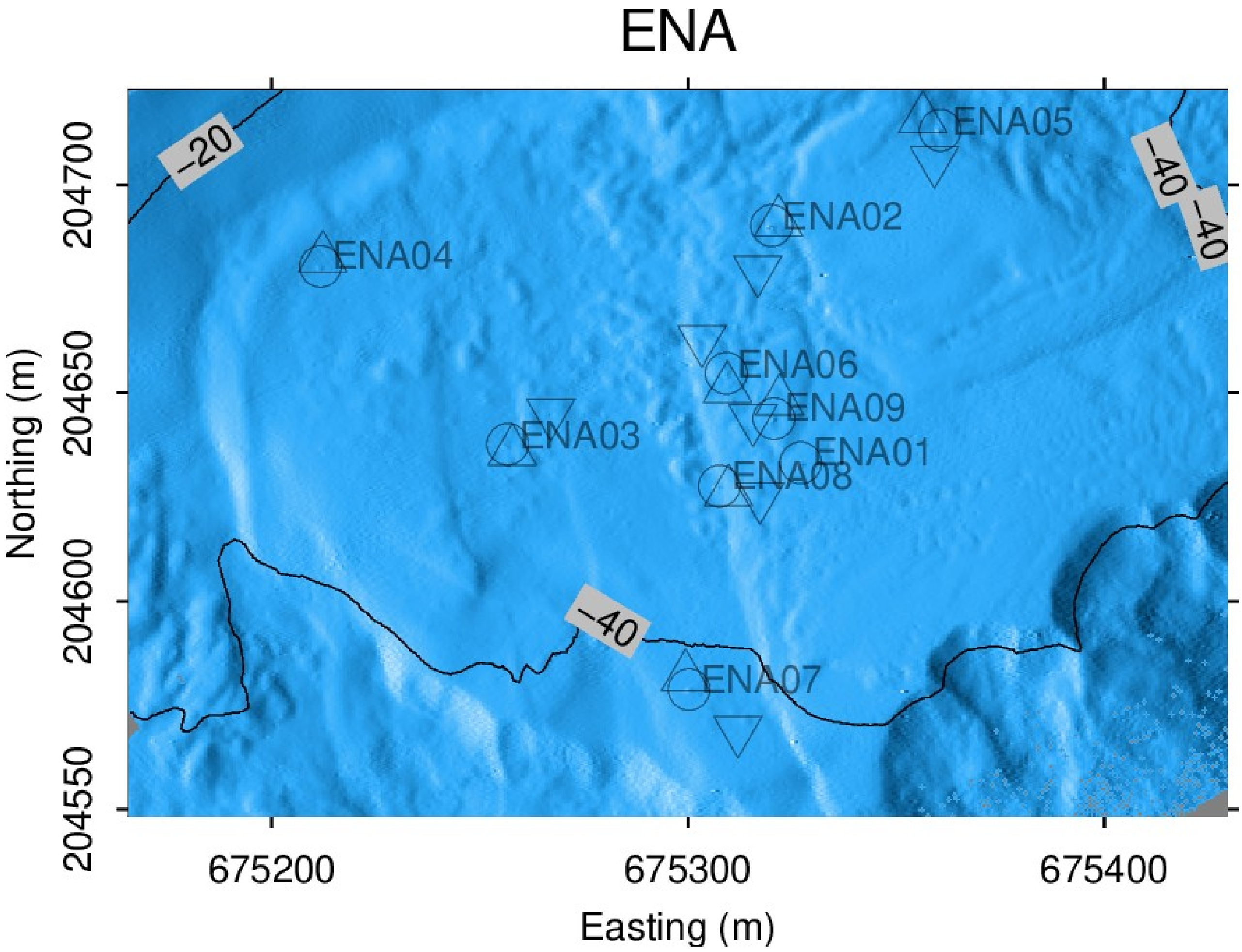

Figure F.1: OBS localization at ENA. The reverse triangle indicates the OBS position at deployment using the differential GPS (dGPS); the triangle indicate the OBS position at recovery using the dGPS; and the circle indicates the OBS position from multibeam.

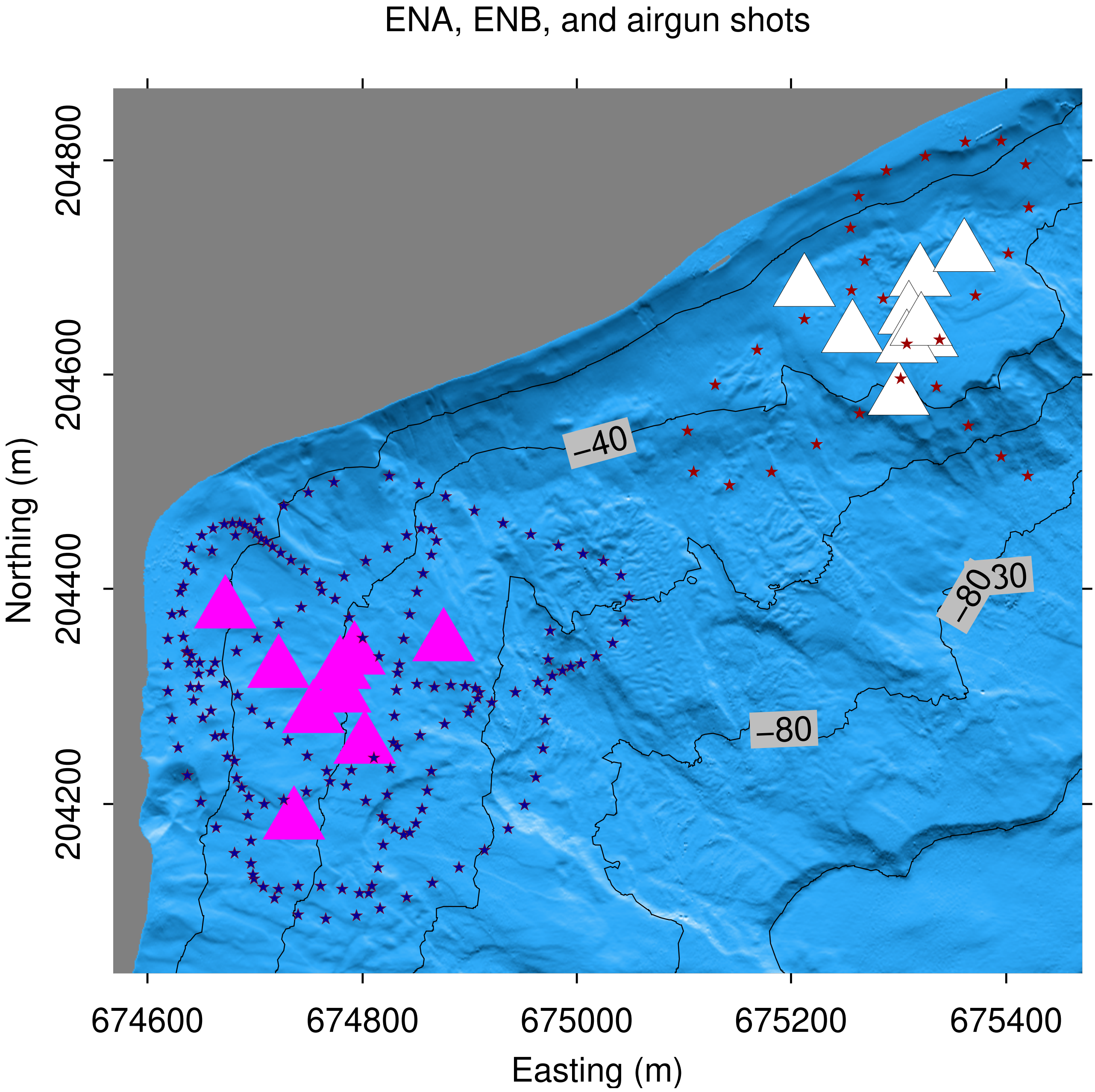

Figure F.2: The stars on top of the white triangles indicate the airgun shooting path at ENA (white triangles).

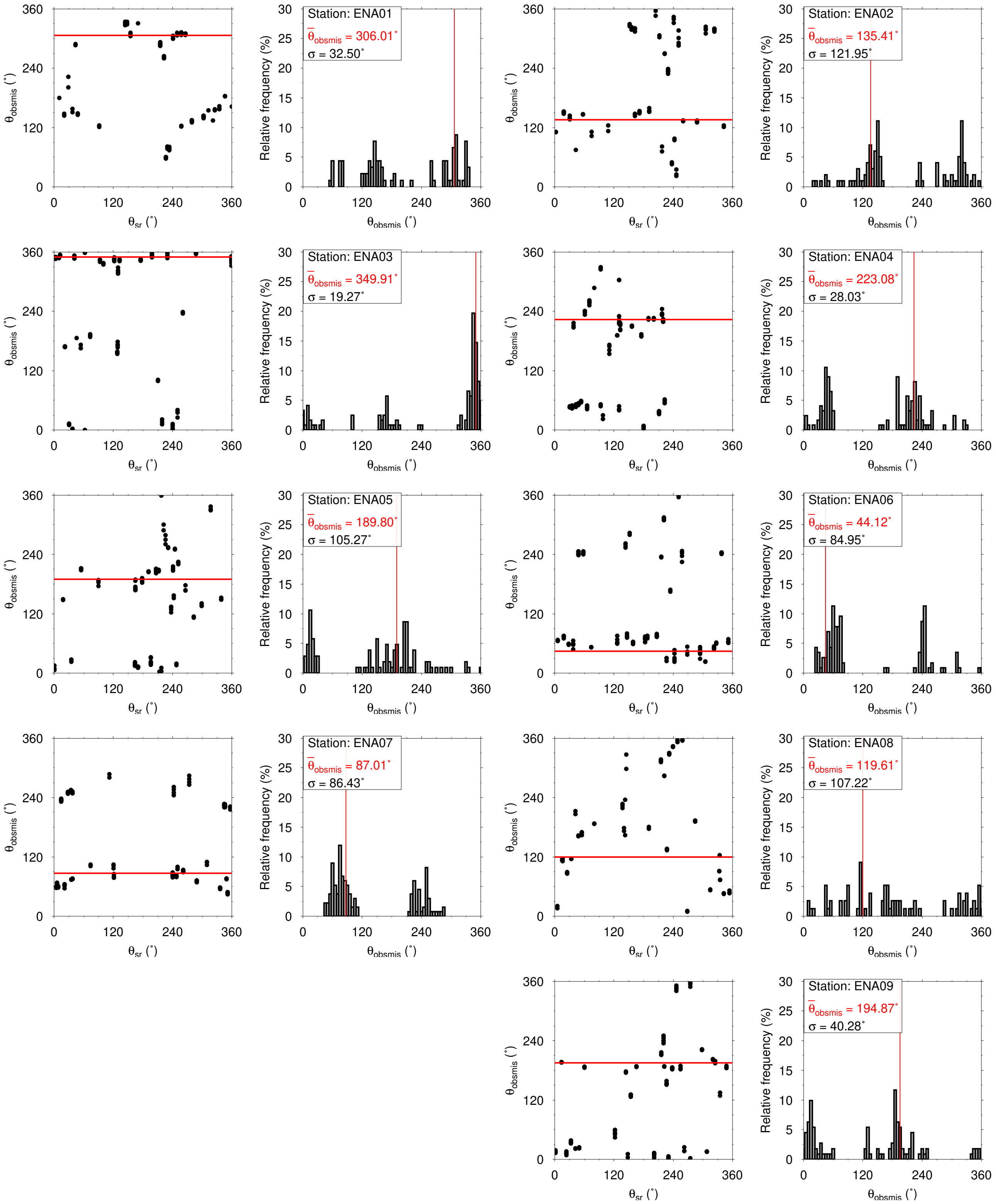

Figure F.3: Misorientation estimates at each OBS station of array ENA with respect to the shot azimuth and the corresponding relative frequency of occurrence.

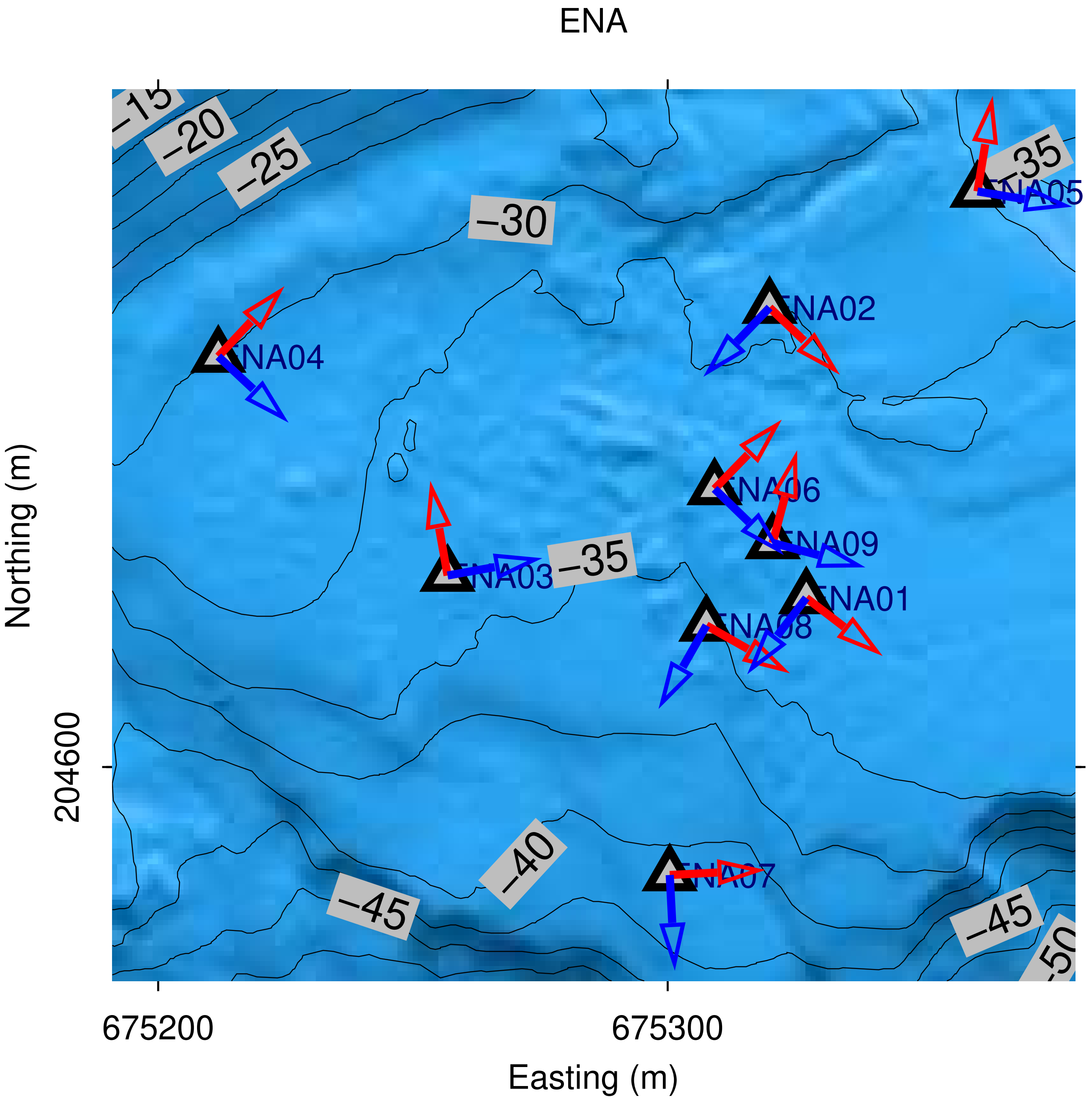

Figure F.4: Plot of the OBS horizontal component orientations on the bathymetry map for OBS stations of array ENA.

G. Ennetbürgen: Array ENB

G.1. OBS locations

See Figure 2b in the main article.

G.2. Airgun measurements

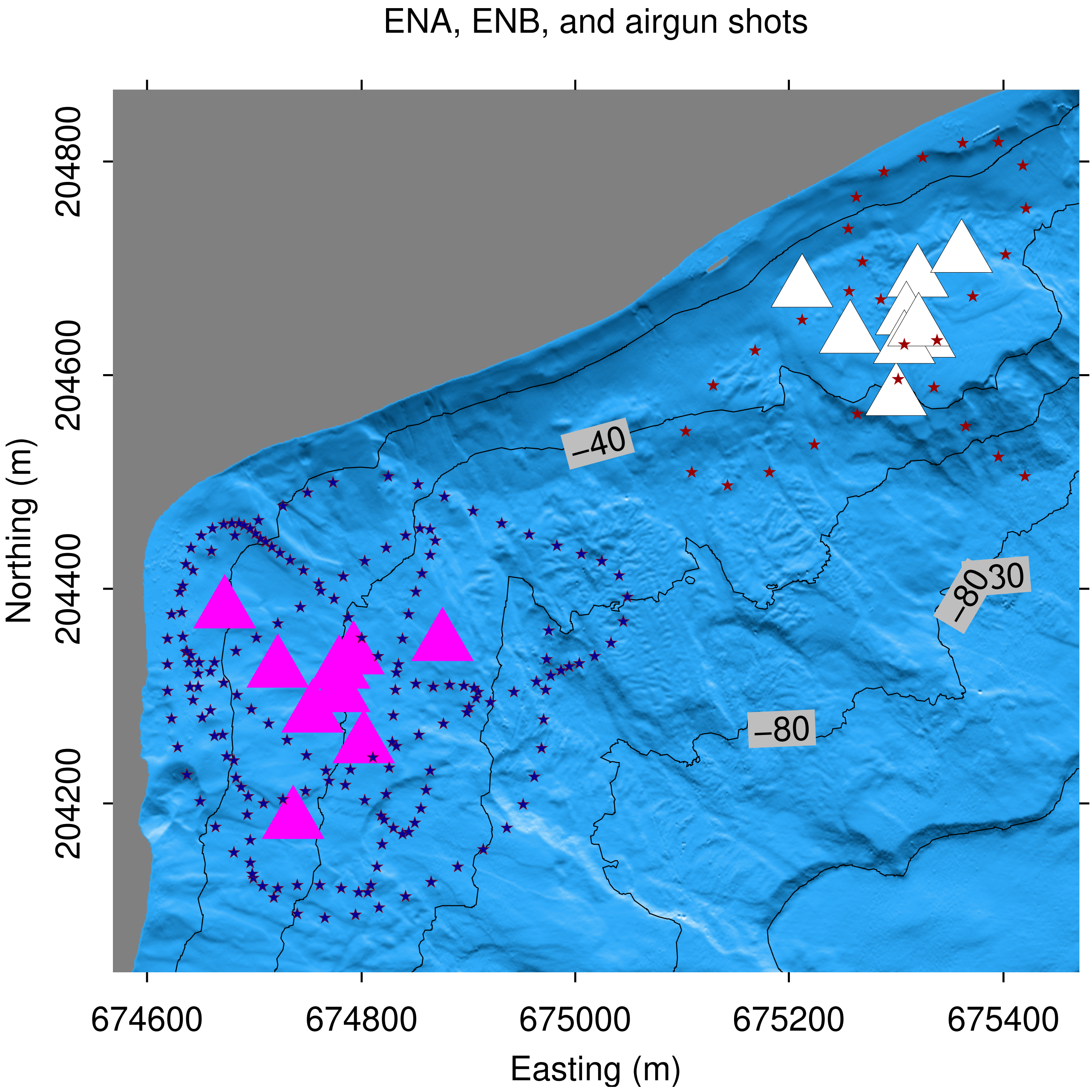

Figure G.1: The stars on top of the magenta triangles indicate the airgun shooting path at ENB (magenta triangles).

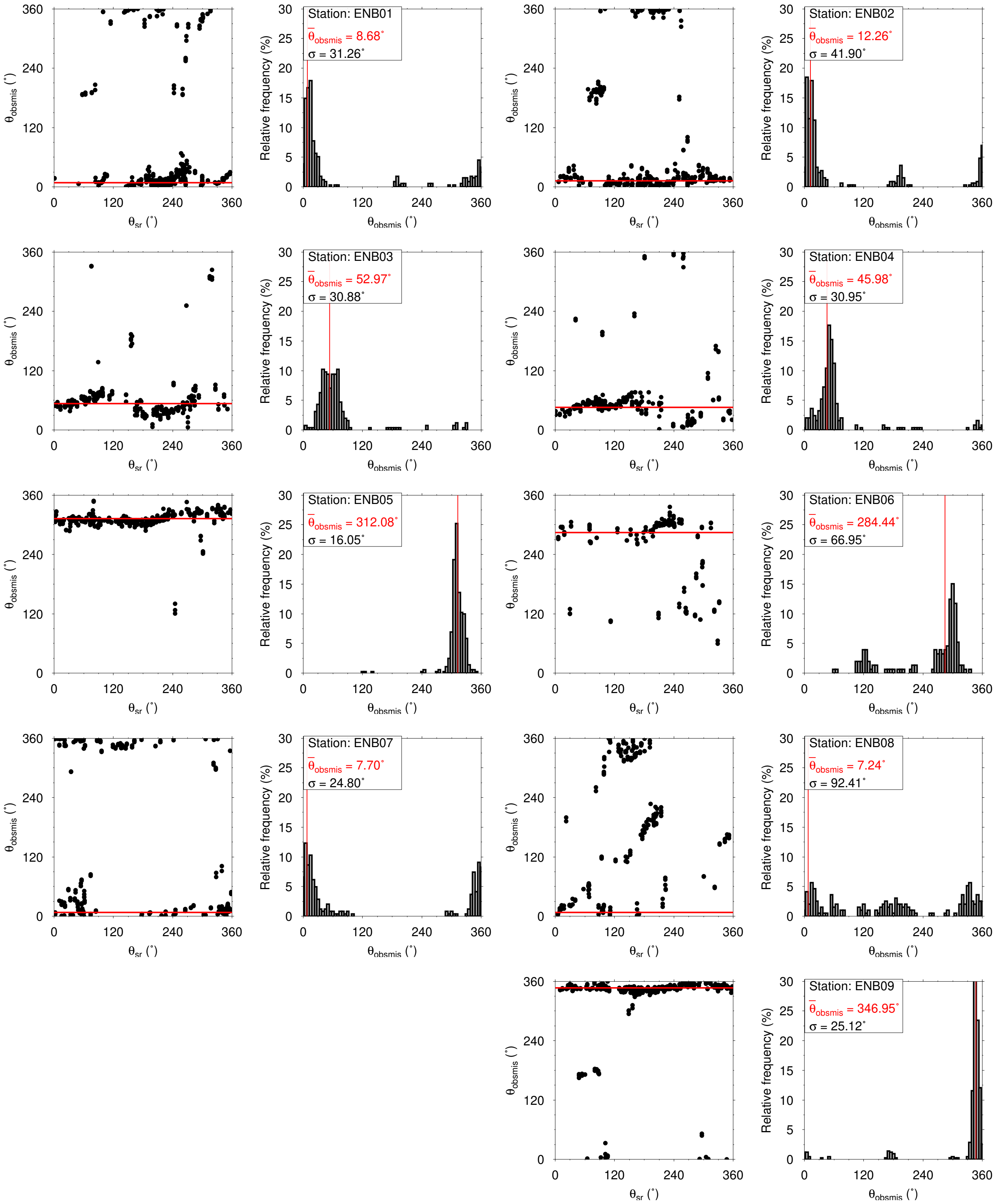

Figure G.2: Misorientation estimates at each OBS station of array ENB with respect to the shot azimuth and the corresponding relative frequency of occurrence.

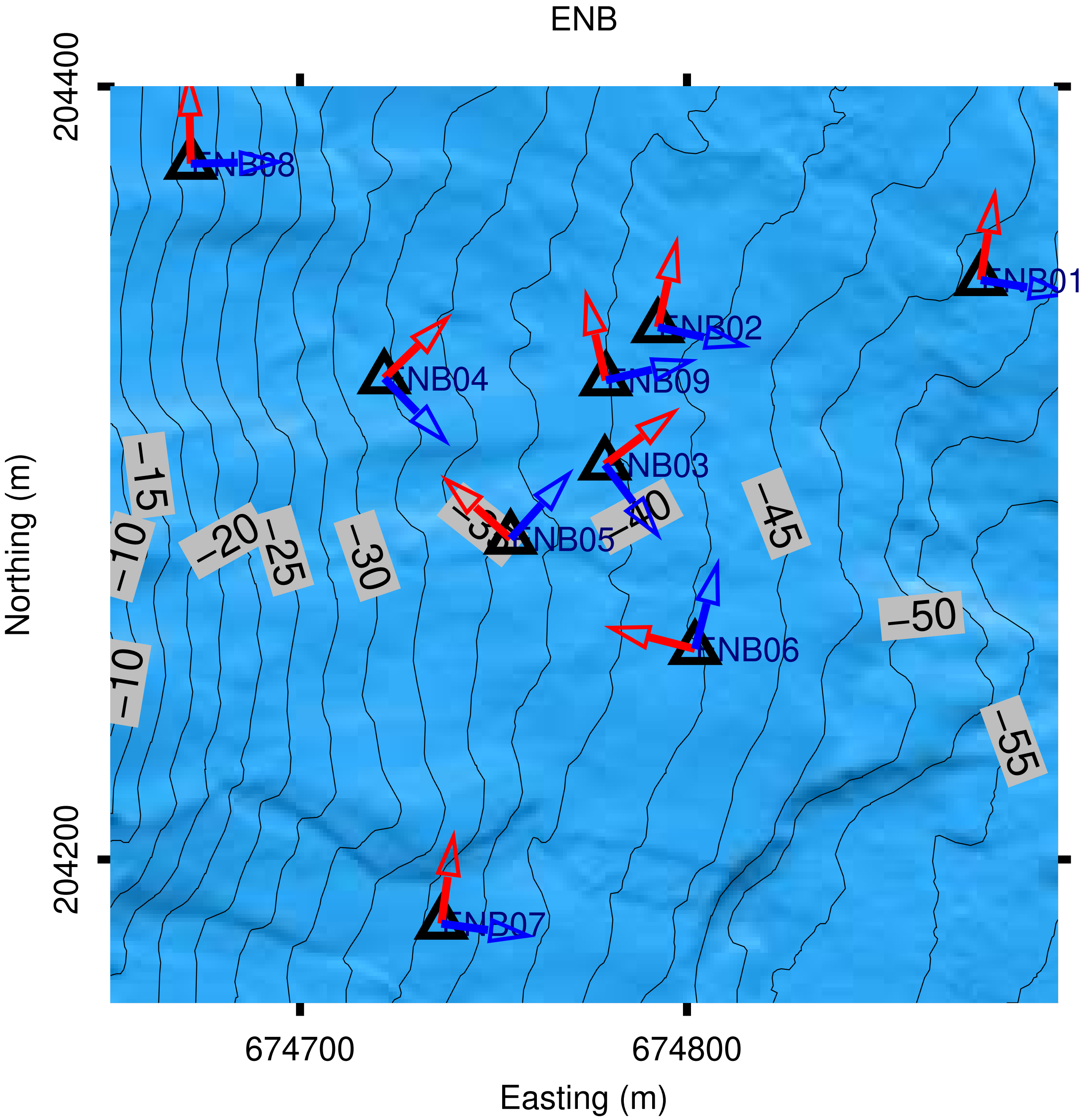

Figure G.3: Plot of the OBS horizontal component orientations on the bathymetry map for OBS stations of array ENB.

H. Kehrsiten: Array KEA

H.1. OBS locations

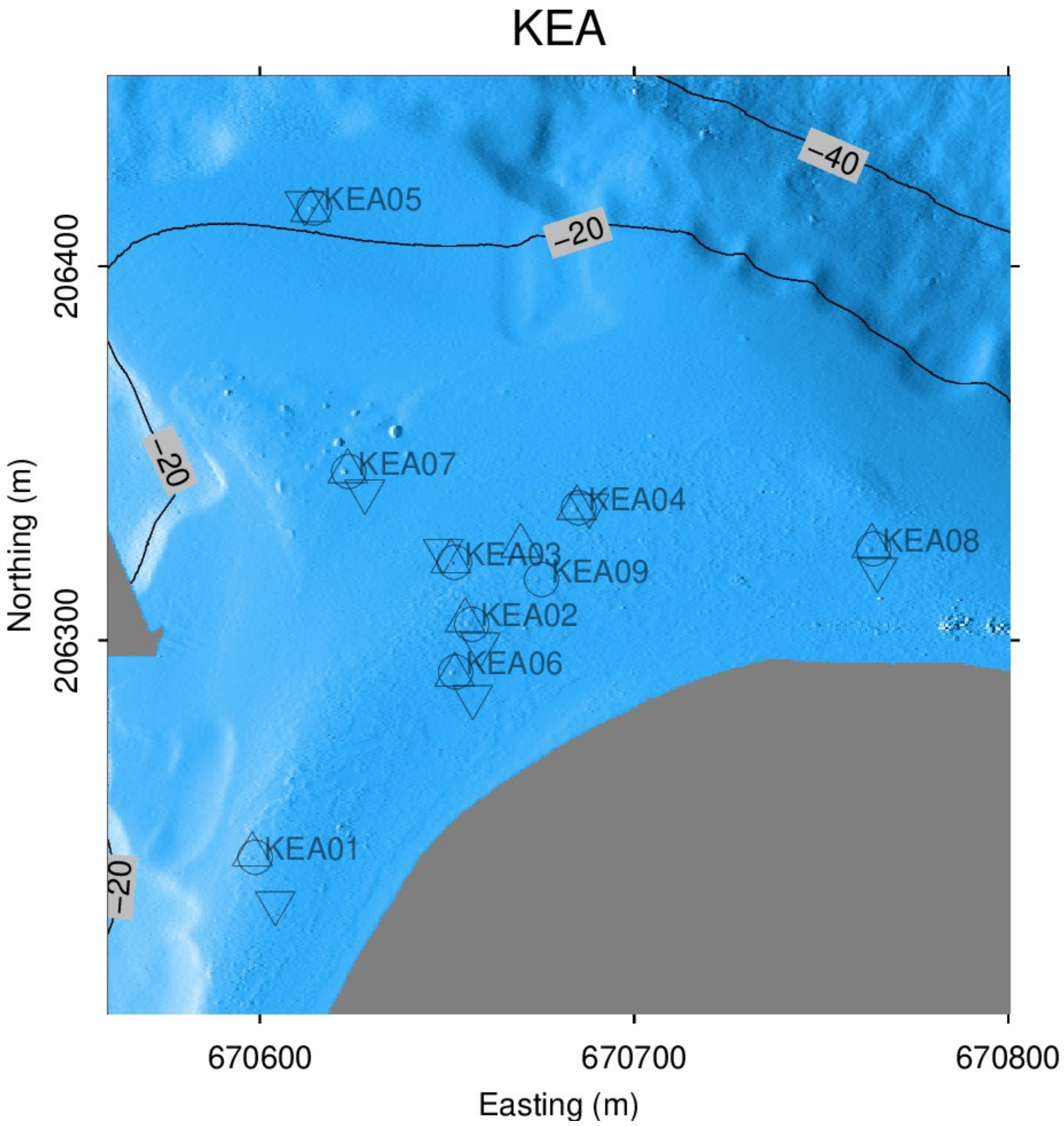

Figure H.1: OBS localization at KEA. The reverse triangle indicates the OBS position at deployment using the dGPS; the triangle indicate the OBS position at recovery using the dGPS; and the circle indicates the OBS position from multibeam.

H.2. Airgun measurements

No airgun measurement available for this site.

H.3. OBS misorientation estimation

No obsmis values available at KEA.

H.4. OBS misorientation with base bathymetry

No obsmis values available at KEA.

I. Kehrsiten: Array KEB

I.1. OBS locations

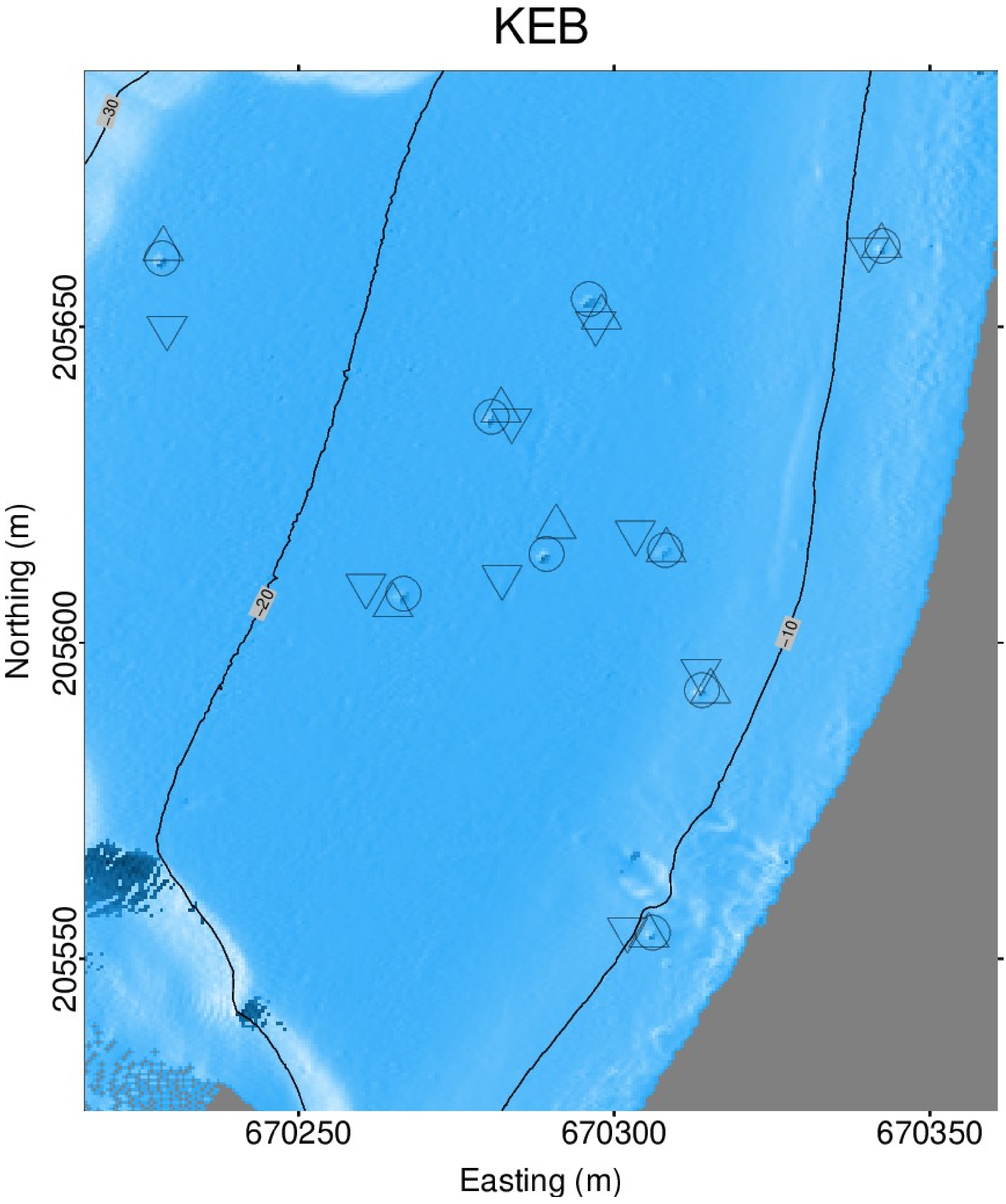

Figure I.1: OBS localization at KEB. The reverse triangle indicates the OBS position at deployment using the differential GPS (dGPS); the triangle indicate the OBS position at recovery using the dGPS; and the circle indicates the OBS position from multibeam.

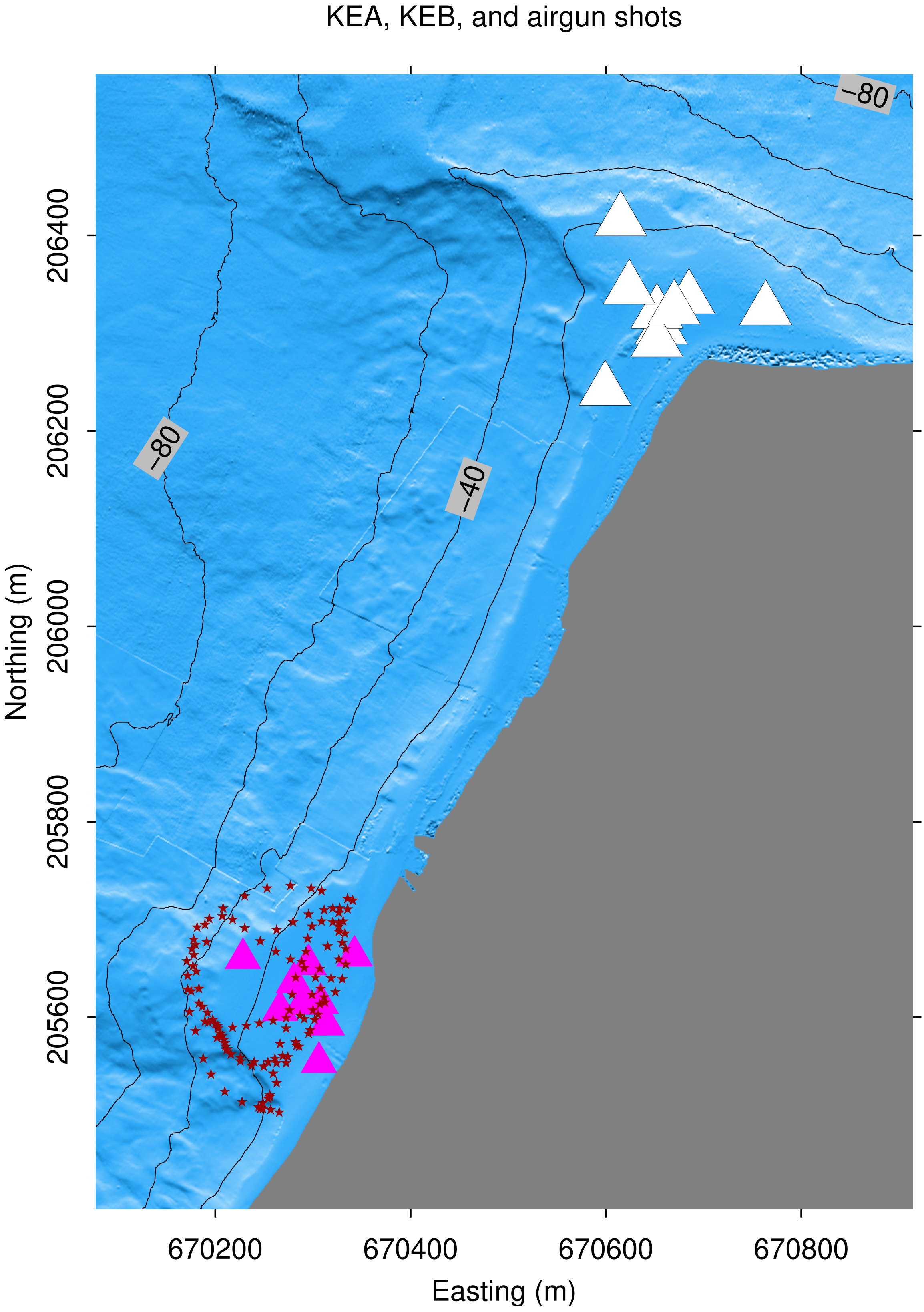

Figure I.2: The stars on top of the magenta triangles indicate the airgun shooting path at KEB (magenta triangles).

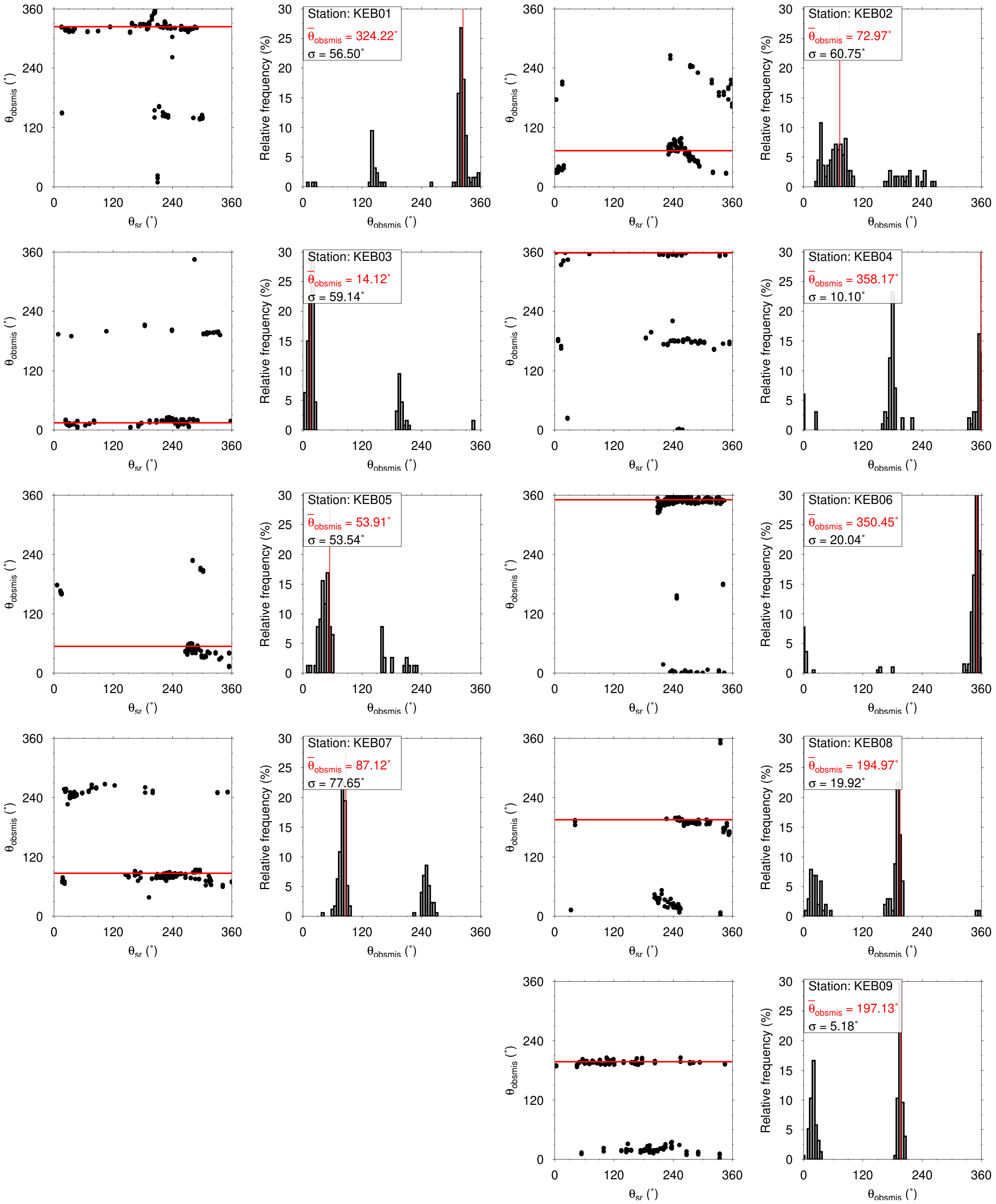

Figure I.3: Misorientation estimates at each OBS station of array KEB with respect to the shot azimuth and the corresponding relative frequency of occurrence.

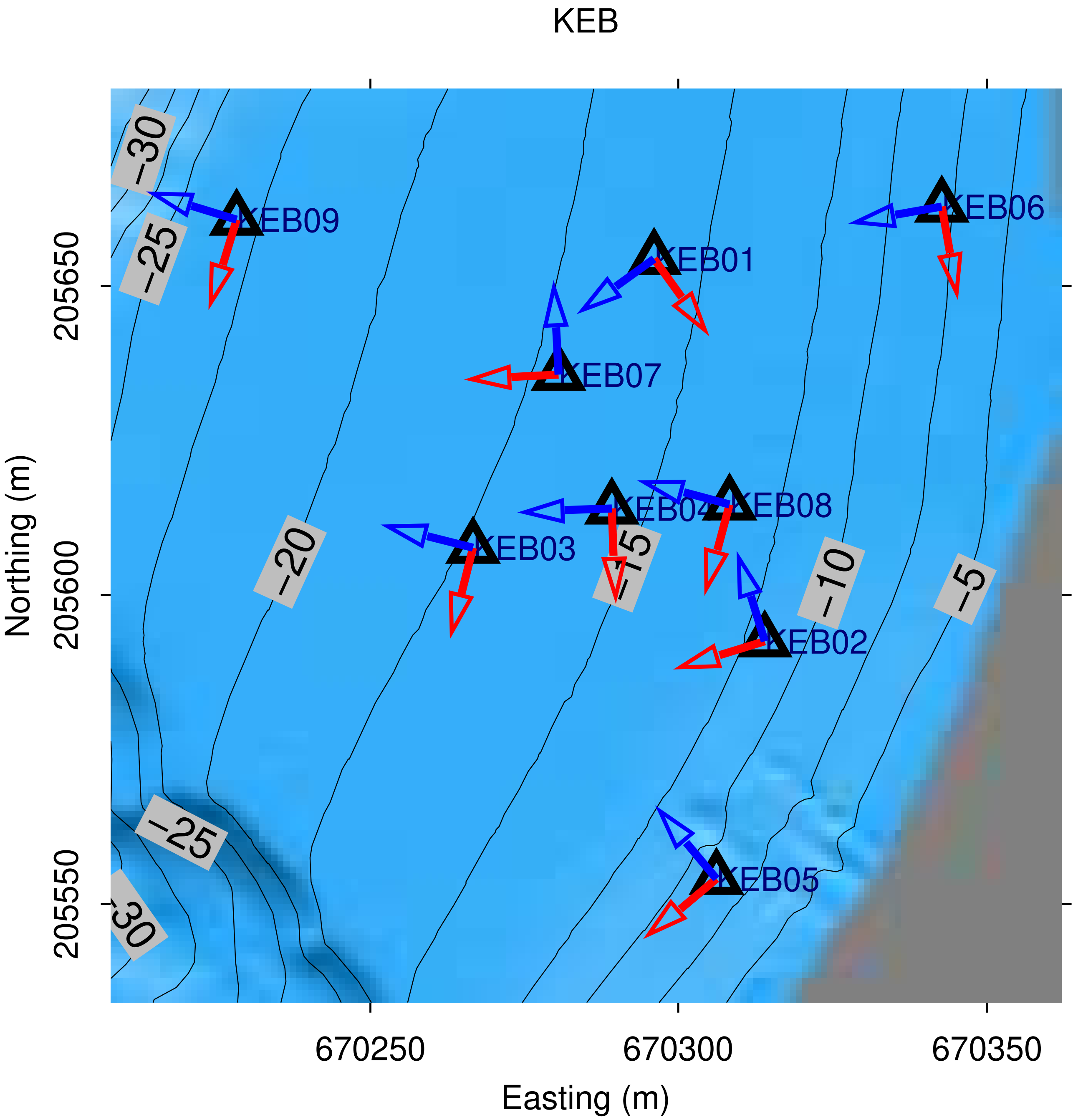

Figure I.4: Plot of the OBS horizontal component orientations on the bathymetry map for OBS stations of array CHB.

J. Muota: Array MUA

J.1. OBS locations

See Figure 2c in the main article.

J.2. Airgun measurements

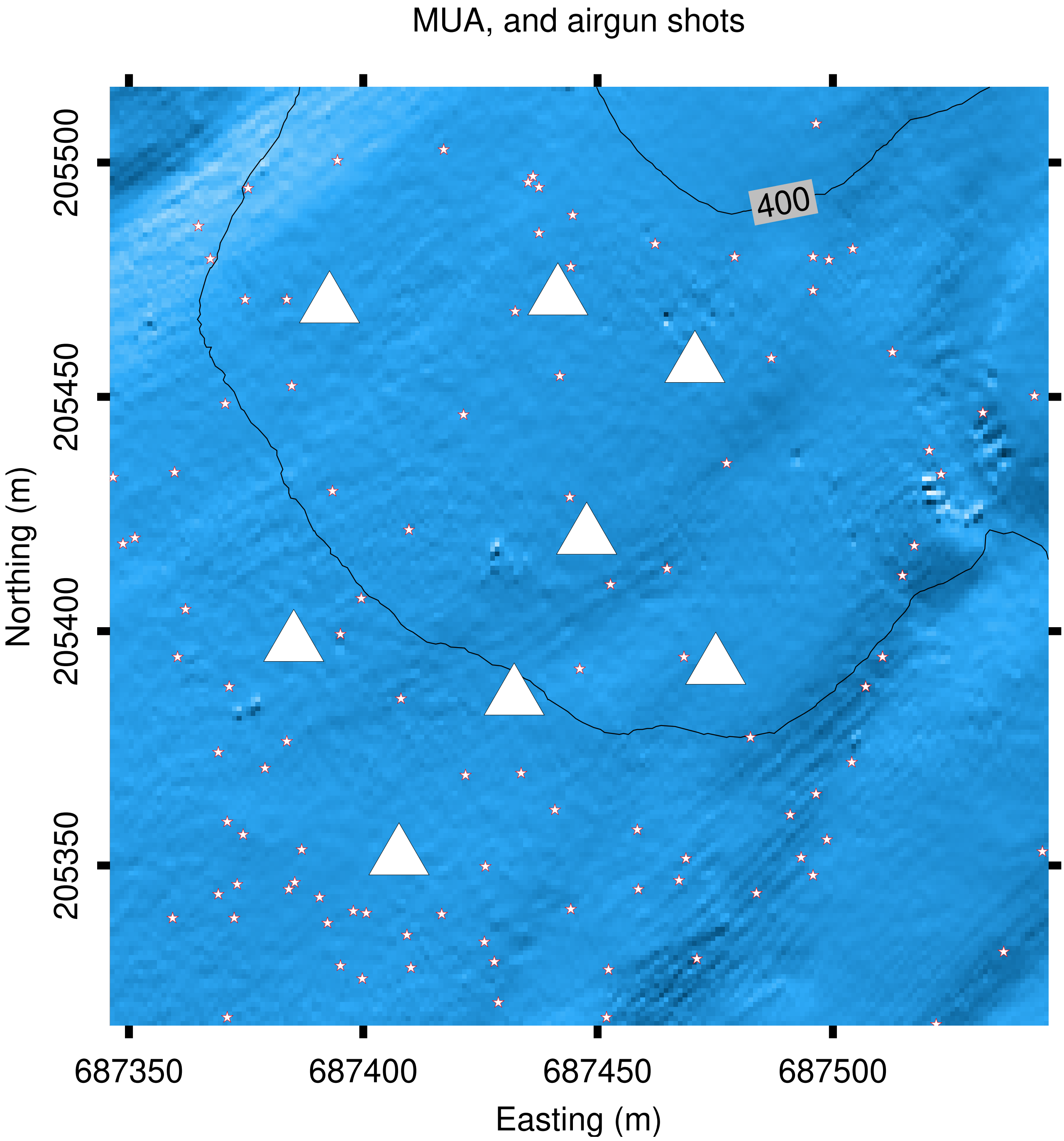

Figure J.1: Red stars indicate the airgun shooting path at MUA (white triangles).

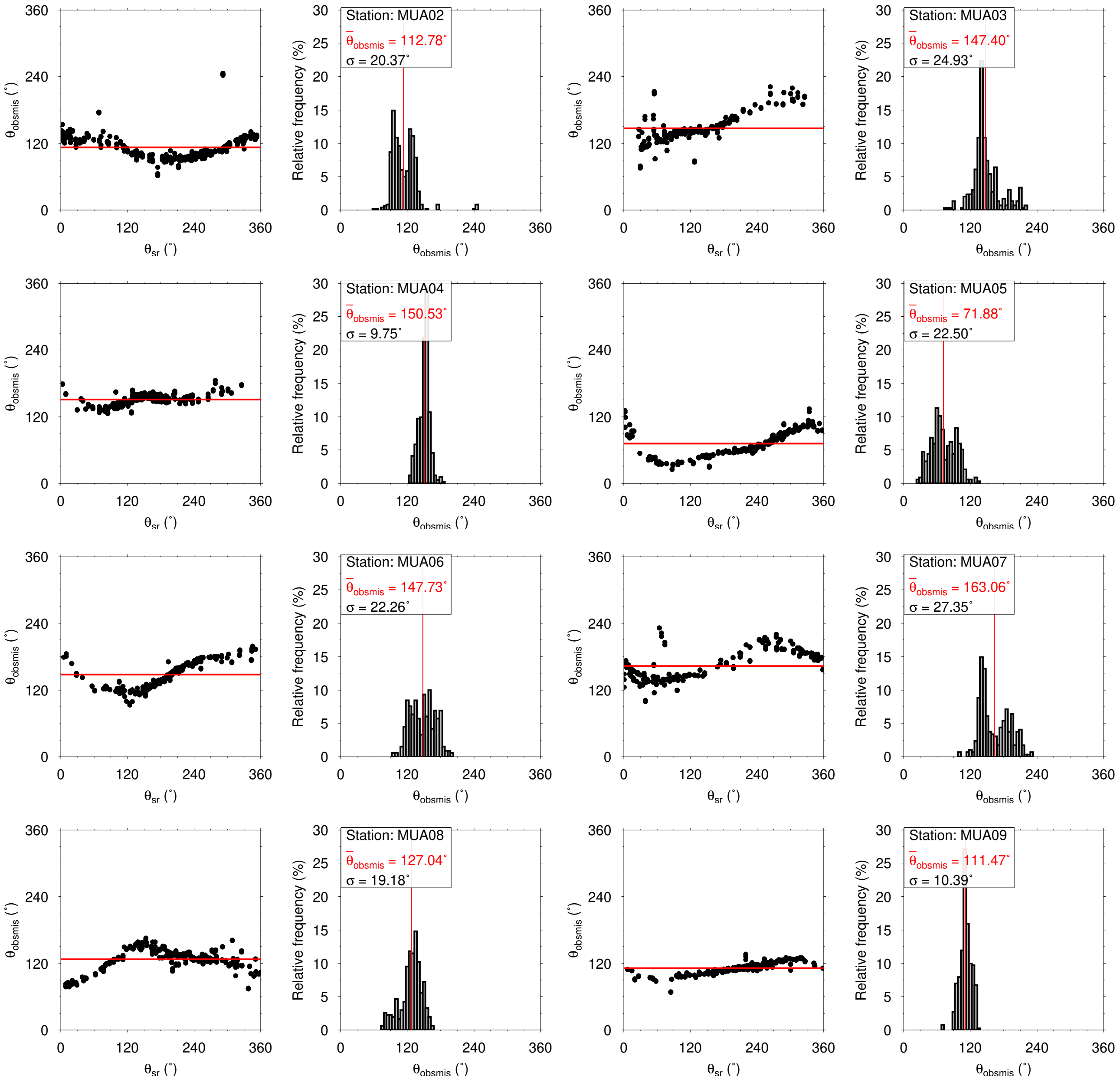

Figure J.2: Misorientation estimates at each OBS station of array MUA with respect to the shot azimuth and the corresponding relative frequency of occurrence.

J.4. OBS misorientation with base bathymetry

See Figure 6 in the main article.

K. Nase: Array NAA

K.1. OBS locations

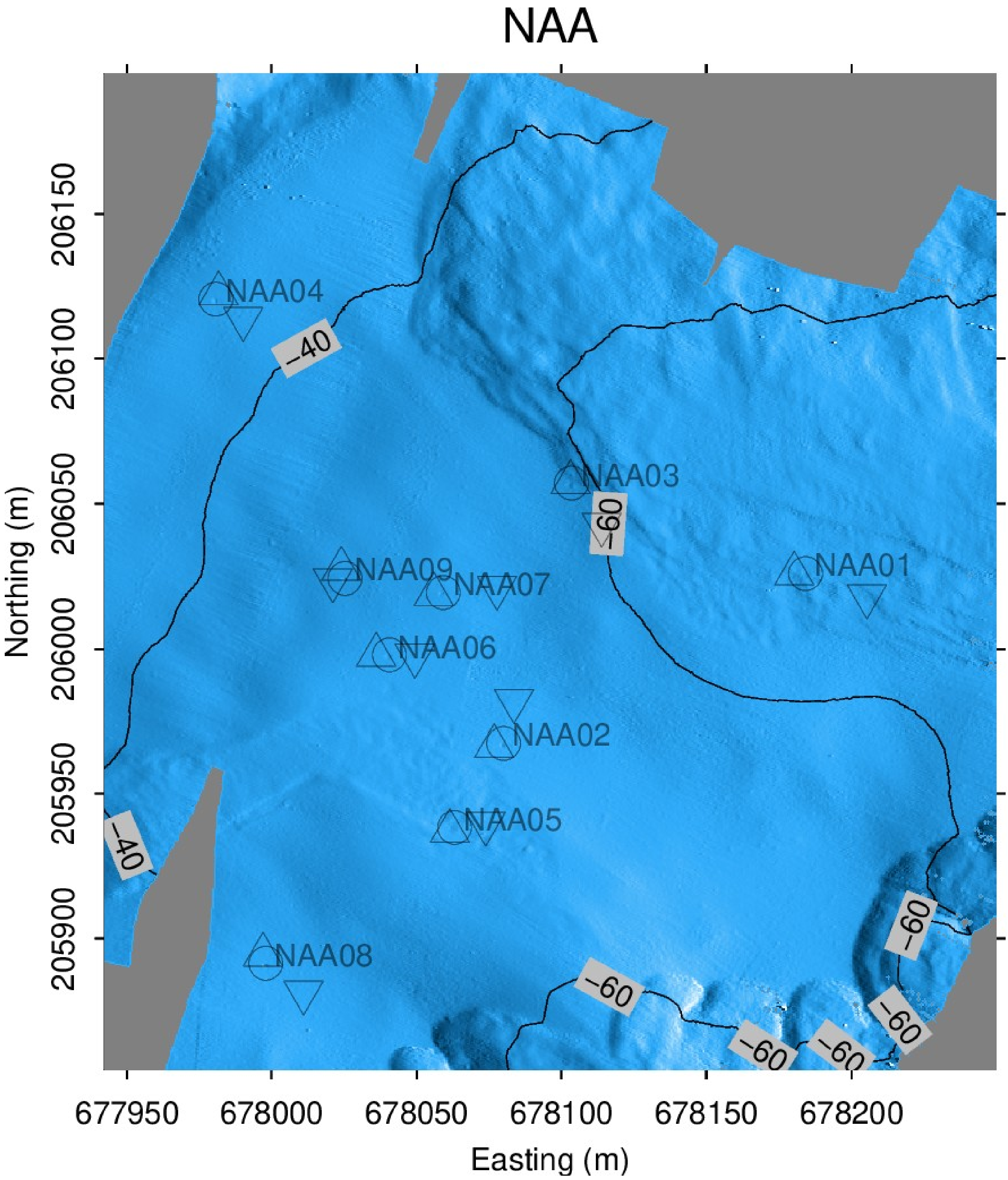

Figure K.1: OBS localization at NAA. The reverse triangle indicates the OBS position at deployment using the differential GPS (dGPS); the triangle indicate the OBS position at recovery using the dGPS; and the circle indicates the OBS position from multibeam.

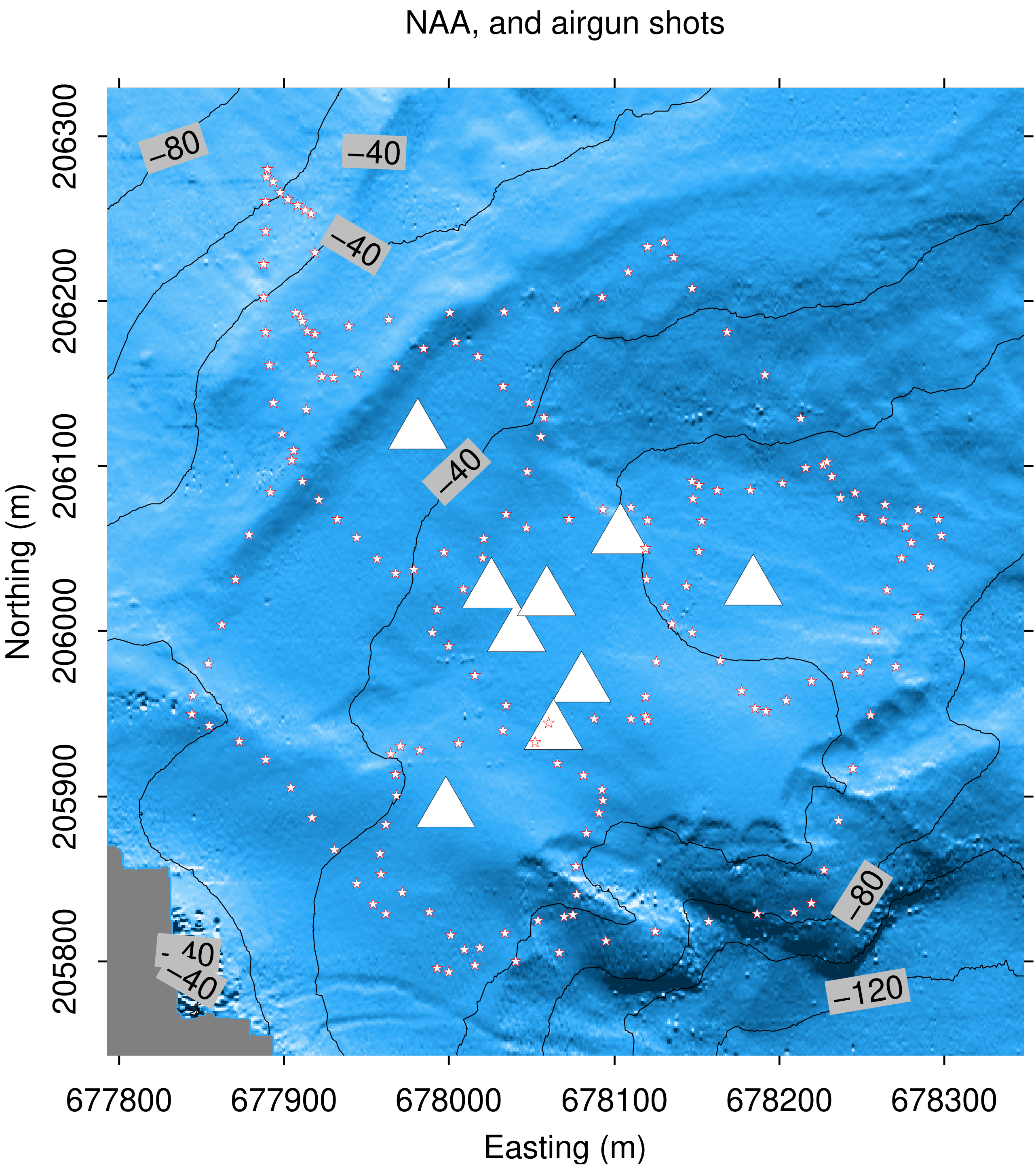

Figure K.2: Red stars indicate the airgun shooting path at NAA (white triangles).

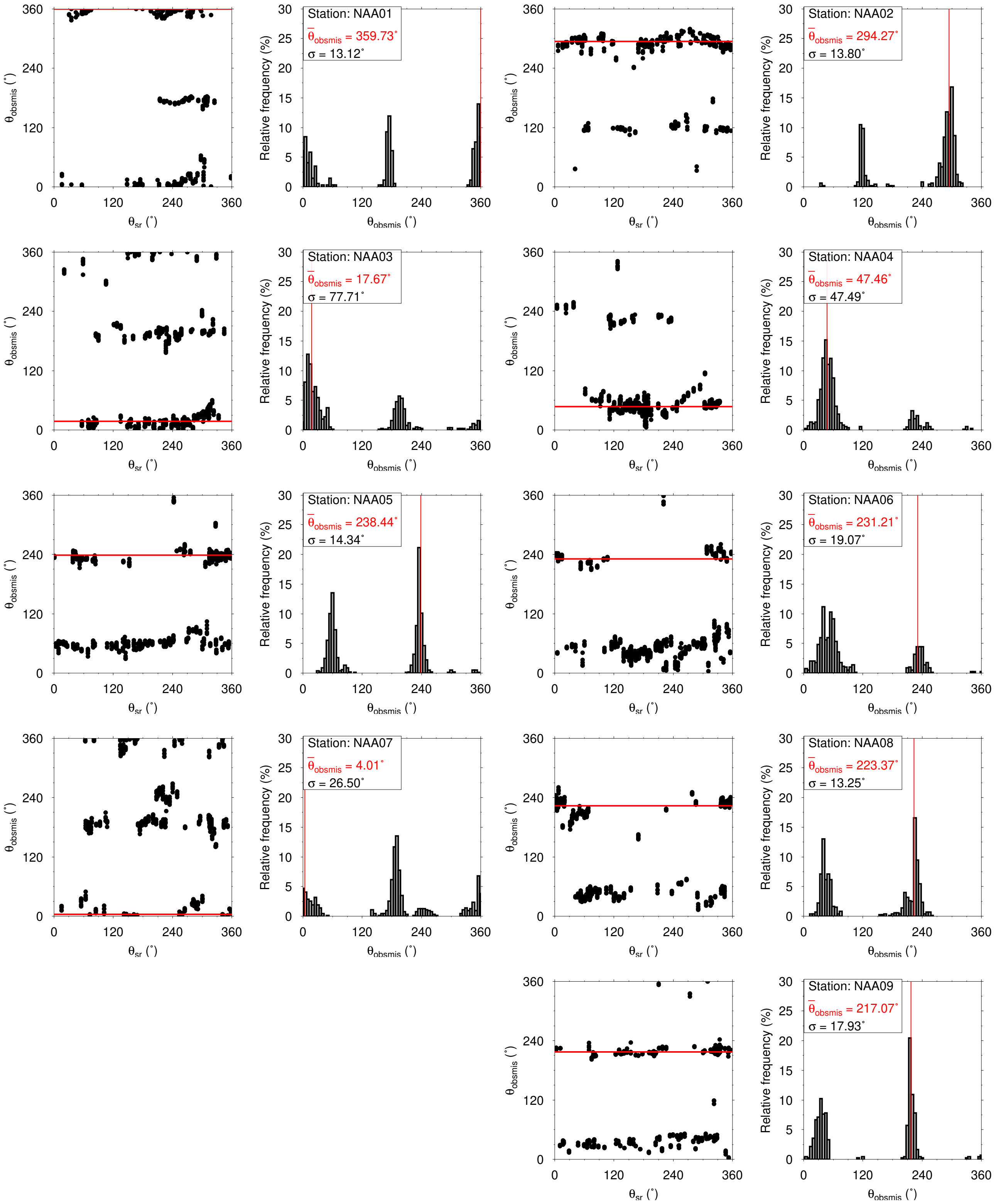

Figure K.3: Misorientation estimates at each OBS station of array NAA with respect to the shot azimuth and the corresponding relative frequency of occurrence.

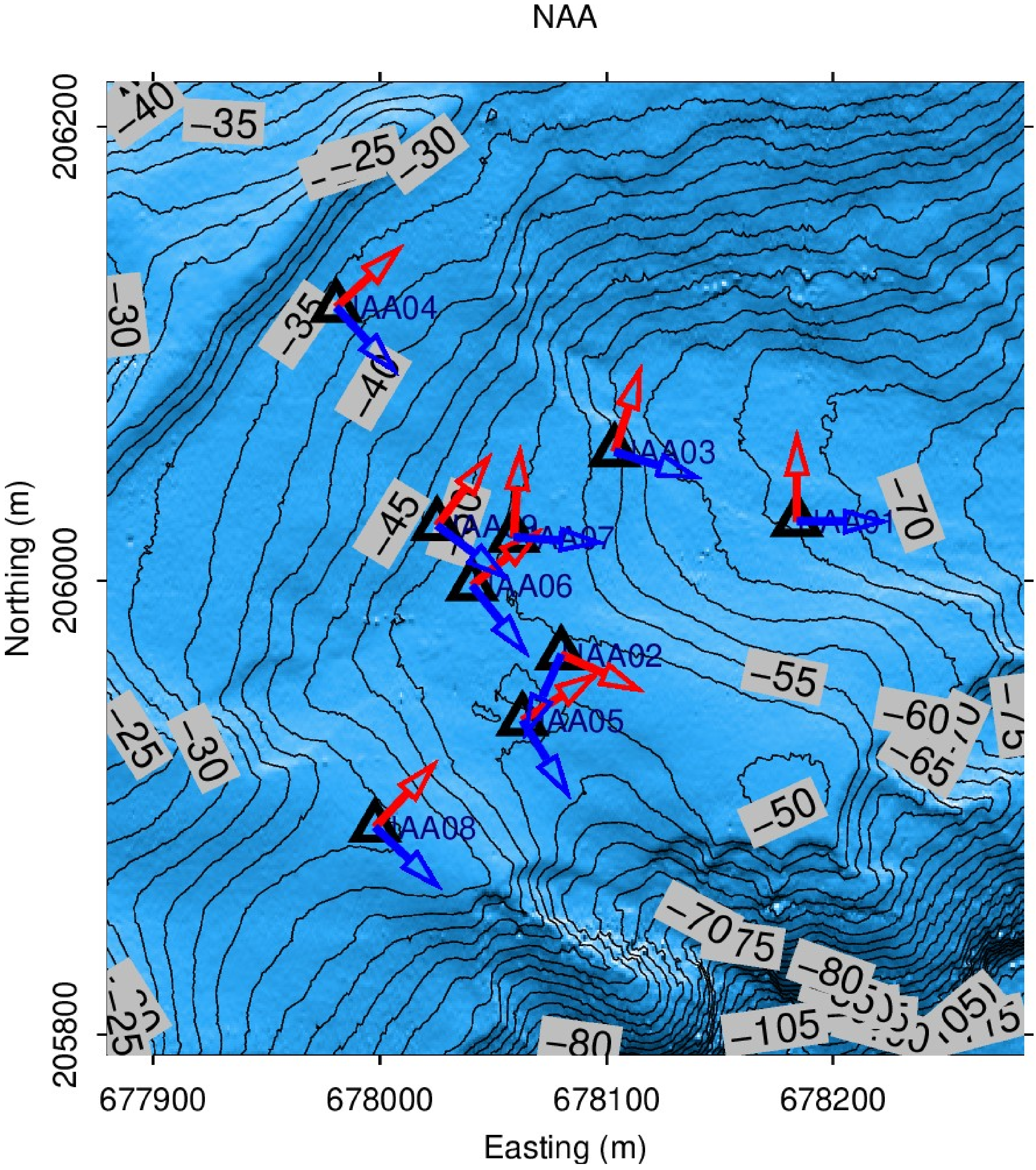

Figure K.4: Plot of the OBS horizontal component orientations on the bathymetry map for OBS stations of array NAA.

L. St. Niklausen: Array NIA

L.1. OBS locations

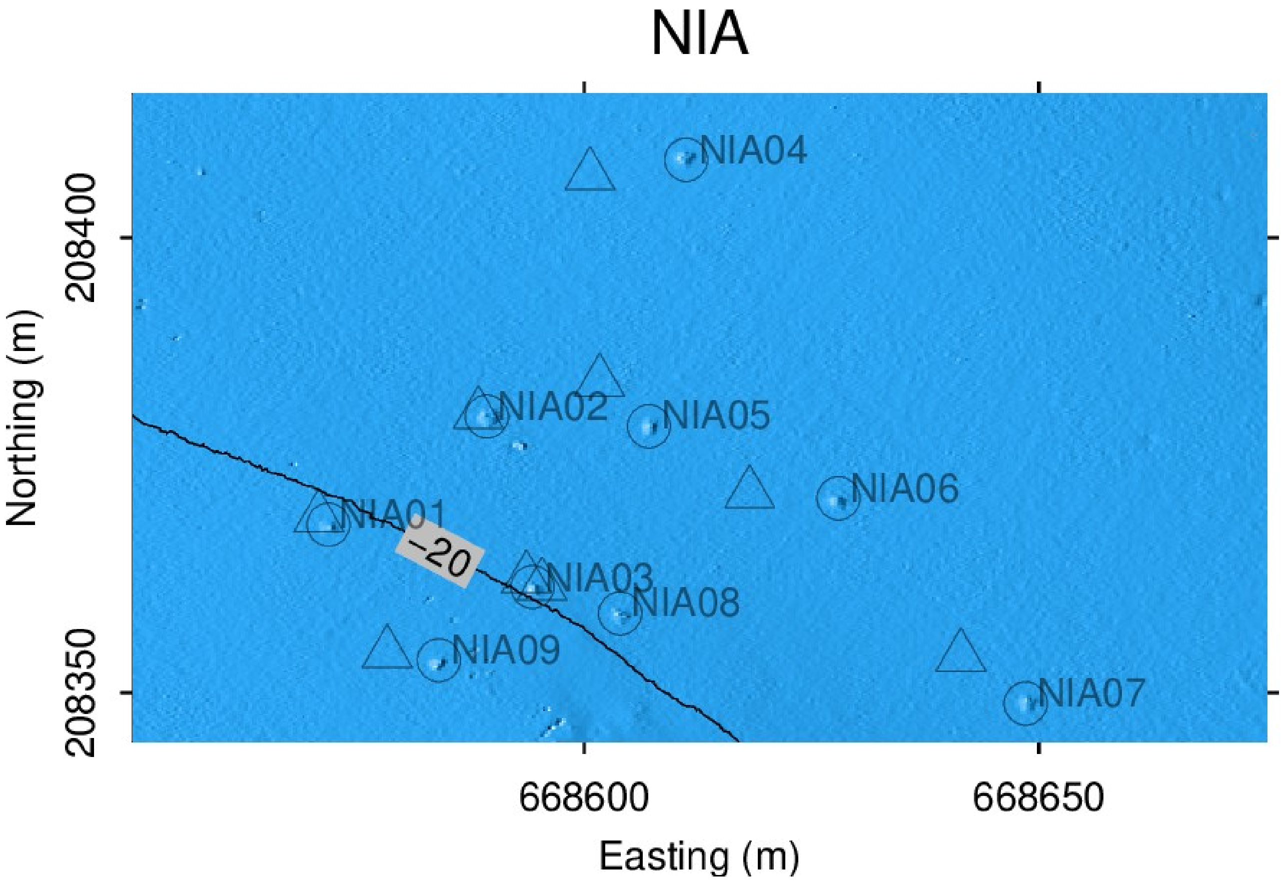

Figure L.1: OBS localization at NIA. The reverse triangle indicates the OBS position at deployment using the differential GPS (dGPS); the triangle indicate the OBS position at recovery using the dGPS; and the circle indicates the OBS position from multibeam.BS localization at CHA, CHB, CIA,

L.2. Airgun measurements

No airgun available at NIA.

L.3. OBS misorientation estimation

No obsmis values available at NIA.

L.4. OBS misorientation with base bathymetry

No obsmis values available at NIA.

M. St. Niklausen: Array NIB

M.1. OBS locations

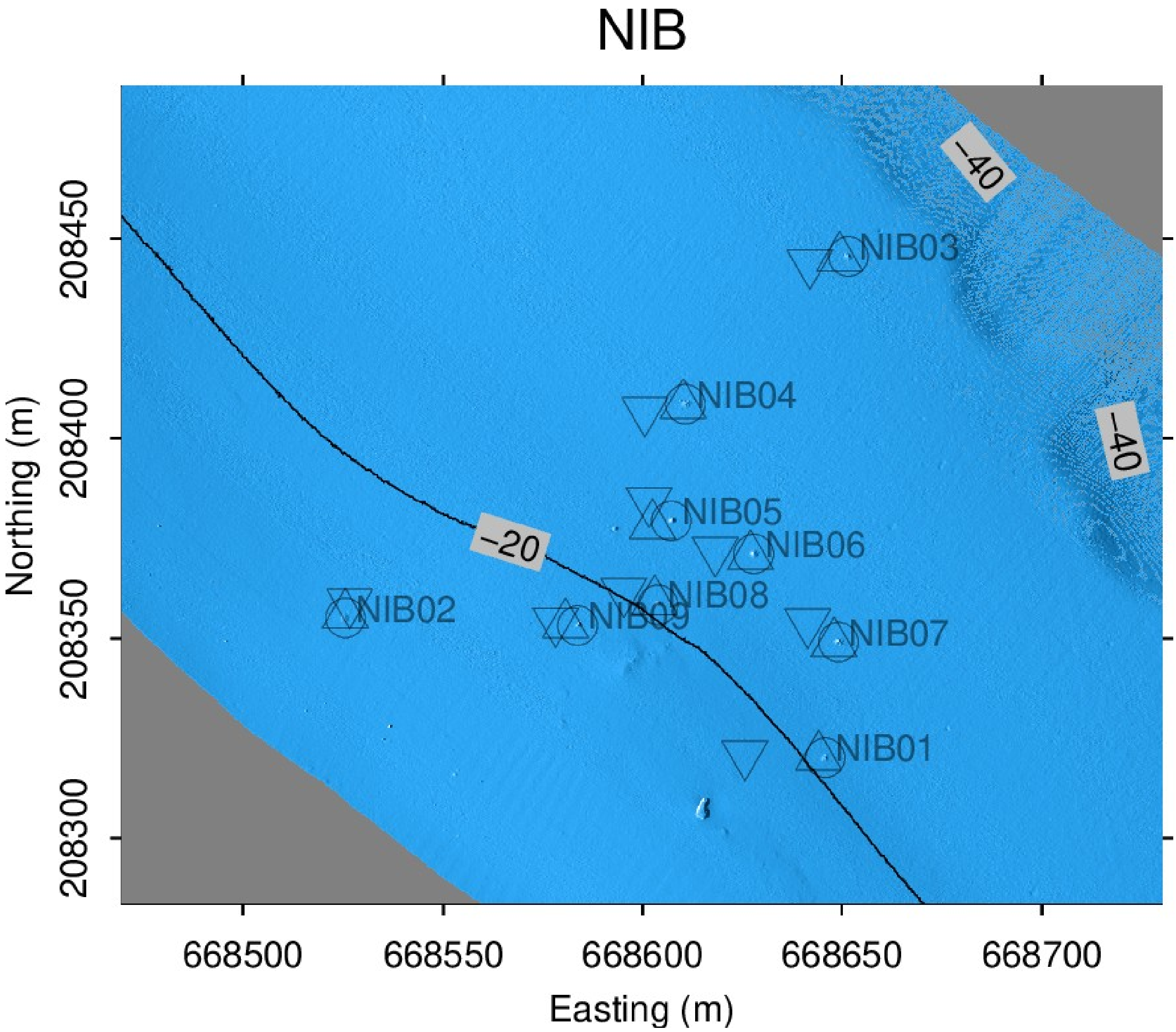

Figure M.1: OBS localization at NIB. The reverse triangle indicates the OBS position at deployment using the differential GPS (dGPS); the triangle indicate the OBS position at recovery using the dGPS; and the circle indicates the OBS position from multibeam.

M.2. Airgun measurements

No airgun available at NIB.

M.3. OBS misorientation estimation

No obsmis values available at NIB.

M.4. OBS misorientation with base bathymetry

No obsmis values available at NIB.

N. St. Niklausen: Array NIC

N.1. OBS locations

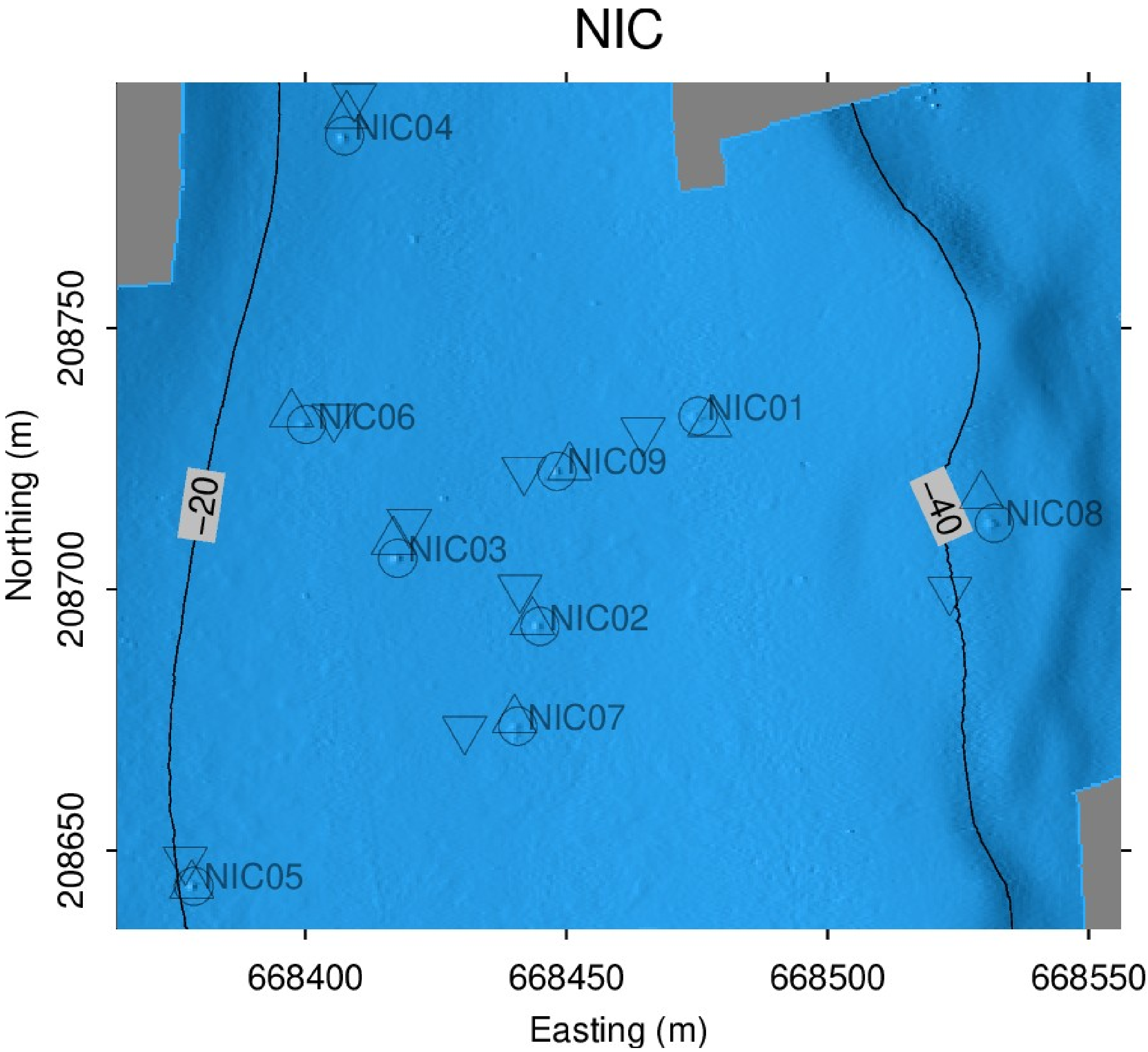

Figure N.1: OBS localization at CIA. The reverse triangle indicates the OBS position at deployment using the differential GPS (dGPS); the triangle indicate the OBS position at recovery using the dGPS; and the circle indicates the OBS position from multibeam.

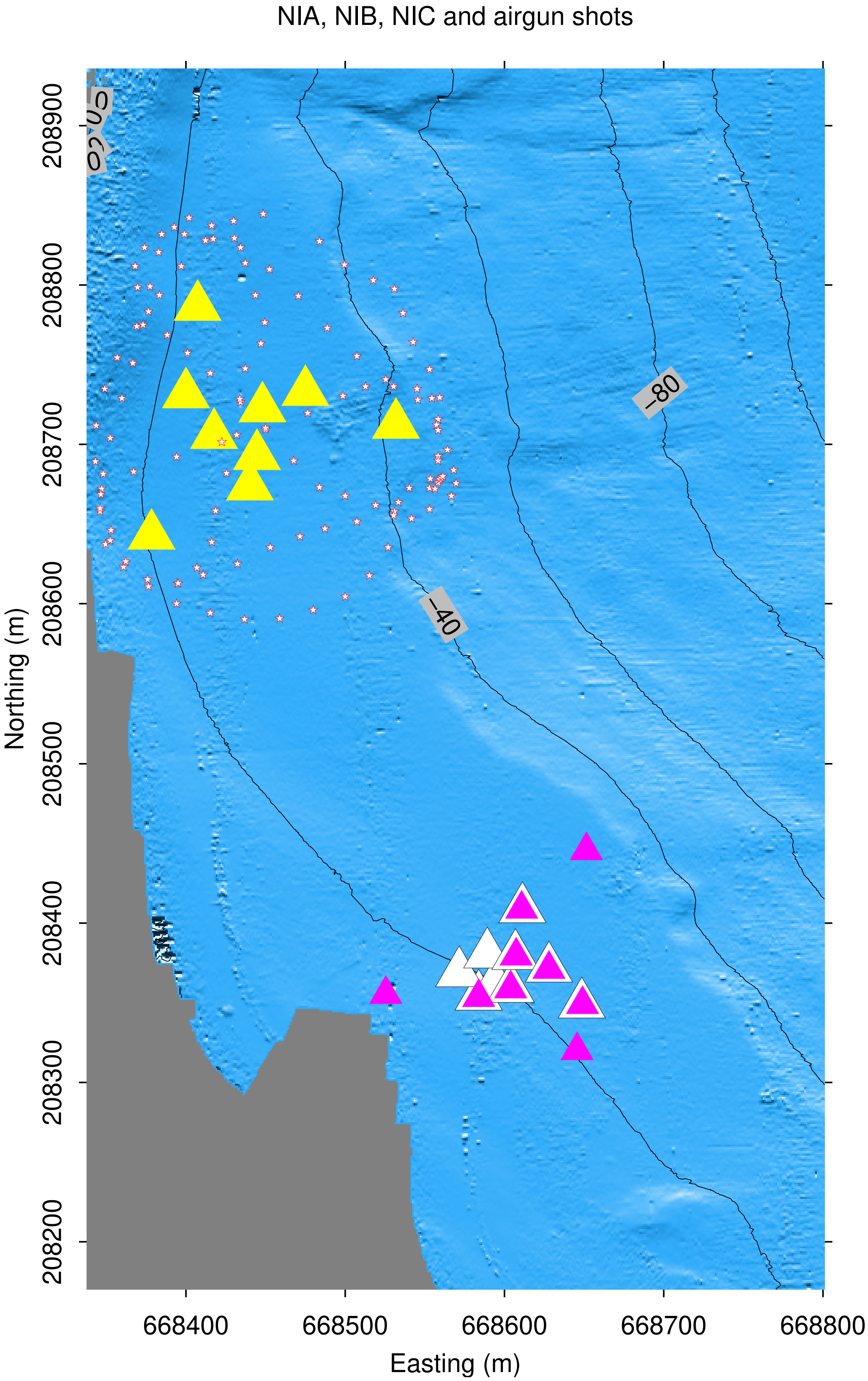

Figure N.2: Red stars indicate the airgun shooting path at NIC (yellow triangles).

N.3. OBS misorientation estimation

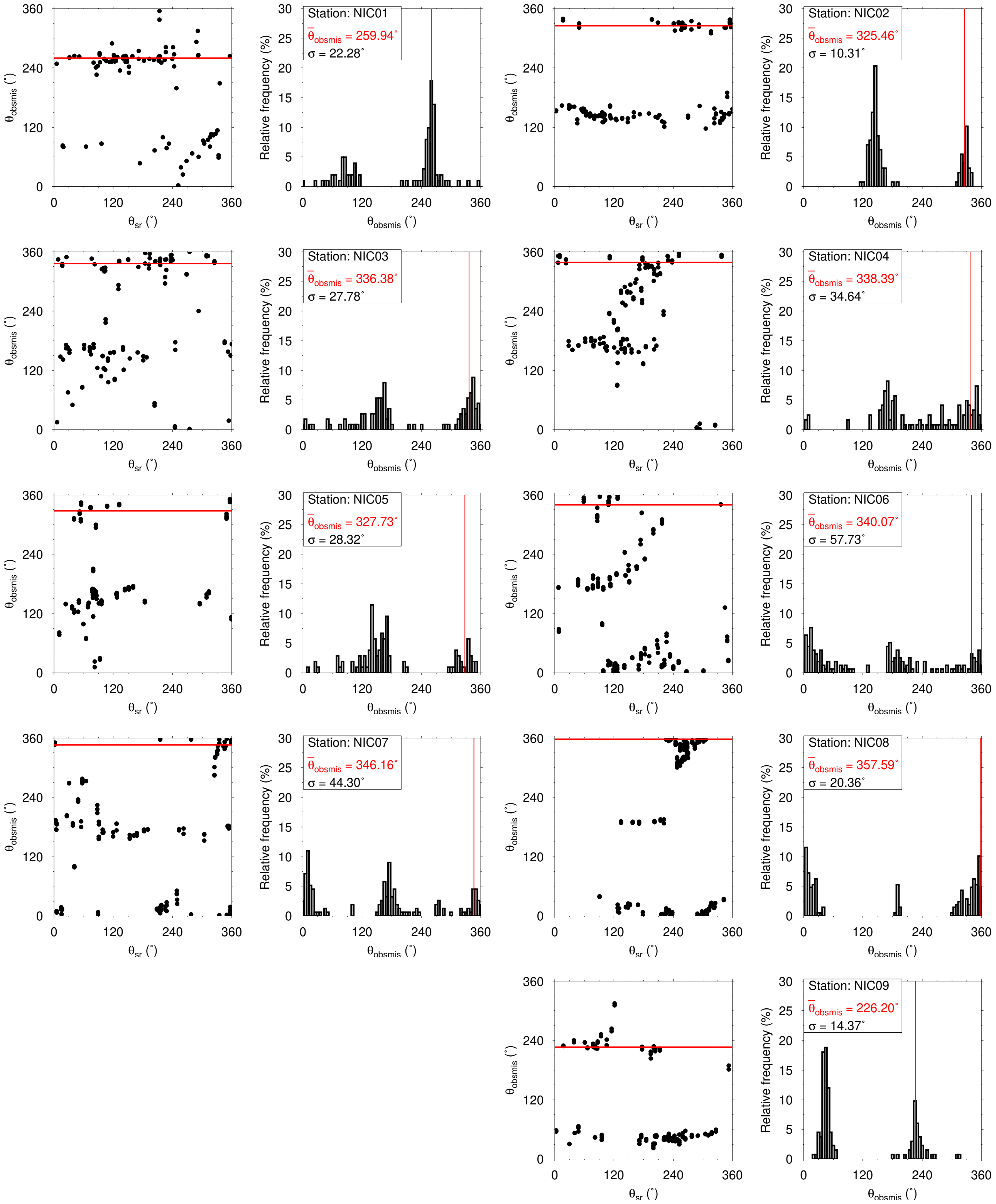

Figure N.3: Misorientation estimates at each OBS station of array NIC with respect to the shot azimuth and the corresponding relative frequency of occurrence.

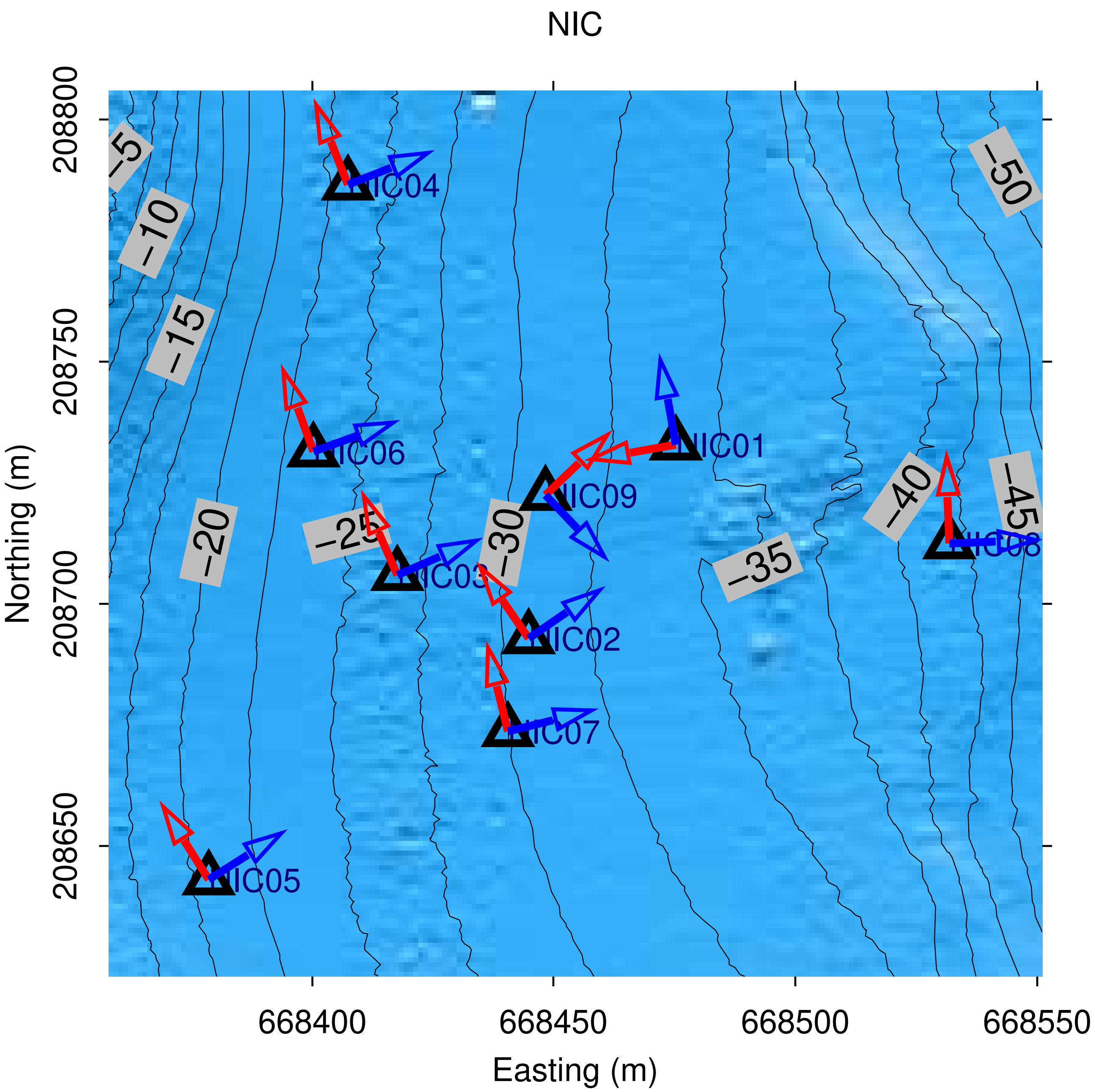

Figure N.4: Plot of the OBS horizontal component orientations on the bathymetry map for OBS stations of array NIC.

O. Weggis: Array WEA

O.1. OBS locations

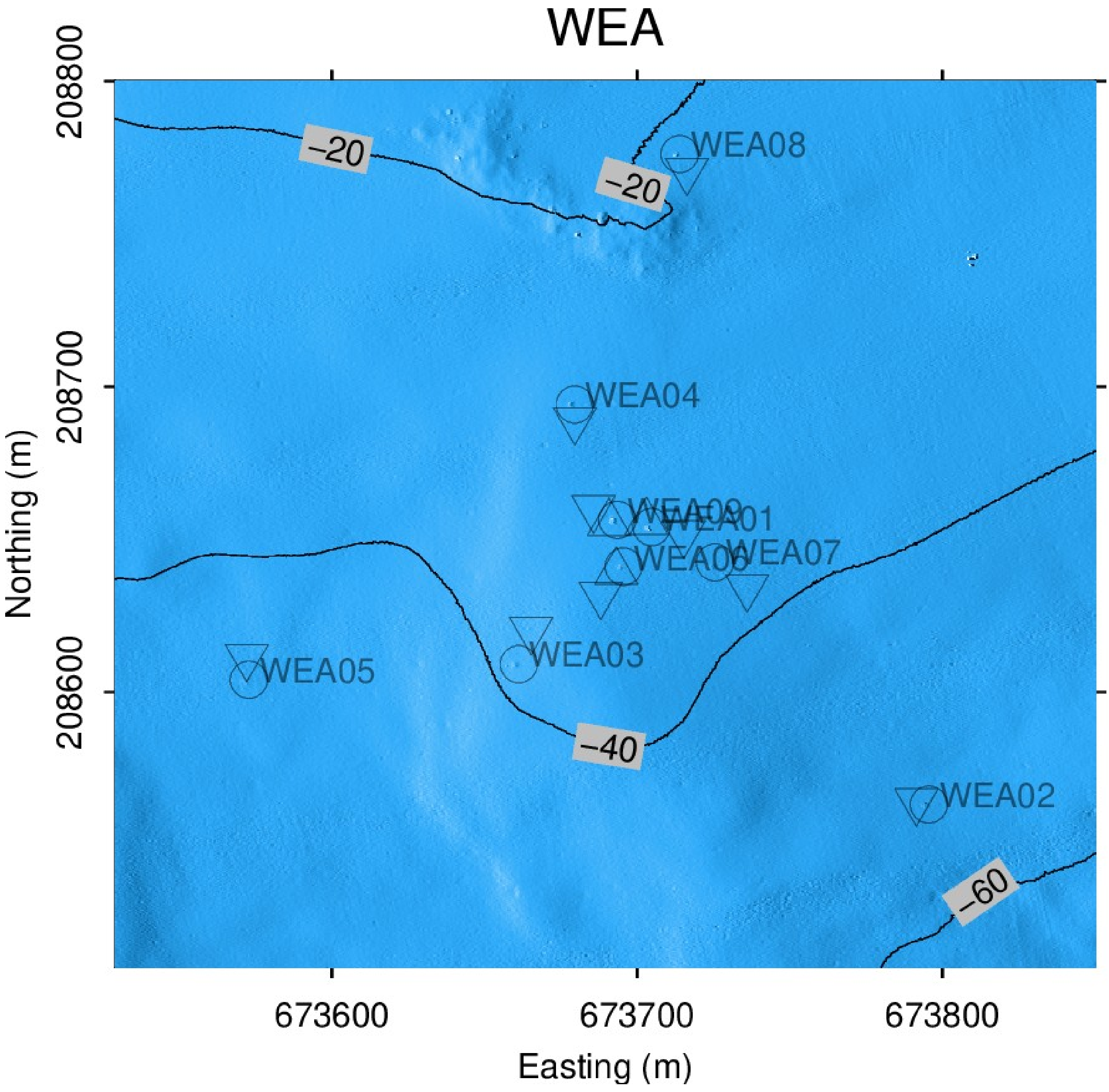

Figure O.1: OBS localization at WEA. The reverse triangle indicates the OBS position at deployment using the differential GPS (dGPS); the triangle indicate the OBS position at recovery using the dGPS; and the circle indicates the OBS position from multibeam.

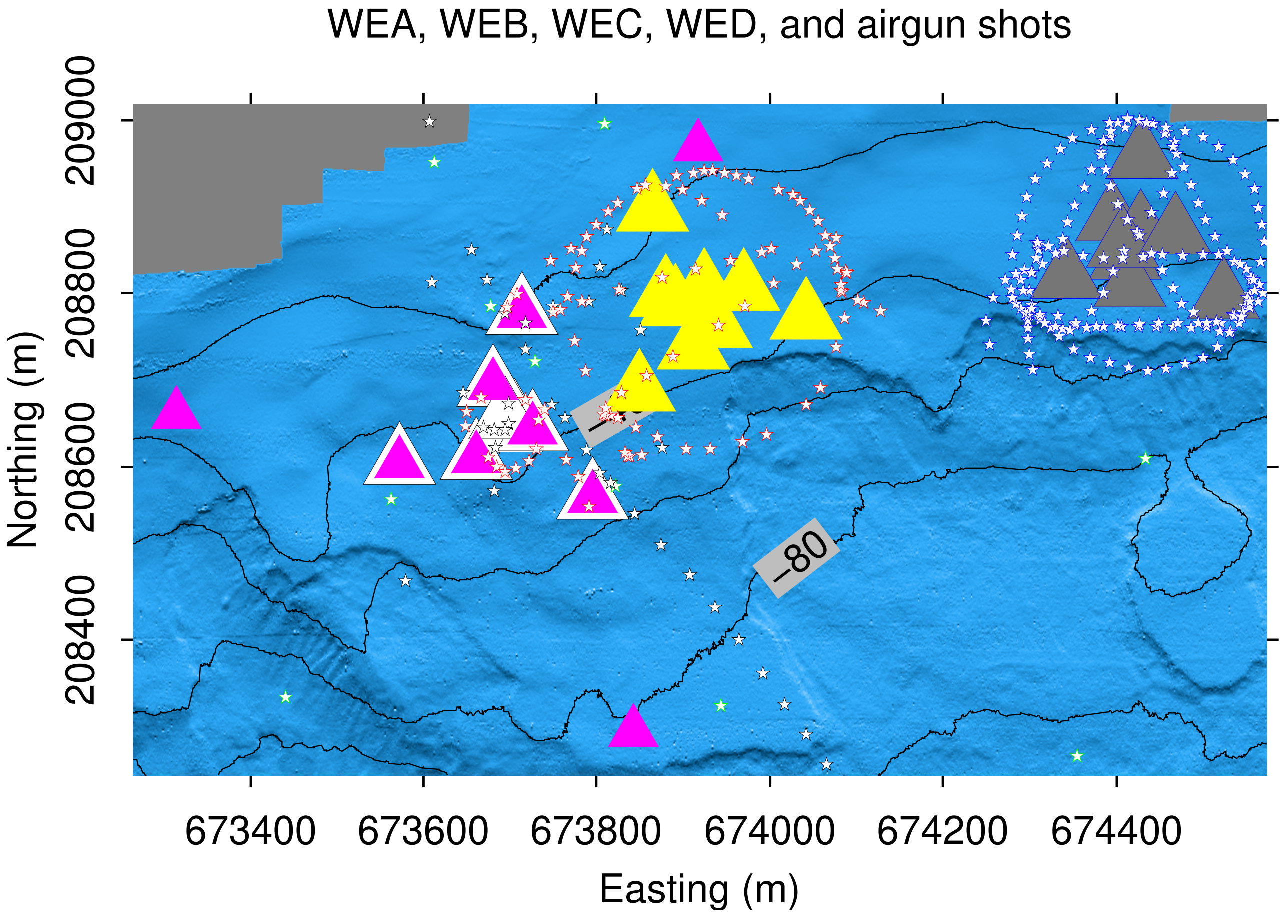

Figure O.2: Green stars indicate the airgun shooting path at WEA (white triangles).

O.3. OBS misorientation estimation

No obsmis values available at WEA due to poor airgun coverage.

O.4. OBS misorientation with base bathymetry

No obsmis values available at WEA due to poor airgun coverage.

P. Weggis: Array WEB

P.1. OBS locations

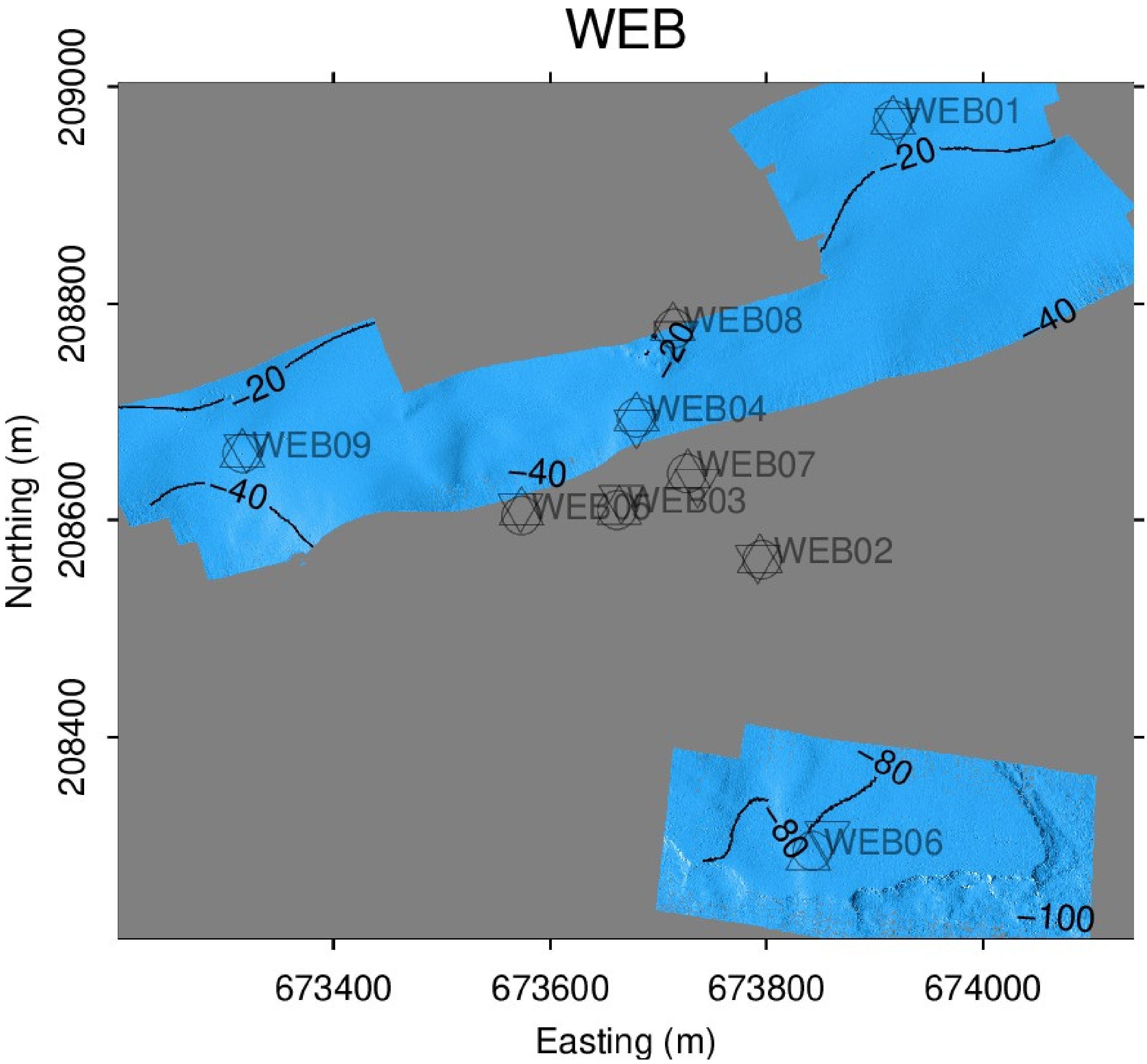

Figure P.1: OBS localization at WEB. The reverse triangle indicates the OBS position at deployment using the differential GPS (dGPS); the triangle indicate the OBS position at recovery using the dGPS; and the circle indicates the OBS position from multibeam.

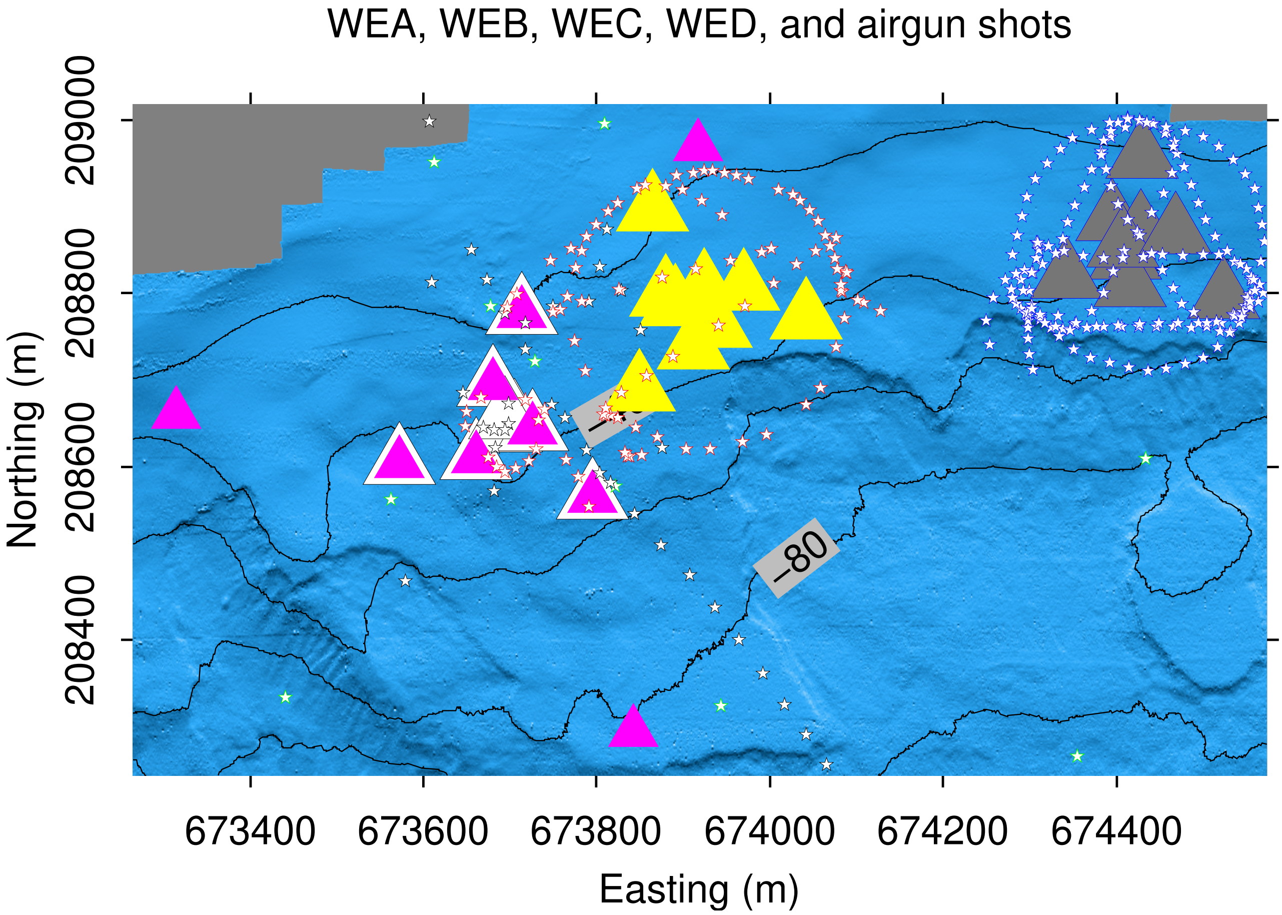

Figure P.2: Black stars indicate the airgun shooting path at WEB (magenta triangles).

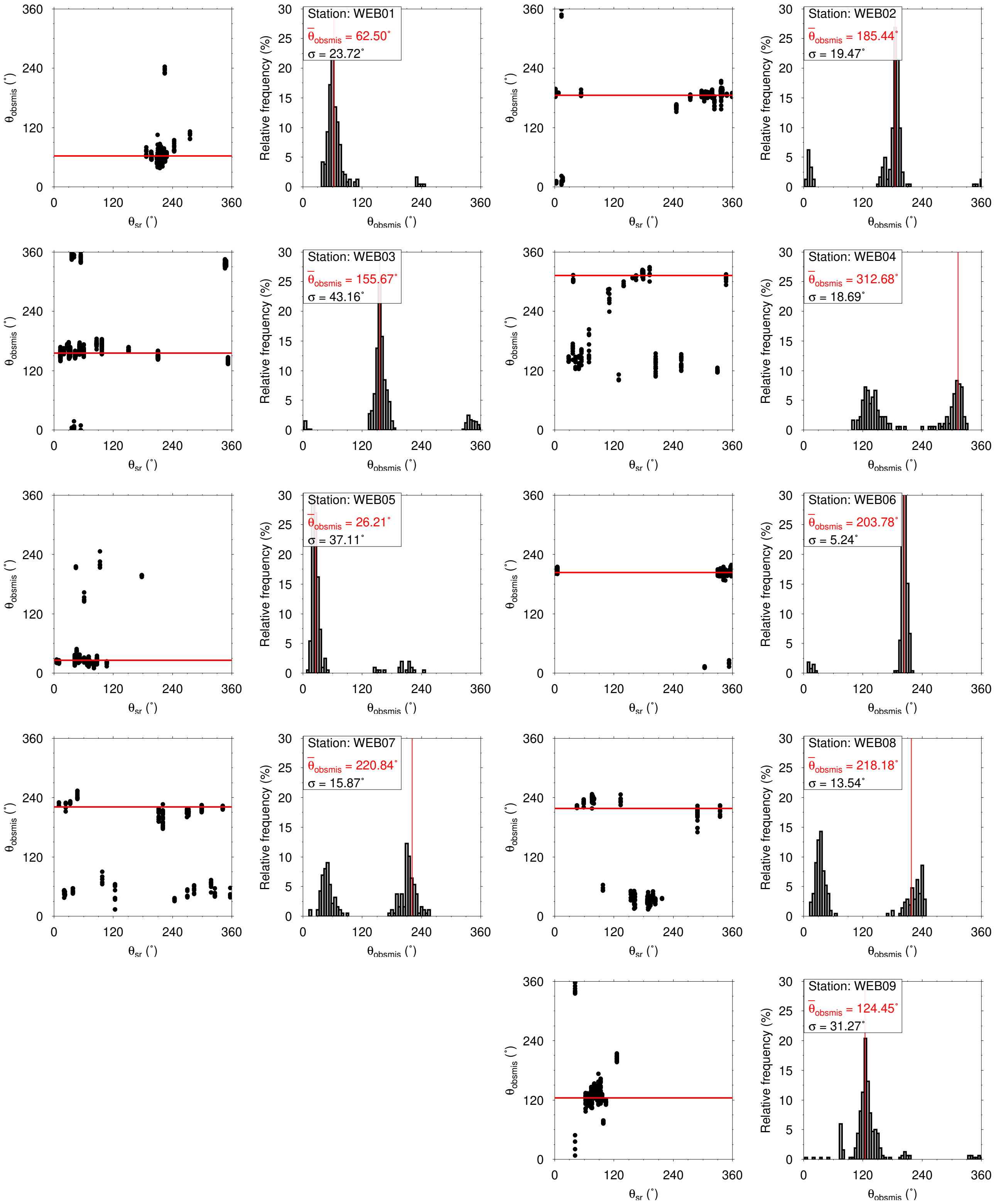

Figure P.3: Misorientation estimates at each OBS station of array WEB with respect to the shot azimuth and the corresponding relative frequency of occurrence.

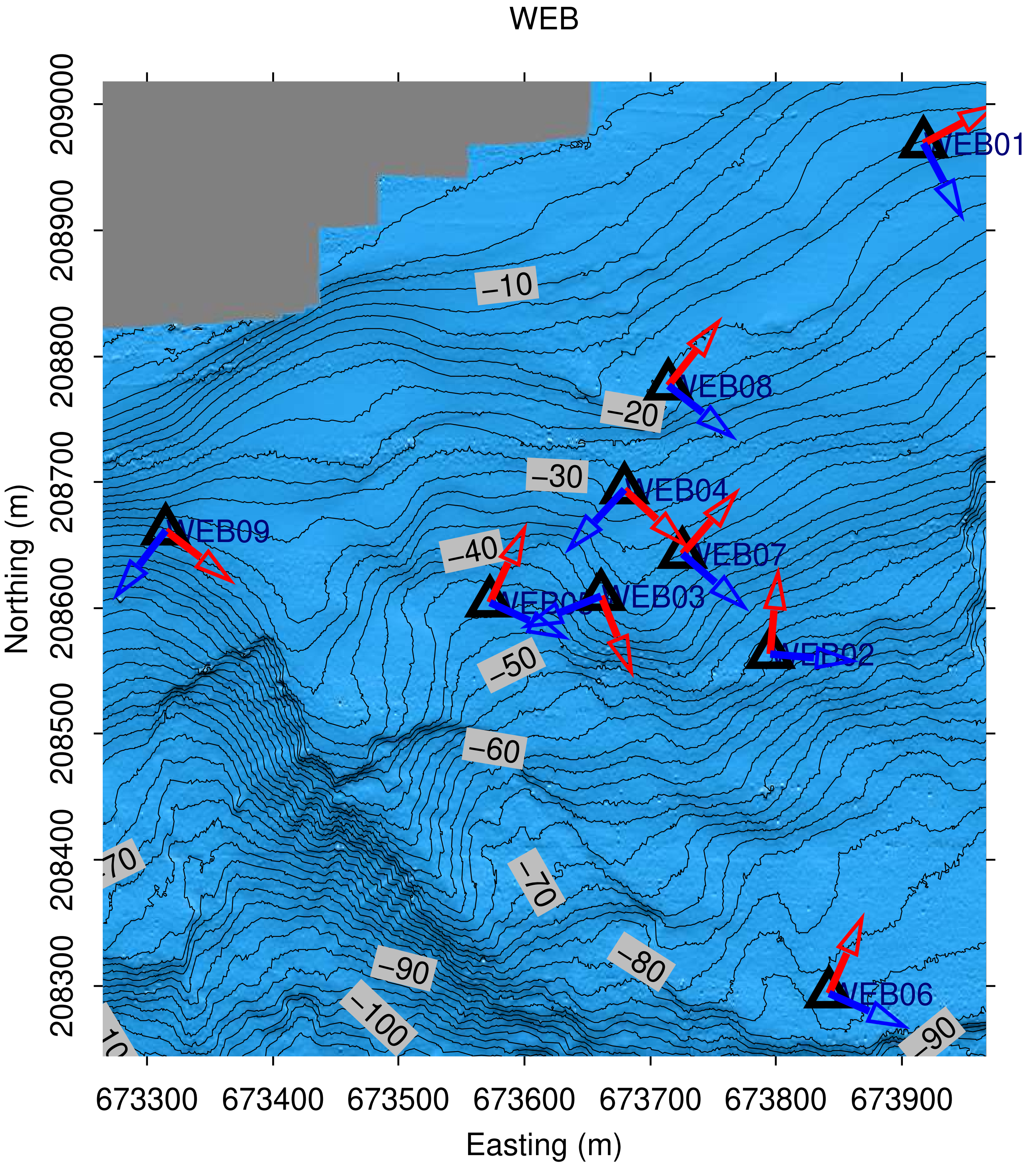

Figure P.4: Plot of the OBS horizontal component orientations on the bathymetry map for OBS stations of array WEB.

Q. Weggis: Array WEC

Q.1. OBS locations

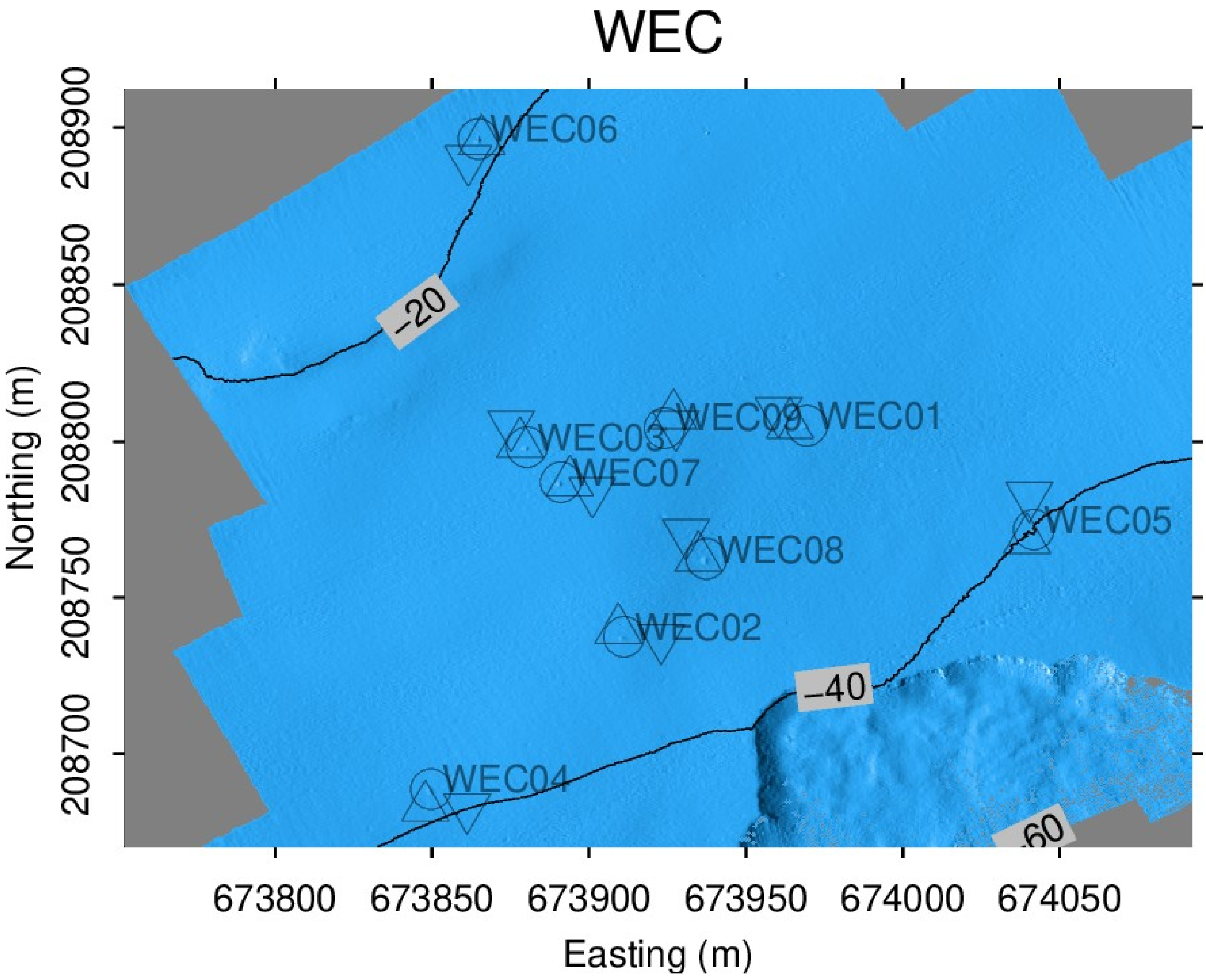

Figure Q.1: OBS localization at WEC. The reverse triangle indicates the OBS position at deployment using the differential GPS (dGPS); the triangle indicate the OBS position at recovery using the dGPS; and the circle indicates the OBS position from multibeam.

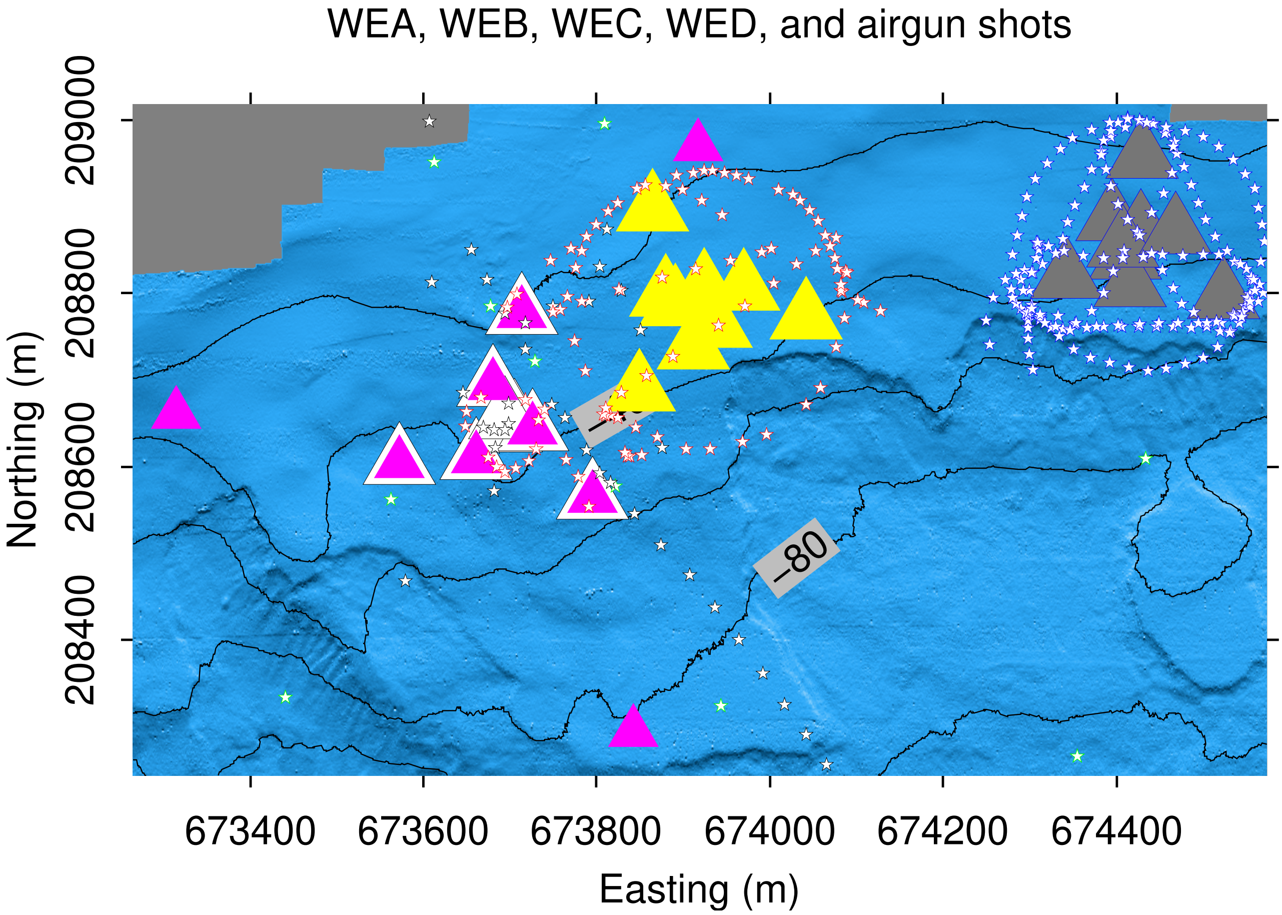

Figure Q.2: Red stars indicate the airgun shooting path at WEC (yellow triangles)

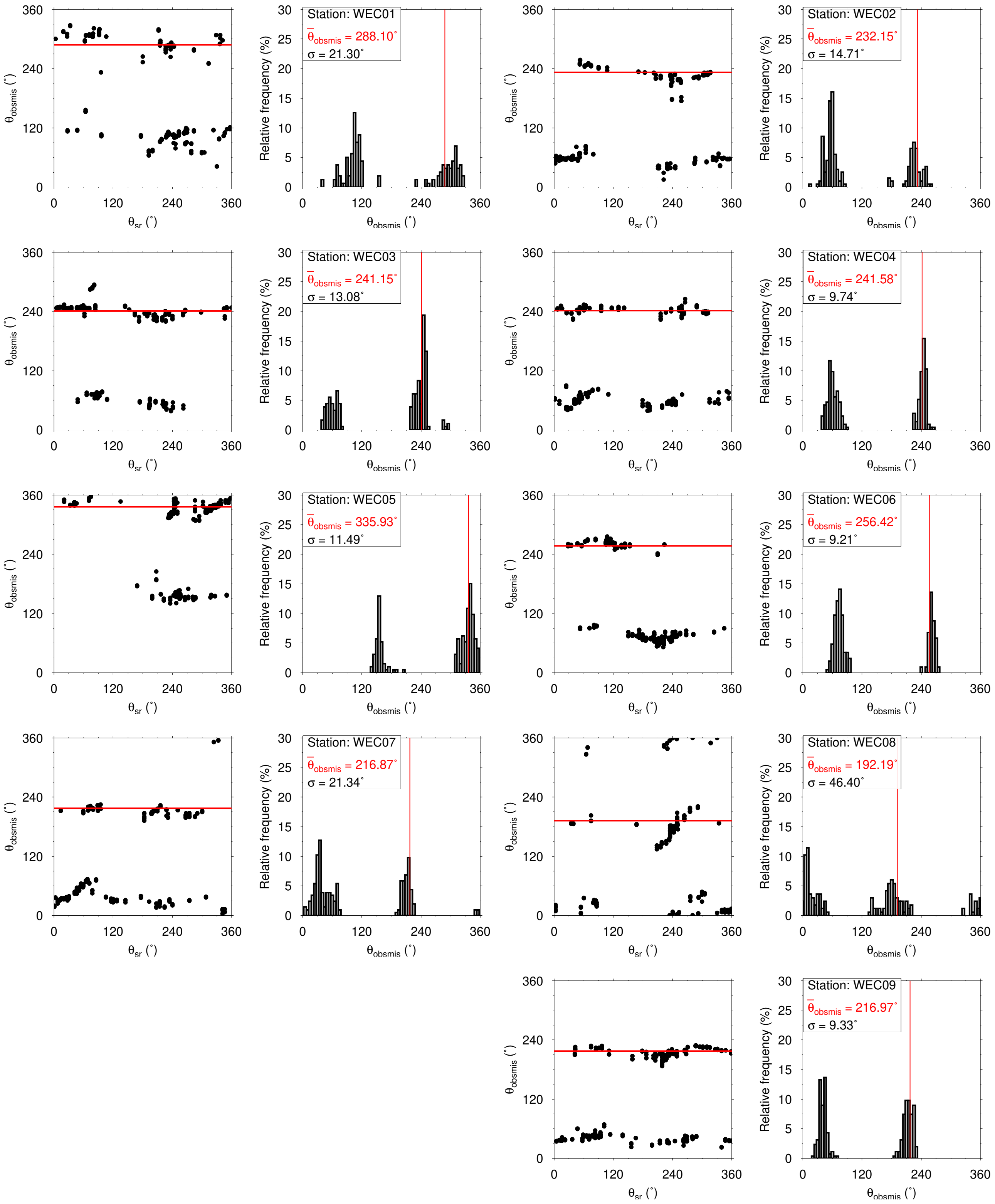

Figure Q.3: Misorientation estimates at each OBS station of array WEC with respect to the shot azimuth and the corresponding relative frequency of occurrence.

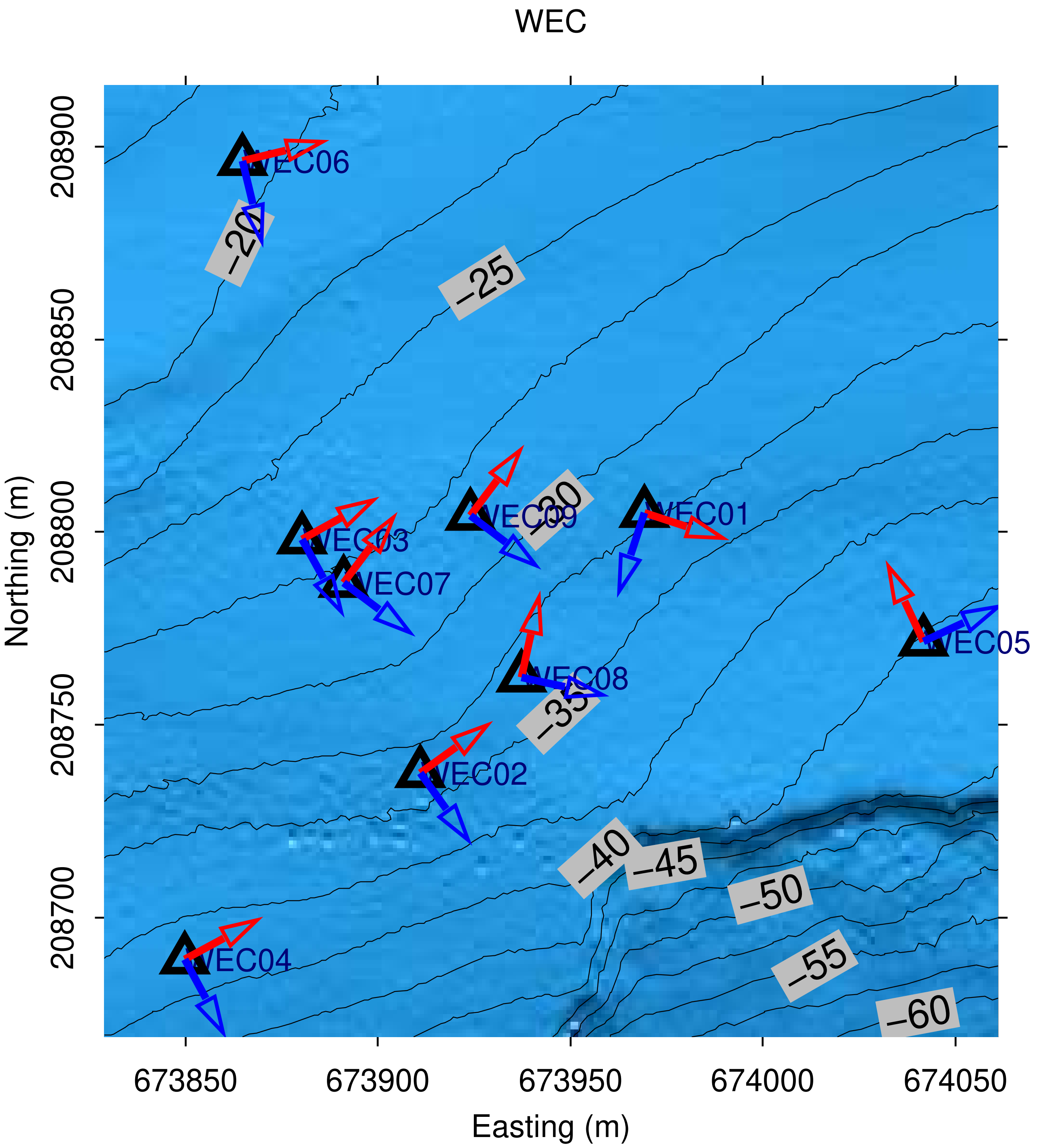

Figure Q.4: Plot of the OBS horizontal component orientations on the bathymetry map for OBS stations of array CHB.

R. Weggis: Array WED

R.1. OBS locations

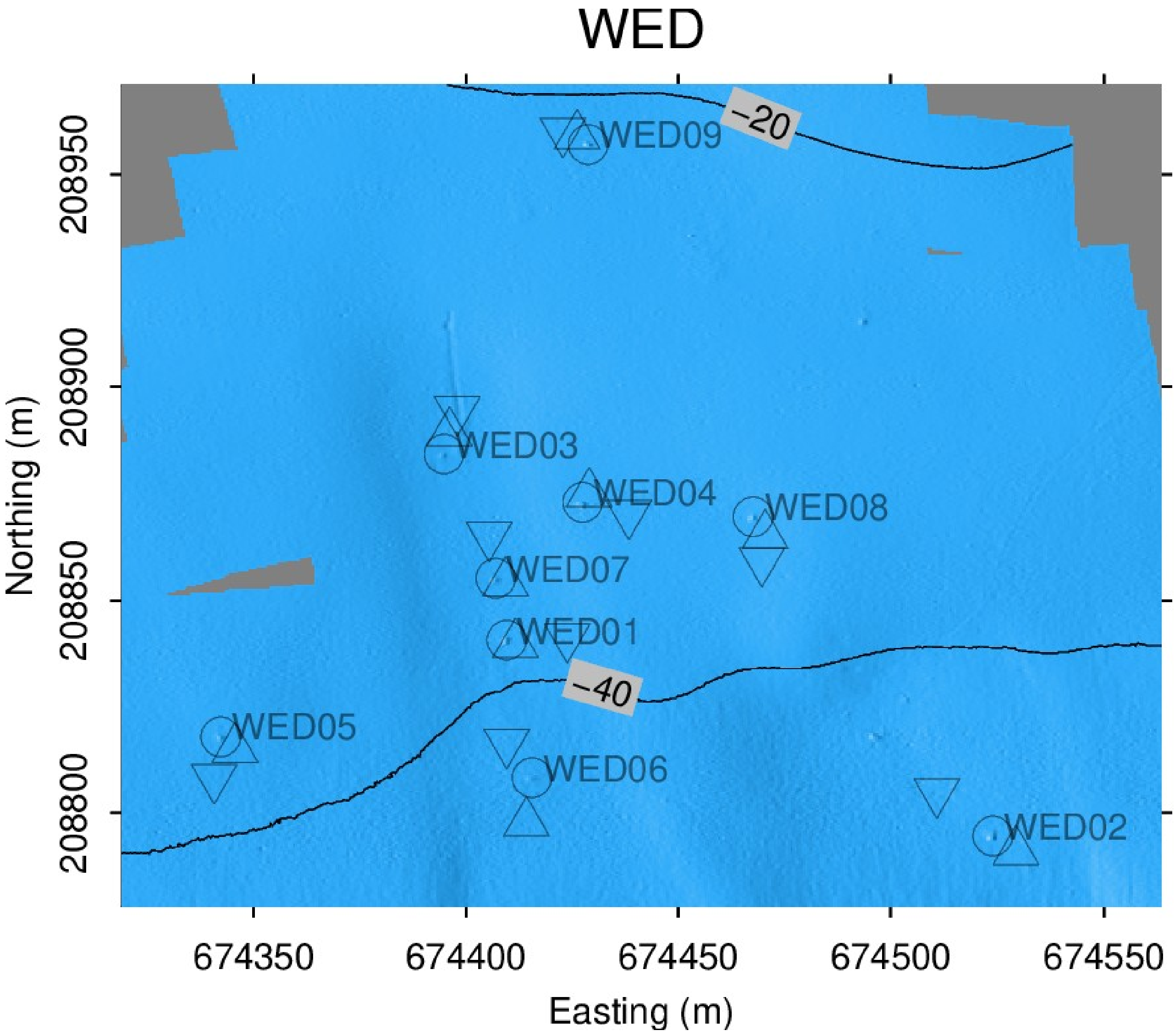

Figure R.1: OBS localization at WED. The reverse triangle indicates the OBS position at deployment using the differential GPS (dGPS); the triangle indicate the OBS position at recovery using the dGPS; and the circle indicates the OBS position from multibeam.

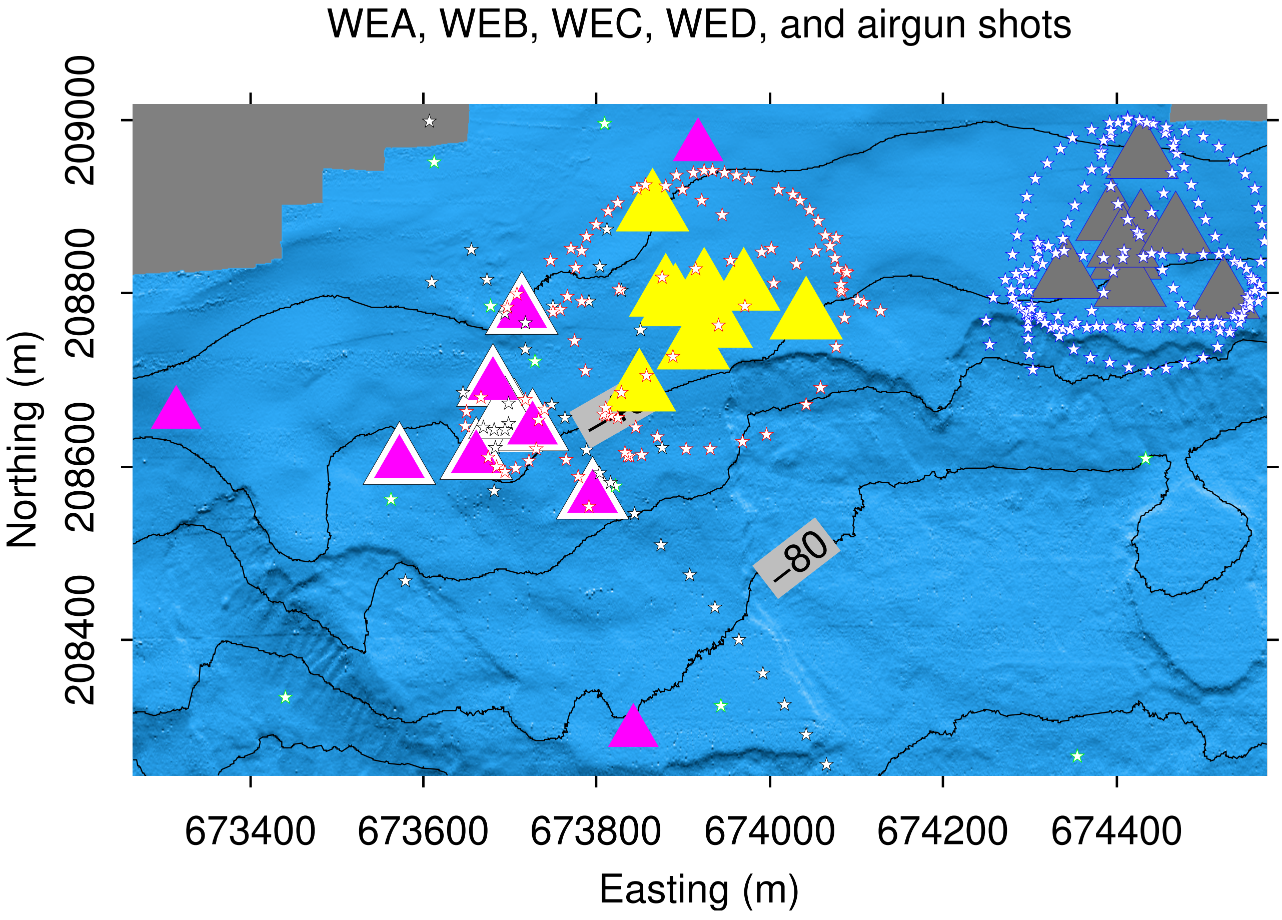

Figure R.2: Blue stars indicate the airgun shooting path at WED (gray triangles).

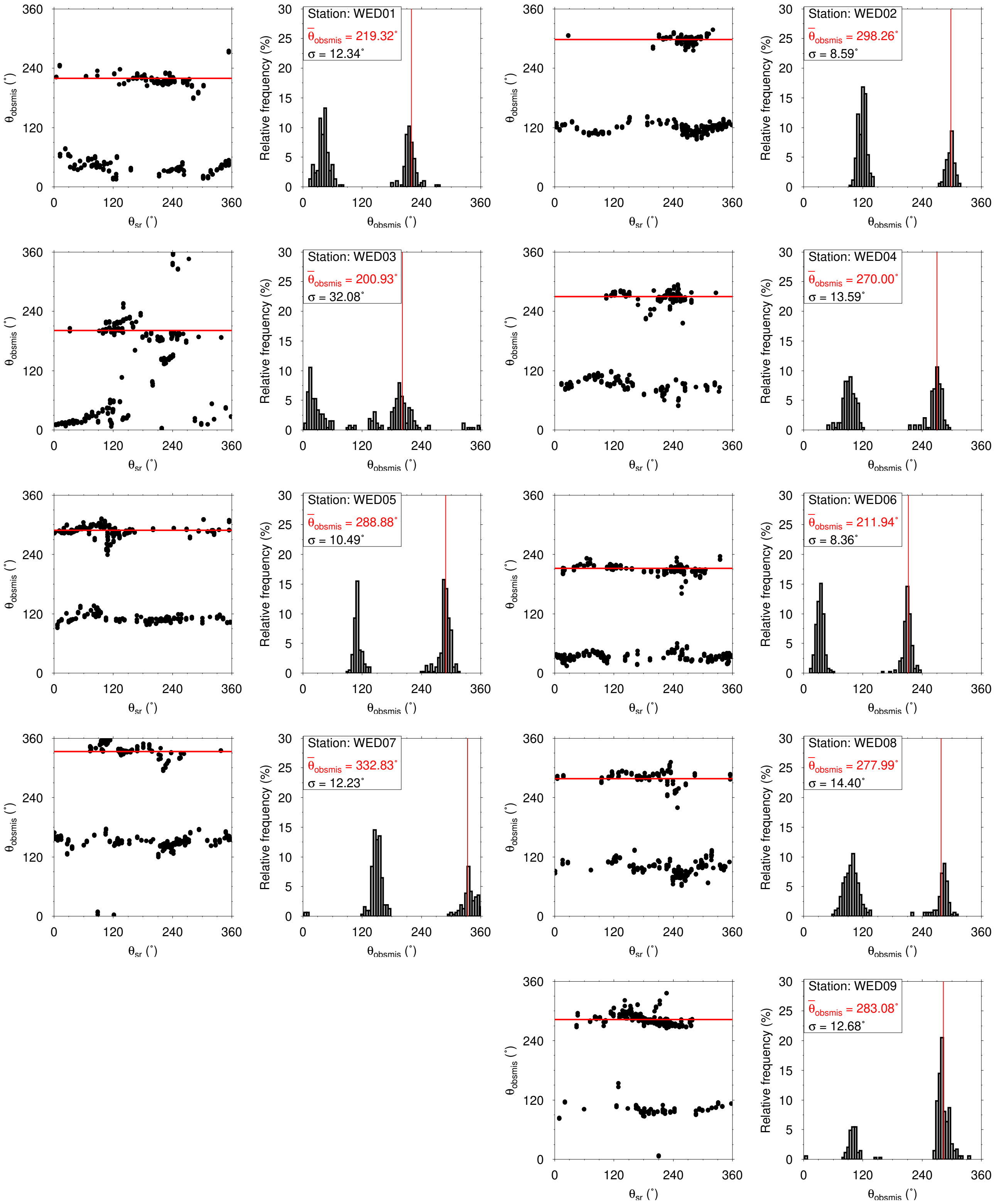

Figure R.3: Misorientation estimates at each OBS station of array WED with respect to the shot azimuth and the corresponding relative frequency of occurrence.

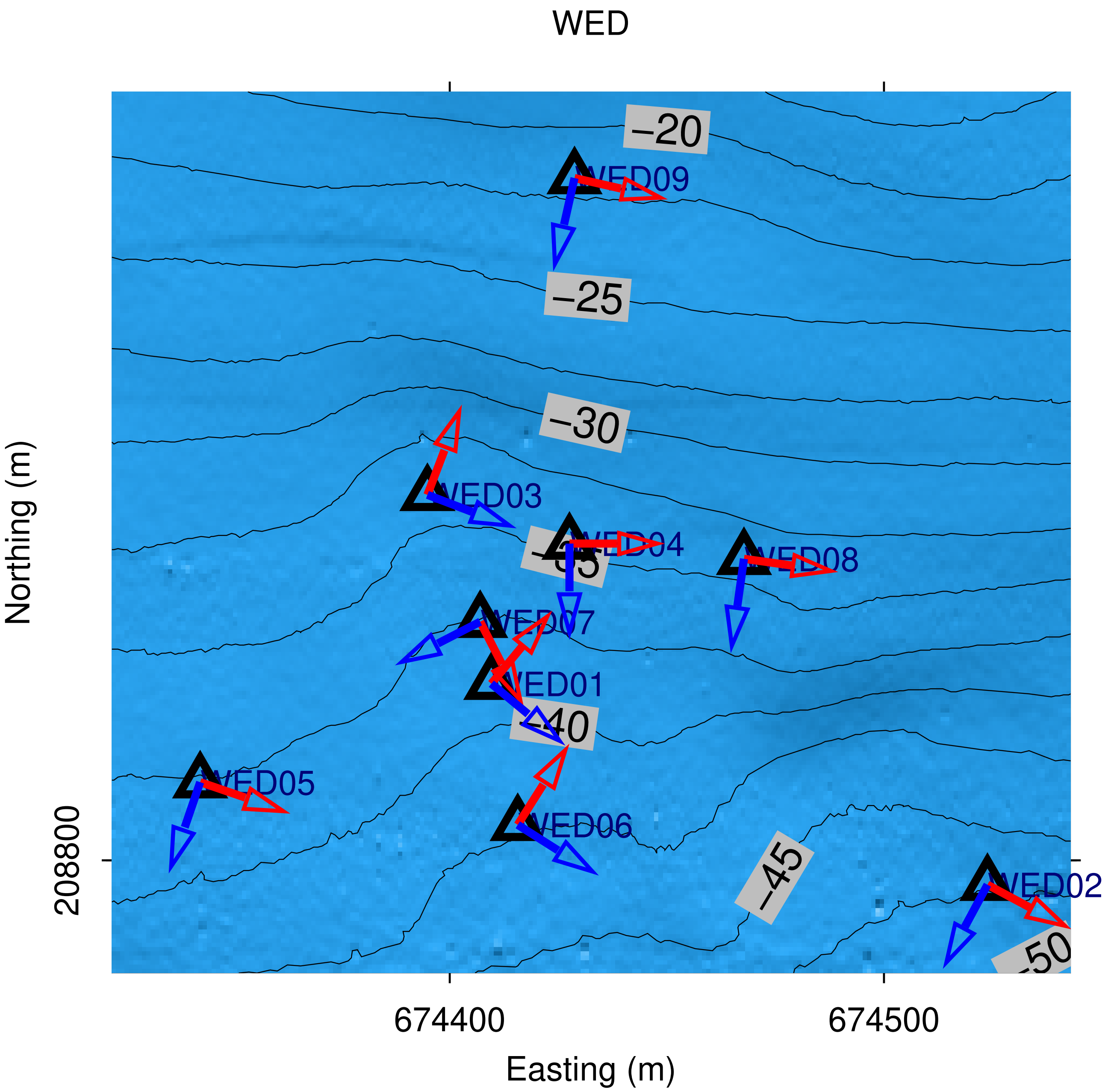

Figure R.4: Plot of the OBS horizontal component orientations on the bathymetry map for OBS stations of array CHB.

S. Modeling Scholte and Love waves phase velocity dispersion curves

Table S.1 gives the seismic parameters used for the modeling of the Scholte and Love waves phase velocity. The results are presented in Figure S.1.

Table S.1: Two-layer over half space model. The first layer is a water layer and the second layer is a soft sediment layer with water saturated P-wave velocity.

Thickness (m)	V_P (m/s)	V_P (m/s)	Mass density (Kg/m^3)
50	1000	0	1000
25	1700	200	1900
∞	2000	1000	2500

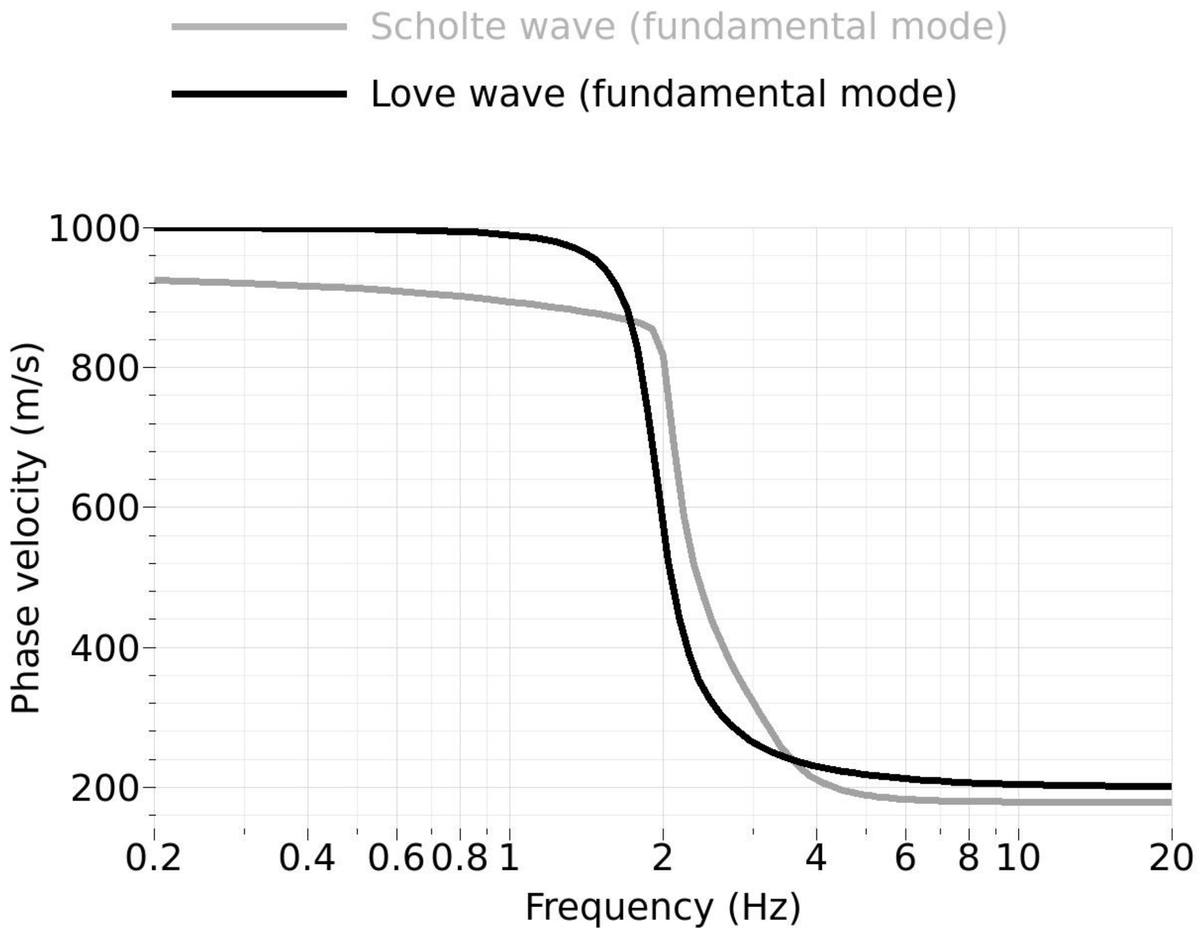

Figure S.1: Comparison between Scholte and Love wave phase velocity dispersion curve for a simple one layer over a half space.

T. Comparison between Scholte and Love waves phase velocity at CIA, ENA, ENB, and MUA

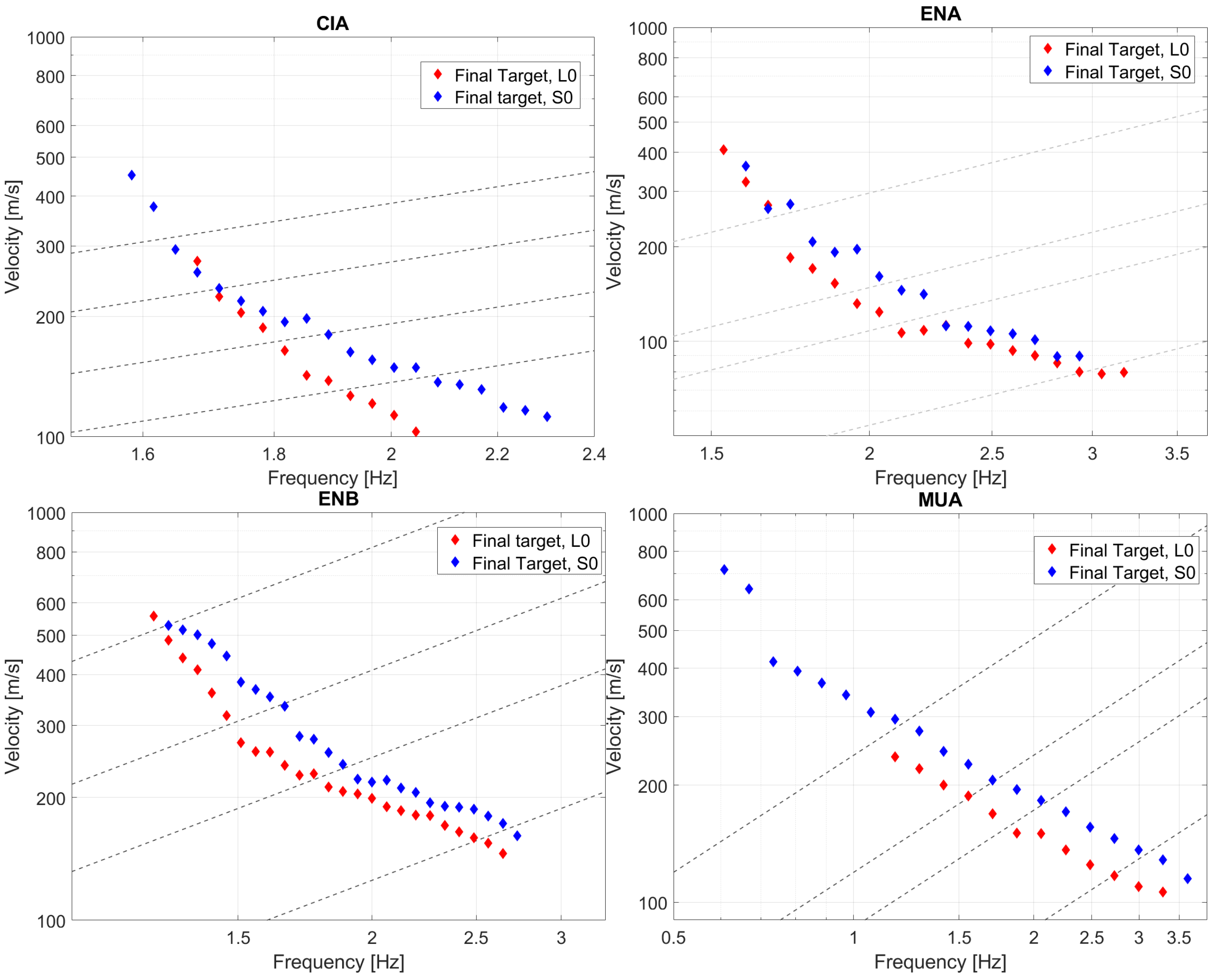

Figure T.1: Comparison between Scholte and Love wave phase velocity dispersion curve at CIA, ENA, ENB, and MUA.
